# Design, Synthesis, and Biological Evaluation of the Quorum-Sensing Inhibitors of *Pseudomonas aeruginosa* PAO1

**DOI:** 10.3390/molecules29102211

**Published:** 2024-05-08

**Authors:** Xinlin Yan, Shi Hou, Cheng Xing, Yuanyuan Zhang, Jiajia Chang, Junhai Xiao, Feng Lin

**Affiliations:** 1 National Engineering Research Center for the Emergency Drug, State Key Laboratory of Toxicology and Medical Countermeasures, Beijing Institute of Pharmacology and Toxicology, Beijing 100850, China; yanxinlin11@163.com (X.Y.); houshi28@126.com (S.H.); jiajiachang@126.com (J.C.); 2 School of Pharmaceutical Sciences, Jilin University, Changchun 130021, China; xingcheng21@mails.jlu.edu.cn; 3 School of Life Sciences, Jilin University, Changchun 130012, China; yuanyuanz21@mails.jlu.edu.cn

**Keywords:** quorum sensing, *Pseudomonas aeruginosa*, inhibitors

## Abstract

Due to the resistance of Gram-negative bacteria *Pseudomonas aeruginosa* PAO1 to most clinically relevant antimicrobials, the use of traditional antibiotic treatments in hospitals is challenging. The formation of biofilms, which is regulated by the quorum-sensing (QS) system of *Pseudomonas aeruginosa* (PA), is an important cause of drug resistance. There are three main QS systems in *P. aeruginosa*: the *las* system, the *rhl* system, and the *pqs* system. The inhibitors of the *las* system are the most studied. Previously, the compound **AOZ-1** was found to have a certain inhibitory effect on the *las* system when screened. In this study, twenty-four compounds were designed and synthesized by modifying the Linker and Rings of **AOZ-1**. Using *C. violaceum* CV026 as a reporter strain, this study first assessed the inhibitory effects of new compounds against QS, and their SAR was investigated. Then, based on the SAR analysis of compound **AOZ-1** derivatives, the parent core of AOZ-1 was replaced to explore the structural diversity. Then, nine new compounds were designed and synthesized with a new nucleus core component of 3-amino-tetrahydro-l,3-oxazin-2-one. The compound **Y-31** (IC_50_ = 91.55 ± 3.35 µM) was found to inhibit the QS of *C. violaceum* CV026. Its inhibitory effect on *C. violaceum* CV026 was better than that of compound **AOZ-1** (IC_50_ > 200 µM). Furthermore, biofilm formation is one of the important causes of *Pseudomonas aeruginosa* PAO1 resistance. In this study, it was found that compound **Y-31,** with a new nucleus core component of 3-amino-tetrahydro-l,3-oxazin-2-one, had the highest biofilm inhibition rate (40.44%). The compound **Y-31** has a certain inhibitory effect on the production of PAO1 virulence factors (pyocyanin, rhamnolipid, and elastase) and swarming. When the concentration of compound **Y-31** was 162.5 µM, the inhibition rates of pyocyanin, rhamnolipid, and elastase were 22.48%, 6.13%, and 22.67%, respectively. In vivo, the lifetime of wildtype *Caenorhabditis elegans* N2 infected with *P. aeruginosa* PAO1 was markedly extended by the new parent nucleus **Y-31**. This study also performed cytotoxicity experiments and in vivo pharmacokinetics experiments on the compound **Y-31**. In conclusion, this study identified a compound, **Y-31**, with a new nucleus core component of 3-amino-tetrahydro-l,3-oxazin-2-one, which is a potential agent for treating *P. aeruginosa* PAO1 that is resistant to antibiotics and offers a way to discover novel antibacterial medications.

## 1. Introduction

In recent years, antibiotic-resistant bacteria have emerged due to antibiotic misuse, hindering the treatment of bacterial infections in clinical settings [1]. At least 65% of these diseases caused by bacterial infections are associated with biofilm formation regulated by bacterial quorum sensing [2]. Quorum sensing (QS) is a signal communication mechanism between cells that controls the production, release, and recognition response of signal molecules to regulate the formation of biofilms and the production of virulence factors [3]. Currently, more than 70 species of bacteria with bacterial quorum-sensing abilities are known, including *Pseudomonas aeruginosa*, *Escherichia coli*, *Staphylococcus aureus,* and other pathogenic bacteria closely related to humans [4]. 

*Pseudomonas aeruginosa* (PA) is a widely distributed Gram-negative bacterium, ubiquitous in nature (soil, air, water, and other environments) [5]. An increasing number of healthcare-associated infections caused by multidrug (MDR) or extensively drug-resistant (XDR) *P. aeruginosa* strains, combined with their worldwide spread, is concerning due to limited therapeutic options [6]. The mechanism of PA resistance to antibiotics is very complex, including biofilm regulation via the quorum-sensing system of PA, which is also one of the important causes of resistance. QS regulates more than 300 genes of *P. aeruginosa*, and it is essential for both drug resistance and pathogenicity in *P. aeruginosa* [7].

**Figure 1:**
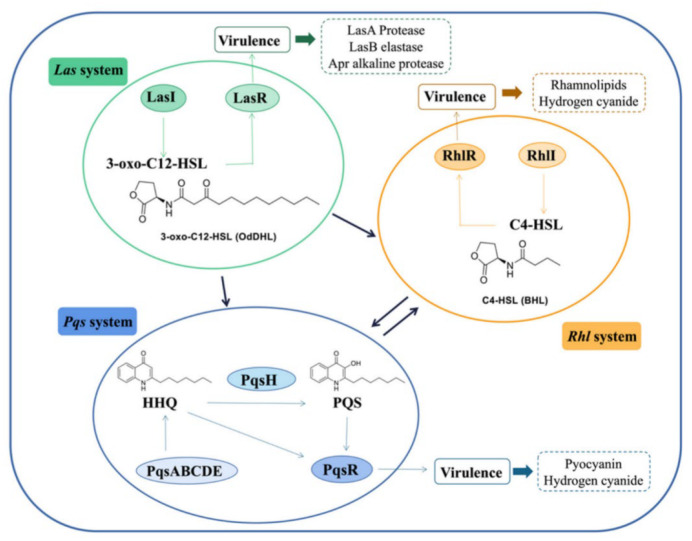
The figure shows the three main QS systems in *P. aeruginosa*—the *las* system, *rhl* system, and *pqs* system—and their signaling molecules. *Las* is indicated in green; *rhl* is indicated in orange; *pqs* is indicated in blue. *Las* is at the top of the QS hierarchy and influences *rhl* and *pqs.* On the other hand, *rhl* is under the control of both *las* and *pqs* [19].

*P. aeruginosa* employs three major interconnected QS systems that function independently and dependently involving the *las*, *rhl*, and *pqs* pathways as well as novel candidate quorum-sensing inhibitor (QSI) pathways regulated by several QS signal molecules (Figure 1). The *las* and *rhl* systems use *N*-acylhomoserine lactones (AHLs) as signal molecules [8]. AHLs are the best-characterized QS signal molecules. Different AHLs possess a homoserine lactone ring with an attached fatty acyl side chain of 4 to 20 carbons (Table 1) [9,10,11]. In the *las* system, *N*-3-oxododecanoyl-homoserine lactone (3-oxo-C12-HSL), which binds to the lasR receptor protein, is synthesized under the direction of lasI protein synthetase. A number of virulence factor genes, including *lasB*, *apr*, and *toxA*, are further expressed in the regulation of the *las* system [12]. In the *rhl* system, rhlI protein synthetase produces *N*-butyryl-homoserine lactone (C_4_-HSL), which can be bound by the rhlR receptor protein. The expression of genes involved in the synthesis of rhamnolipid, pyocyanin, and hydrogen cyanide synthase is regulated by the *rhl* system [13,14]. The third QS system is the *pqs* system, and it is interlinked with the other two systems. 2-Heptyl-3-hydroxy-4-quinolone (PQS) is a signal molecule that *P. aeruginosa* can produce through the *pqs* system. Studies indicate that the *pqs* system functions as a link between the *rhl* and *las* systems [15,16]. These three signaling systems are connected, mutually regulated, and work together to control the synthesis of virulence factors, the formation of biofilms, and bacterial movement. (Figure 1) [17]. As a result, the creation of homoserine analogs is thought to be a novel approach to creating QS modulators [18].

**Figure 2:**
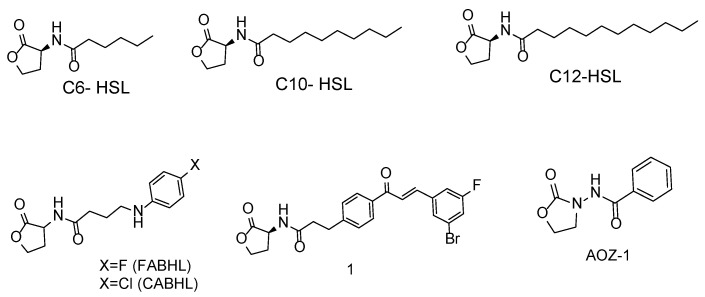
The figure shows the AHL classes of QSIs.

Structural modifications to AHL molecules are used to create QSIs. The length of the acyl side chain significantly affects the activity of natural AHLs, and lengthening the side chain can convert excitatory activity into inhibitory activity. *C. violaceum*’s autoinducer (AI) is a C_6_-HSL, and its receptor protein is CviR (Figure 2). A side chain of an HSL with 10 carbons (C_10_-HSL) has a significant inhibitory effect on CviR, and AhyR, CviR, and LuxR are inhibited by the small molecule 3-oxo-C_12_-HSL of LasR. (Figure 2) [20]. Some researchers have attempted to design inhibitors based on AHL signaling molecules. The structures of *N*-(4-{4-fluoroanilno} butanoyl)-L-homoserine lactone (FABHL) and *N*-(4-{4-chlororoanilno} butanoyl)-L-homoserine lactone (CABHL) are similar to HSLs (Figure 2). These two compounds can influence *las* via interaction with the LasR protein [21]. Wu et al. focused on central amide moieties and synthesized a series of chalcone-based L-homoserine lactones; among these compounds, (*S*)-2-((4-(3-(4bromo-2-fluorophenyl) acryloyl) phenyl) amino)-*N*-(2-oxotetrahydrofur-an-3-yl) acetamide 3 (Figure 2) was found to inhibit the LasR-dependent QS system of *P. aeruginosa* [22]. Despite the discovery of numerous extremely active analogs of AHLs, these molecules have a few notable drawbacks. First of all, in an alkaline environment, these compounds are prone to lose their activity due to ring opening. Second, other bacteria or host quorum quenching enzymes can easily break down these molecules [23]. Thus, in previous studies, LuxR-type QS transcriptional regulatory protein was employed as the target to obtain a better QSI. In our research group, Zhao [24] screened the compound AOZ-1 via computer-aided drug design. Its parent nucleus configuration is basically consistent with the configuration of homoserine lactone. Additionally, compound AOZ-1 is stable under alkaline conditions. Therefore, AOZ-1 was used as the lead compound in this study.

In this study, through the docking analysis of AOZ-1 and CviR protein, twenty-four novel 3-amino 2-oxazolidinone compounds were designed and synthesized. Furthermore, in order to further the inventiveness of compound AOZ-1, nine novel parent nucleus compounds were created and synthesized by substituting a six-membered ring structure for the parent nucleus of 3-amino-2-oxazolidinone. For the preliminary screening of the compounds, *C. violaceum* CV026 was used as a report strain together with 3-amino-tetrahydro-l,3-oxazin-2-one. A compound with a new nucleus core component of 3-amino-tetrahydro-l,3-oxazin-2-one was found to inhibit the QS of CV026. Next, in vitro and in vivo tests were used to assess the test chemicals’ inhibitory effect on *P. aeruginosa* PAO1 QS. Through in vitro experiments, the effects of the novel parent nuclear compound were assessed on the biofilm, virulence factors (pyocyanin, rhamnolipid, and elastase), and swarming motility. This study also examined the impact of the novel parent nuclear component on the ability of infected *C. elegans* to survive. This study also performed cytotoxicity experiments and in vivo pharmacokinetics experiments on new parent nucleus compounds. In general, this study identified a compound, **Y-31**, with a new nucleus core component of 3-amino-tetrahydro-l,3-oxazin-2-one, and it offers a fresh strategy for the treatment of clinical drug-resistant infections.

## 2. Results and Discussion

### 2.1. Chemistry

#### 2.1.1. Analysis of the Interaction between AOZ-1 and CviR Protein and the Design of Its Derivatives and Synthesis

**Figure 3:**
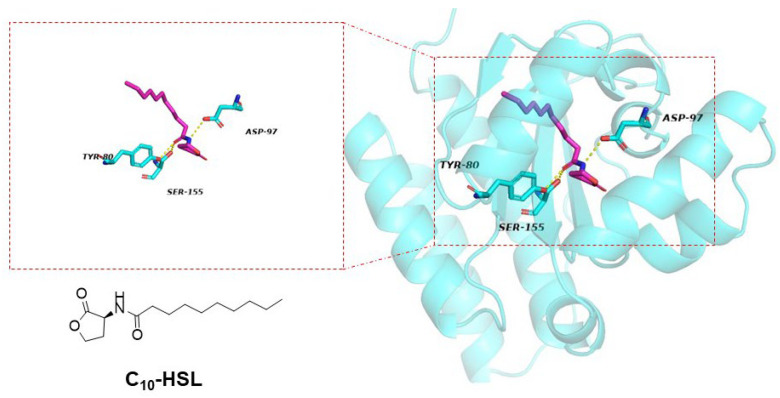
The figure shows a close-up of the CviR/C_10_-HSL crystal complex structure and key sites (Red is C_10_-HSL).

In order to study the regulation mechanisms of quorum sensing in *C. violaceum*, Chen et al. obtained the co-crystalline structure of the transcription factors CviR and C_10_-HSL [25] (Figure 3). C_10_-HSL is a good inhibitor of quorum sensing in *C. violaceum*. Co-crystal structure analysis showed that the cavity between the CviR protein and ligand had two parts: one was hydrophilic, and the other was narrow and hydrophobic. Tyrosine Tyr80, serine Ser155, and aspartic acid Asp97 could form hydrogen bonds with homoserine lactone and amide bonds.

In order to find new quorum-sensing inhibitors, Zhao [24] applied DOCK4.0 software to virtually screen the MDL database, the ACD database, and a self-built database. By observing the binding of the compounds to the receptor cavity and the formation of hydrogen bonds with the key amino acid residues of tyrosine Tyr80, tryptophan Trp84, serine Ser155, and aspartic acid Asp97, the 3-amino-2-oxazolidinone compound of AOZ-1 was selected as the lead compound from the database.

**Figure 4:**
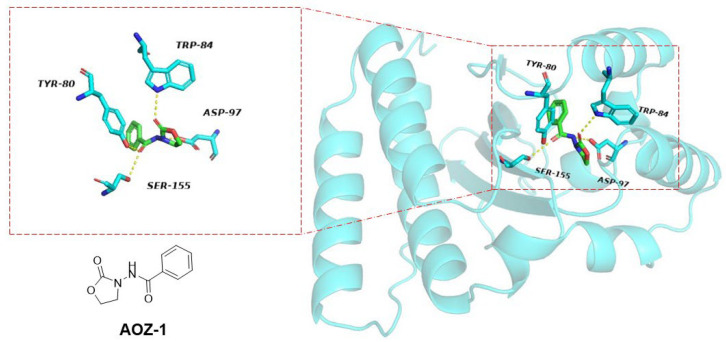
The figure shows the docking results and close-up of key sites between AOZ-1 and CviR molecules (Green is AOZ-1).

In this project, AOZ-1 was docked with CviR (PDB: 3QP5) (Figure 4). From the docking results, we can see that AOZ-1 can enter the cavity of the active site of CviR and has a good fit with CviR. Moreover, it can form hydrogen bonds with the key amino acid residues of the CviR active site, such as tryptophan Trp84, serine Ser155, and aspartic acid Asp97. Under alkaline conditions, AHL compounds easily open the ring and lose their activity, while 3-amino-2-oxazolidinones can maintain their stability under alkaline conditions. Zhao [24] measured the half-maximal inhibitory concentration (IC_50_) of AOZ-1 on *C. violaceum* CV026 QS in accordance with the suppression. The results showed that AZO-1 had some inhibitory activity, but the activity was weak (IC_50_ > 200). Therefore, we previously conducted preliminary modification studies on AOZ-1 [26]. In order to obtain compounds with better activity and novel structures, we further modified AOZ-1.

**Figure 5:**
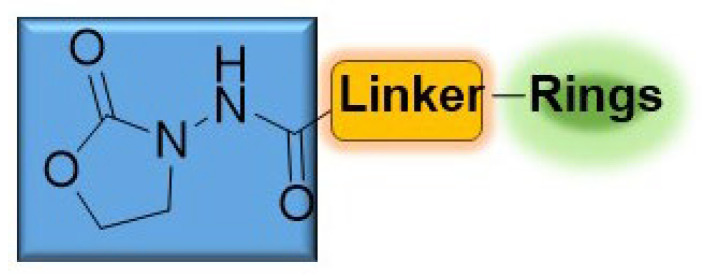
The figure shows the design strategy of the novel QSI.

In this study, AOZ-1 was taken as the lead compound, and combined with the previous work, the structure of AOZ-1 was modified by the principle of bioisosterism, with the expectation of obtaining a target compound with better compatibility with CviR. The specific structural transformation methods are as follows (Figure 5).

First, we modified the linker, which is mainly divided into the following. (1) Studies have shown that changing the length of a carbon chain will affect the activity of bacterial quorum sensing; therefore, we changed the length of the carbon chain to observe the effect of carbon chain length on the activity. (2) Although O and S have similar bond angles and spatial structures, they have different electronegativity and hydrophobicity; thus, the O atom was replaced with an S atom to observe the changes in biological activity.

Second, we transformed the region of the rings. The modifications were as follows. (1) The benzene ring was replaced with different aromatic heterocyclic groups because the electron isoefflux of the ring can modulate the activity and improve the pharmacokinetic properties. (2) Since the unsubstituted aromatic ring is easily oxidized in vivo, we substituted the benzene ring ends with different groups, firstly with different halogen atoms and, secondly, with different groups, such as trifluoromethyl.

**Scheme 1:**
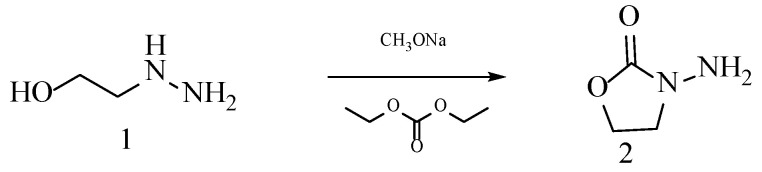
The scheme shows the synthetic route of the intermediate compounds.

**Scheme 2:**
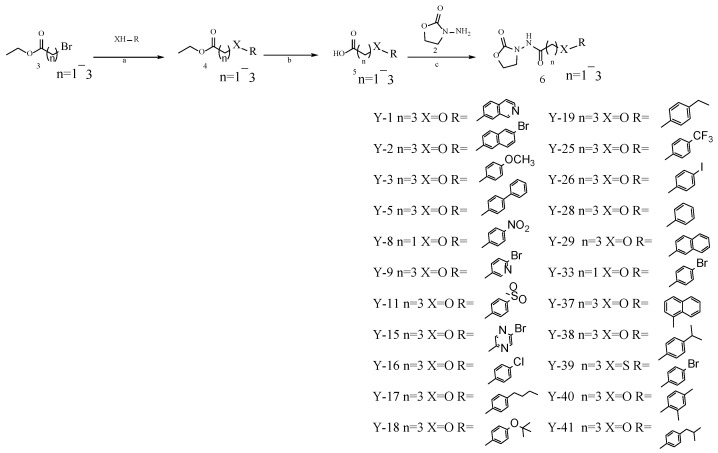
The scheme shows the synthetic route of the target compounds. (a: Cs_2_CO_3_, CH_3_CN; b: NaOH, 3M HCl; c: HOBT, EDCI, TEA, DCM).

Finally, according to the synthetic route, we obtained a series of novel oxazolidinone compounds (Scheme 1 and Scheme 2).

Target compound **2** was synthesized using 2-hydrazine ethanol (**1**) and diethyl carbonate as raw material via cyclization catalyzed using sodium methoxide (Scheme 1). Using a bromocarboxylic acid ester (**3**) and the equivalent phenol or thiophenol compound as raw materials, target compound 4 was produced using heat reflux, cesium carbonate catalysis, and a substitution reaction. (Scheme 2). Target compound **5** was hydrolyzed by heating the intermediate compound **5** under NaOH conditions (Scheme 2). Using the intermediate compound **5** and 3-amino-2-oxazolidinone (**2**) as raw materials, target compound **6** was created via the condensation of 1-(3-Dimethylaminopropyl)-3-ethylcarbodiimide hydrochloride (EDCI) and 1-hydroxybenzotriazole (HOBT), with TEA acting as a catalyst (Scheme 2).

#### 2.1.2. Analysis of Inhibition of *C. violaceum* QS Activity, Screening, and SAR Analysis of AOZ-1 Derivatives

In the exploration of the QS inhibitions, *C. violaceum* CV026, a mini-T5 mutant of *C. violaceum* ATCC31532, is frequently employed as a biosensor strain. Because *C. violaceum* CV026 lacks the CviI gene, which directs the synthesis of AHLs, it is unable to manufacture violacein on its own. The violacein reaction can be induced in *C. violaceum* CV026 by adding exogenous C_6_-HSL [27]. As a result, the violacein reaction in *C. violaceum* CV026 can be used to assess the compounds’ suppression of QS, as used in this investigation.

In this study, we first assessed the impact of several compounds on *C. violaceum* CV026’s QS inhibitory activities. According to our findings, *C. violaceum* CV026’s QS was inhibited by the chemicals **Y-1**, **Y-3**, **Y-9**, **Y-13**, **Y-15**, **Y-17-19**, **Y-25**, **Y-26**, **Y-28**, **Y-29**, and **Y-37-40**. In order to conduct more research, we employed C_10_-HSL as a positive control and measured the half-maximal inhibitory concentration (IC_50_) of the test compounds on *C. violaceum* CV026 QS in accordance with the suppression of violacein production. Compound **Y-15** (IC_50_ = 91.33 ± 3.02 µM), **Y-25** (IC_50_ = 62.62 ± 3.27 µM), **Y-26** (IC_50_ = 27.56 ± 1.25 µM), **Y-29** (IC_50_ = 26.14 ± 2.52 µM), and **Y-39** (IC_50_ = 47.44 ± 1.74 µM) had a better inhibitory effect on *C. violaceum* CV026 QS. (Table 1). 

Based on the following analysis, we developed a preliminary structure–activity relationship for the steroid-induced inhibition of *C. violaceum* CV026 by the 3-amino-2-oxazolidinone target compounds. By comparing the IC_50_ of compounds **Y-8**, **Y-16**, **Y-24**, **Y-26**, **Y-30**, **Y-33**, and **Y-39** against CV026, we can find that the inhibition activity is best when the length of the linker is three carbon atoms linked to one oxygen atom. By comparing the IC_50_ of compounds **Y-1**, **Y-9**, **Y-11**, **Y-18**, **Y-19**, **Y-25**, **Y-26**, **Y-29**, **Y-37**, and **Y-41** against CV026, it was found that the inhibition activity was best when the ring is benzene and the benzene ring is linked to halogen atoms. Based on the above conclusions, we found that compounds **Y-26** and **Y-29** had the best inhibitory activity.

#### 2.1.3. Design and Activity Screening of the New Parent Nucleus

**Figure 6:**
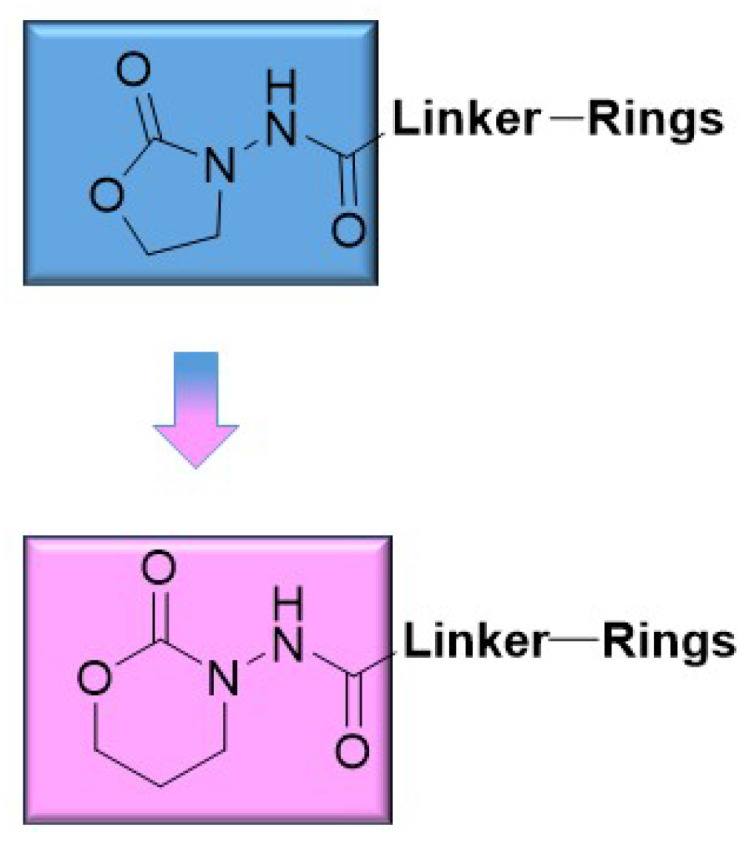
The figure shows the design strategies for novel inhibitors of novel parent nuclear structures.

Based on the structure of compound AOZ-1, we sought to find novel inhibitors with a new parent core structure. (Figure 6). By replacing the parent core of 3-amino-2-oxazolidinone with a six-membered ring structure, we designed and synthesized new parent compounds 3-amino-tetrahydro-l,3-oxazin-2-one compounds. The Linker and Rings of the new parent core compound were also preliminarily investigated in the same way as 3-amino-2-oxazolidinone.

**Scheme 3:**
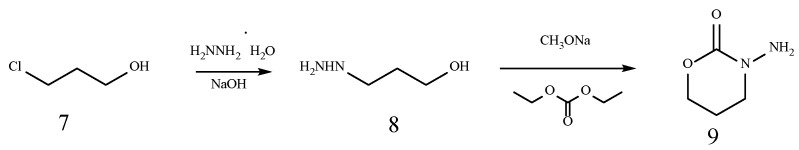
The scheme shows the synthetic route of the intermediate compounds.

**Scheme 4:**
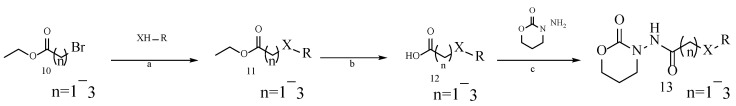

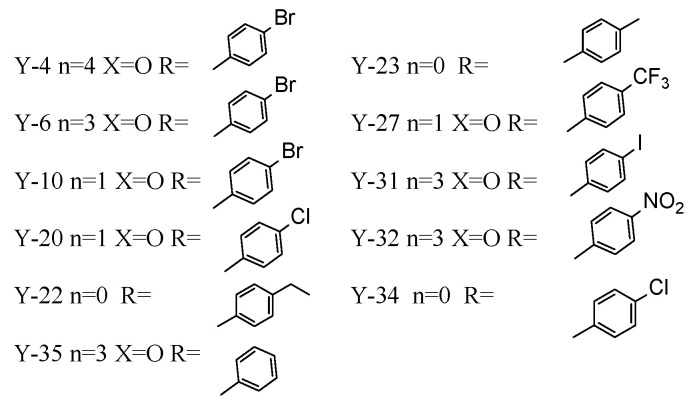
The scheme shows the synthetic route of the target compounds. (a: Cs_2_CO_3_, CH_3_CN; b: NaOH, 3M HCl; c: HOBT, EDCI, TEA, DCM).

Finally, according to the synthetic route, we obtained a series of novel 3-amino-tetrahydro-l,3-oxazin-2-one compounds (Scheme 3 and Scheme 4).

Target compound **8** was synthesized using 3-chloropropanol (**7**) and hydrazine hydrate as the raw materials. Target compound **9** was synthesized using 3-Hydrazino-1-propanol (**8**) and diethyl carbonate as the raw materials via cyclization catalyzed by sodium methoxide (Scheme 3). Using a bromocarboxylic acid ester (**10**) and the equivalent phenol or thiophenol compound as raw materials, target compound **11** was produced using heat reflux, cesium carbonate catalysis, and a substitution reaction (Scheme 4). Target compound **12** was hydrolyzed by heating intermediate compound 5 under NaOH conditions (Scheme 4). Using intermediate compound **12** and the 3-amino-tetrahydro-l,3-oxazin-2-one (9) as raw materials, target compound **13** was created via the condensation of 1-(3-Dimethylaminopropyl)-3-ethylcarbodiimide hydrochloride (EDCI) and 1-hydroxybenzotriazole (HOBT), with TEA acting as a catalyst (Scheme 4).

The activity of the compound with the novel parent core structure was tested in the same way as that used for 3-amino-2-oxazolidinone. The results of the activities obtained are shown in Table 2. Among the compounds with new parent nuclei, compound **Y-31** (IC_50_ = 91.55 ± 3.35 µM) showed the best inhibitory activity.

When studying the activity of the new parent compound, it was found that the new parent compound 3-amino-tetrahydro-l,3-oxazin-2-one compound **Y-31** (IC_50_ = 91.55 ± 3.35 µM) had good inhibitory activity, and the activity was better than that of **AOZ-1** (IC_50_ > 200). In addition, it was found that when the length of the linker was three carbon atoms linked to one oxygen atom, and when the Ring position of the benzene ring and the benzene ring para link was a halogen atom, the inhibition activity was the best. This is consistent with the SAR studies of 3-amino-tetrahydro-l,3-oxazin-2-one compounds. It further proved that when the length of the linker is three carbon atoms linked to one oxygen atom, and when the Rings is a benzene ring, and the benzene ring is linked to halogen atoms, the QS inhibition effect is better.

### 2.2. The Effect of the Compounds on P. aeruginosa Biofilm Formation

*P. aeruginosa* is a significant opportunistic infection found in hospitals. Therefore, it is crucial to the context of antibiotic resistance. Biofilm cells significantly increase the antibiotic resistance of bacteria when compared to planktonic cells. Numerous investigations have demonstrated that the QS system controls the development of bacterial biofilms.

**Figure 7:**
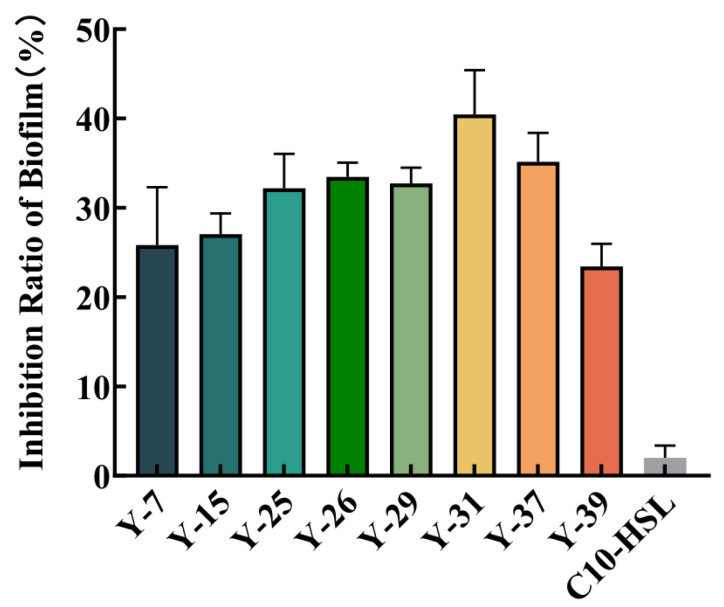
The figure shows the effect of the compounds on *P. aeruginosa* biofilm formation.

Compounds **Y-15**, **Y-25**, **Y-26**, **Y-29**, **Y-31**, **Y-37**, and **Y-39** were obtained from the preliminary activity test of *C. violaceum* CV026. Additionally, the crystal violet assay was used to ascertain the impact of the compounds on *P. aeruginosa* PAO1 biofilms (Table S1). As shown in Figure 7, compound **Y-31** significantly reduced the biofilm formation of *P. aeruginosa* PAO1. When the concentration of **Y-31** was 40 μM, the *P. aeruginosa* PAO1 biofilm was inhibited at a rate of 40.44%. C_10_-HSL had no significant inhibitory effect on the biofilm of *P. aeruginosa* PAO1, probably due to the different effects of the compounds on different bacterial genera. In addition, the inhibitory effects of compound **Y-26** and compound **Y-29** against *C. violaceum* CV026 were stronger than those of compound **Y-31**. In contrast, compound **Y-31** was more effective than compound **Y-26** and compound **Y-29** in inhibiting *P. aeruginosa* PAO1 biofilm formation at the concentration of 40 μM. This study demonstrated that the novel parent nuclear compound **Y-31** could decrease *P. aeruginosa* PAO1 biofilm formation, which would decrease *P. aeruginosa* PAO1’s resistance to therapeutic drugs. Therefore, this study aimed to conduct a more in-depth study on the inhibitory effect of **Y-31** on *P. aeruginosa* PAO1.

### 2.3. The Analysis of Growth Activity of ***Y-31*** against PAO1

**Figure 8:**
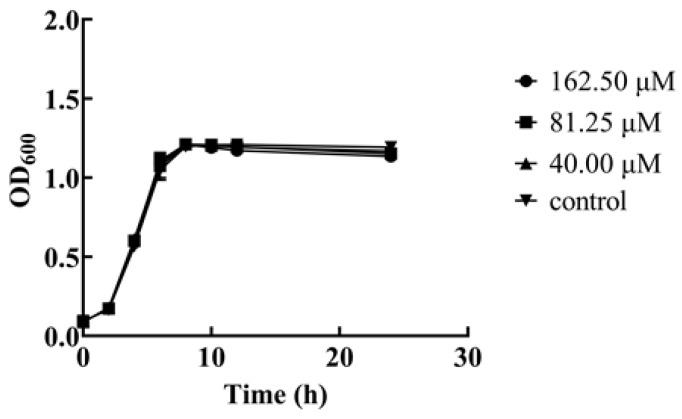
The figure shows the growth inhibition curve of **Y-31** on PAO1 (OD_600_: Absorbance value at 600 nm).

The difference between QSI and traditional clinical antibiotics is that QSI can inhibit the QS system of pathogenic bacteria without affecting the growth activity of bacteria, thereby reducing the pathogenicity and resistance of pathogenic bacteria and treating bacterial infections. It is precisely because QSI has no inhibitory effect on bacterial growth activity that the bacteria have no survival pressure and fewer bacterial resistance mutations [28]. Therefore, we evaluated the effect of the compound **Y-31** on the growth activity of PAO1 bacteria using C_10_ HSL as a positive control [25]. The results are shown in Figure 8. The compound **Y-31** had no significant inhibition on the growth activity of PAO1.

### 2.4. The Effect of ***Y-31*** on P. aeruginosa Virulence Factors

The pathogenicity of *P. aeruginosa* is largely dependent on the virulence factors that QS regulates. *P. aeruginosa* produces a blue-green phenazine pigment called pyocyanin, which is one of the causes of lung infections and has a significant impact on cystic fibrosis [29,30]. In *P. aeruginosa*, the rhl and pqs QS pathways regulate the synthesis of pyocyanin. [15]. Rhamnolipid is a prerequisite for the development of cystic fibrosis, which can kill polymorphonuclear (PMN) leukocytes in the host [31]. Rhamnolipid synthase, rhlAB, is responsible for the synthesis of rhamnolipid and is regulated by rhlR [32]. One of *P. aeruginosa*’s main virulence factors is called elastase, and it destroys the cells of the host. The lasB gene controls elastase. Due to the phenotypic characteristics of *P. aeruginosa*’s pathogenicity, the effects of compounds on QS can be evaluated by examining their inhibitory effects on the virulence factors of *Pseudomonas aeruginosa*.

**Figure 9:**
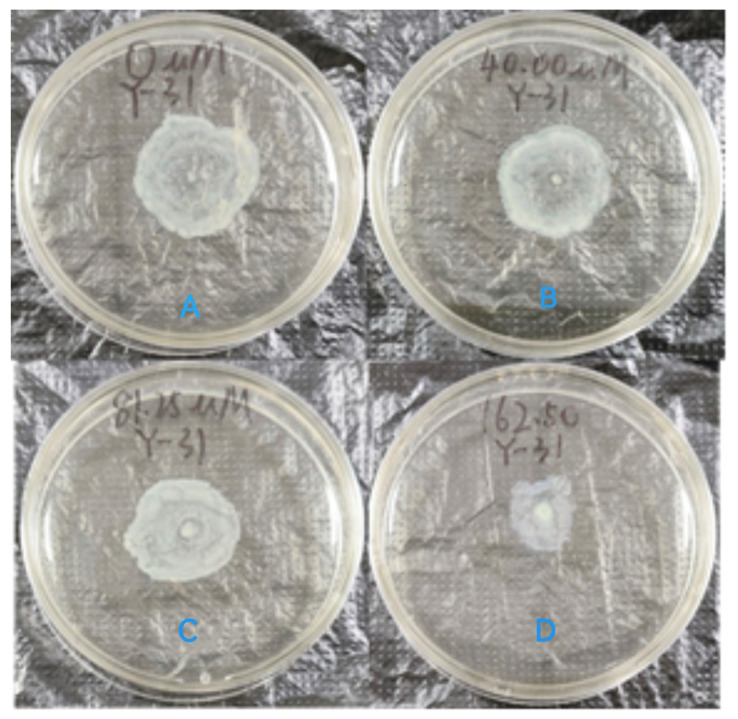
The figure shows the **Y-31** inhibition of *P. aeruginosa* PAO1’s swarming motility. Swarming *P. aeruginosa* PAO1 on 0.5% agar plates with **Y-31**. (**A**) Blank control, (**B**) 40 μM, (**C**) 81.25 μM, and (**D**) 162.5 μM is shown.

Therefore, the virulence factors (pyocyanin, rhamnolipid, and elastase) of *P. aeruginosa* PAO1 were examined to evaluate the effects of compound **Y-31** with a novel parent nucleus structure on the QS of *P. aeruginosa*. It can be observed from Table 3 that compound **Y-31** has a significant inhibitory effect on the virulence factors of PAO1 (pyocyanin, rhamnolipid, and elastase), and this inhibitory effect is related to its concentration. When the concentration of **Y-31** was 162.5 μM, the inhibition rates of pyocyanin, rhamnolipid, and elastase were 22.5%, 6.1%, and 22.7%, respectively. Therefore, the results indicate that compound **Y-31** with a novel parent nucleus structure has some inhibitory effect on the QS of PAO1. Furthermore, the movement of bacteria on swarming agar plates was used to assess the impact of compound **Y-31** (40 μM, 81.25 μM, and 162.5 μM) on *P. aeruginosa* PAO1’s swarming motility. According to the results, compound **Y-31** significantly reduced swarming motility as compared to the control group. (Figure 9). In summary, compound **Y-31** with a new parent nucleus structure inhibited *P. aeruginosa* PAO1 virulence factors in a concentration-dependent manner; this effect may have been caused by the blocking of *P. aeruginosa* PAO1’s QS pathways. 

### 2.5. The Effect of ***Y-31*** on the Survival of Infected C. elegans

**Figure 10:**
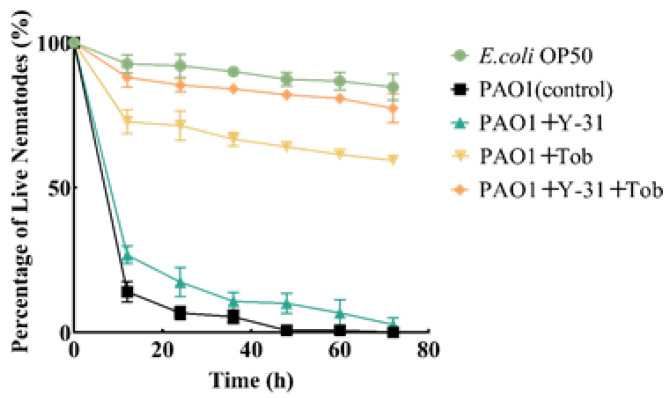
The figure shows the effect of **Y-31** (162.5 μM), the antibiotic Tob (0.25 μg/mL) alone, and a combination of both drugs on the survival of *C. elegans* infected with *P. aeruginosa* PAO1 (values are presented as mean ± SD, *n* = 3).

Using wildtype Caenorhabditis elegans N2 as a model, the compound’s inhibitory effect on *P. aeruginosa* QS in vivo was further investigated. Many benefits come with *C. elegans*, including its compact size, quick generation time, and ease of culture under lab conditions [33]. Research has demonstrated a connection between *C. elegans*’ mortality and *P. aeruginosa*’s QS-mediated virulence components [34]. *P. aeruginosa* grows in *C. elegans*’ stomach and produces virulence proteins like pyocyanin, which kill nematodes [35]. Three groups were used to determine the survival rate of nematodes infected with *P. aeruginosa* PAO1: compound **Y-31** alone, Tobramycin (Tob) alone, or compound **Y-31** and Tob. It was discovered that compound **Y-31** (162.5 μM) alone or Tob (0.25 μg/mL) alone significantly reduced the pathogenicity of PAO1 and improved the survival rates of infected nematodes by an average of 9.33% and 63.33% after 48 h, respectively. Moreover, compared with **Y-31** or Tob alone, the results from the **Y-31** combined with the Tob group significantly improved the average survival rate of nematodes (Figure 10). As a result, the compound **Y-31** prolonged the life of nematodes infected with PAO1, and when combined with Tob, it significantly improved nematode survival. As a QSI, the compound **Y-31** prolongs the lifespan of infected nematodes by inhibiting the QS of *P. aeruginosa*.

### 2.6. The Cytotoxicity Test

**Figure 11:**
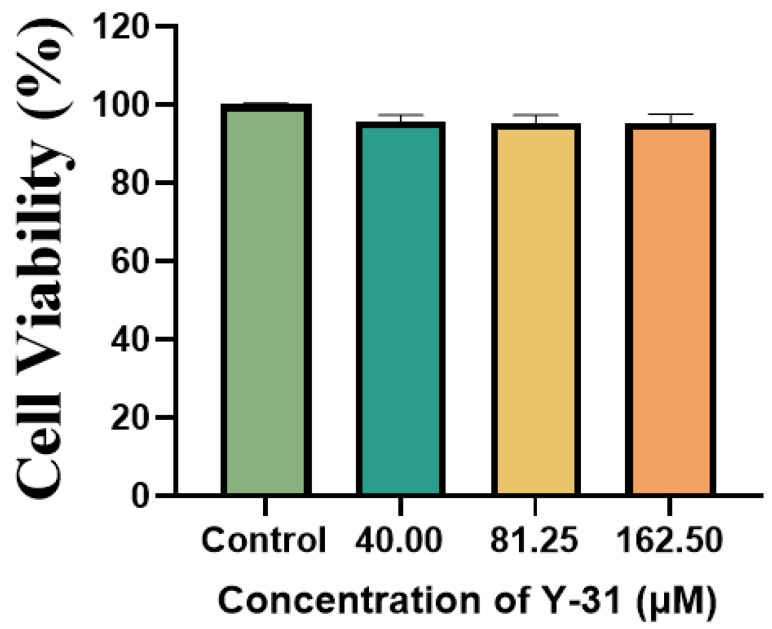
The figure shows the effects of compound **Y-31** on the proliferation of RAW264.7 cells.

Cytotoxicity tests are commonly used to assess toxicity and biological activity, making them one of the most crucial experimental approaches when evaluating the toxic effects of chemicals on cells. In comparison to in vivo experiments, cytotoxicity tests offer advantages in terms of speed, simplicity, high repeatability, low cost, and high predictability. These tests are often utilized as key tools for rapidly screening the toxicity of chemicals. In this study, the compound **Y-31** (162.50 μM, 81.25 μM, and 40.00 μM) was incubated with RAW 264.7 cells for 12 h, and the impact of compound **Y-31** on cell viability was assessed using the CCK8 method. The results are presented in Figure 11. At concentrations of 162.50 μM, 81.25 μM, and 40.00 μM, the compound **Y-31** neither inhibited nor promoted the viability of RAW 264.7 cells, indicating that the compound **Y-31** did not exhibit significant cytotoxicity towards RAW 264.7 cells.

### 2.7. ADMET

In order to assess the in vivo pharmacokinetics of **Y-31**, we chose a rodent pharmacokinetic model to evaluate ADME properties (Table 4). When dosed in mice, **Y-31** could be absorbed with a long terminal half-life (T_1/2_ = 5.18 h). This indicates that **Y-31** can inhibit biofilm and virulence factor production in vivo for a longer period of time. The short time to reach the peak concentration indicated that compound (T_max_ = 0.25 h) **Y-31** exerted its efficacy more quickly. Compound **Y-31** reached a high peak concentration in vivo (C_max_ = 1279 ng/mL) and exhibited high bioavailability (F = 30.4%), indicating that it could exert efficacy in vivo following oral administration. Therefore, the compound **Y-31** is valuable for further research.

## 3. Materials and Methods

### 3.1. General Chemistry

The ^1^H-NMR test instrument used in this study was a JNM-ECA-400 superconducting NMR instrument (Agilent company, Santa Clara, CA, USA); the internal standard was TMS. The MS was determined by an API-150 ex (ESI) mass spectrometer (Agilent company, Santa Clara, CA, USA). Thin-layer chromatography silica gel was purchased from Qingdao Ocean Chemical GF254 silica gel (Qingdao Ocean Chemical Co., Ltd., Qingdao, China); 7-Hydroxyisoquinoline, 4-Phenylphenol, 4-Methoxyphenol, 4-Chlorophenol, and other significant reagents were purchased from Alfa (Holbrook, NY, USA), and the remaining reagents were utilized without additional purification after being bought commercially.

The specific steps of compound synthesis are described in the Supplementary Materials.

### 3.2. Biological Assays

#### 3.2.1. Medium, Bacterial Strains, and Experimental Drugs

*C. violaceum* CV026, *E. coli* OP50, and *P. aeruginosa* PAO1, representing wildtype strains, were stored in our laboratory. Luria–Bertani (LB) broth (1% tryptone, 1% NaCl, 0.5% yeast extract) was typically used to cultivate the bacteria, in which *C. violaceum* CV026 was cultivated at 30 °C; *P. aeruginosa PAO1* and *E. coli* OP50 were cultured at 37 °C. Dimethyl sulfoxide (DMSO) was used to dissolve the test compound to a stock concentration of 65 mM. The tobramycin base was purchased from Sigma (St. Louis, MO, USA), and it was stored at 6.4 mg/mL.

#### 3.2.2. The Half-Maximal Inhibitory Concentration (IC_50_) of Compounds against *C. violaceum* CV026 QS [36]

Initially, the compound that demonstrated QS inhibitory activity was sequentially diluted 2 times with DMSO, resulting in concentrations of 650 mM, 325 mM, 162.5 mM, 81.25 mM, 40 mM, 20 mM, 10 mM, 5 mM, and 2.5 mM. Subsequently, the log phase of *C. violaceum* CV026 was placed in a 12-well plate (2 mL/well) after being diluted 10-fold with fresh LB medium. The experimental group received 15 μL of the diluted inducer C_6_-HSL (0.125 mM), and 8 μL of each dilution gradient compound was added to each well, while the positive control group received 8 μL of C10-HSL, and the blank control group received 8 μL of LB medium. The plates were incubated at 30 °C and 130 rpm for 12 h. After centrifuging 1 mL of the culture for 10 min at 11,255 g, the supernatant was discarded. After dissolving the violacein with 500 μL of DMSO, the mixture was centrifuged once more at 11,255 g for 10 min. In order to measure the absorbance at 585 nm, 200 μL of the upper pigment was added to a 96-well plate. The IC_50_ was calculated using GraphPad Prism 5 according to the formula (absorbance at 585 nm of the test concentration/the absorbance of the control at 585 nm × 100%).

#### 3.2.3. Biofilm Quantification Assays

The crystal violet assay was used to quantitatively analyze the biofilm, as previously described [37]. *P. aeruginosa* PAO1 log phase was diluted 100 times with fresh LB medium and incubated for 3.5 h at 37 °C and 150 rpm. After that, the *P. aeruginosa* PAO1 bacterial culture was added to the 96-well plate. *P. aeruginosa* PAO1 bacterial culture in 90 μL was added to the experimental group, and 10 μL of the test compound at a final concentration of 325 μM, 162.5 μM, 81.25 μM, 40 μM, 20 μM, and 10 μM. Each well received 100 μL of bacterial culture for the control group and 100 μL of fresh LB medium for the blank group. After 20 h of incubation at 37 °C, the bacterial culture was removed from the 96-well plate, and it was dried after three PBS washes. Each well received 150 mL of 0.1% crystal violet (CV) for a 15 min staining period. Each well was washed three times with PBS after the CV was removed. Lastly, each well received 150 μL of 33% glacial acetic acid, and a Biotek multi-function microplate reader was used to measure the absorbance value at 595 nm. The biofilm inhibition rate was calculated as follows = (OD_595 blank_ − OD_595 sample_)/OD_595 blank_ × 100%.

#### 3.2.4. The Growth of the Compound of PAO1 QS Activity Analysis

*P. aeruginosa* PAO1 in log phase was diluted to OD_600_ = 0.05 in fresh LB medium. Then, 98 μL of diluted bacterial solution and 2 μL of **Y-31** solution were added to the 96-well plate. The final concentration of **Y-31** in each well was 162.50 μM, 81.25 μM, and 40.00 μM, respectively. The pores without the compound **Y-31** were used as blank controls. The cells were incubated in an incubator at 37 °C for 24 h, and OD_600_ was measured at 0 h, 2 h, 4 h, 6 h, 8 h, 10 h, 12 h, and 24 h, respectively.

#### 3.2.5. Virulence Factor Quantification Analysis

The method used to perform the pyocyanin quantification assay was previously reported [38]. In short, *P. aeruginosa* PAO1 was cultivated in LB medium to reach the logarithmic growth phase at 37 °C and 150 rpm. Following this, the medium (2% peptone, 1% K_2_SO_4_, 0.3% MgCl_2_) was diluted 10 times. A 5 mL bacterial culture containing and excluding the compound (162.5 μM, 81.25 μM, and 20 μM) was incubated for 16 h at 37 °C at 150 rpm. The culture was centrifuged at 1665 g for 10 min in order to extract pyocyanin. The supernatant was then collected and extracted using 3 mL of chloroform. The upper aqueous phase was then collected via centrifugation at 1665 g for 10 min after 1 mL of 0.2 M HCl was combined with the chloroform layer. Absorbance was measured at 520 nm. The pyocyanin inhibition rate was calculated as follows = (OD_520 blank_ − OD_520 sample_)/OD_520 blank_ × 100%.

The rhamnolipid quantification assay was as follows: sulfuric acid–orcinol concentrated solution was used to examine the compound’s impact on *P. aeruginosa* PAO1 rhamnolipid. Initially, 50 μg/mL, 100 μg/mL, 200 μg/mL, and 400 μg/mL of a prepared solution of rhamnose standard at a concentration of 1 mg/mL in tilled water were diluted. Subsequently, 900 μL of 0.19% orcinol-concentrated sulfuric acid solution (0.19% orcinol (w/v), 50% H_2_SO_4_) was mixed with 100 μL of each concentration of rhamnose standard solution, and the mixture was incubated for 30 min at 80 °C in a water bath. After cooling to 25 °C, the OD value at 421 nm was measured. A standard curve of rhamnose concentration–absorbance values was established using Excel software 2019.

After cultivating *P. aeruginosa* PAO1 in LB medium for a logarithmic growth phase at 37 °C and 150 rpm, the bacterial culture was diluted with PPGAS solution (20 mM KCl, 20 mM NH_4_Cl, 0.5% glucose, 1.6 mM MgSO_4_, 120 mM Tris-HCl, and 1.0% peptone) to OD_600_ = 0.05. After adding the compound to final concentrations of 162.5 μM, 81.25 μM, and 20 μM, it was cultivated for 48 h at 37 °C and 150 rpm. After centrifuging the bacterial cultures for 10 min at 1665 g, the supernatants were gathered. After extracting the supernatants twice using ethyl acetate, the organic layers were gathered and allowed to dry out at room temperature overnight. After resuspending the solid product in deionized water, 100 μL was added to 900 μL of 0.19% orcinol in 50% H_2_SO_4,_ and the mixture was incubated for 30 min at 80 °C in a water bath. After cooling to 25 °C, the OD value at 421 nm was measured. The rhamnolipid content in the sample was determined according to a rhamnose standard curve (rhamnolipid content = 2.5 × rhamnose content).

The method previously described was used to perform the elastase quantification experiment [39]. In LB medium, *P. aeruginosa* PAO1 was cultivated until the logarithmic growth phase at 37 °C and 150 rpm. The compound was added to final concentrations of 162.5 μM, 81.25 μM, and 20 μM and cultured at 37 °C at 150 rpm for 24 h. Elastase activity was detected using the EnzCheckTM Elastase Assay Kit after filtration with a 0.22 μm filter membrane.

#### 3.2.6. Swarming Motility Analysis

After being inoculated into LB medium, *P. aeruginosa* PAO1 was cultivated at 37 °C and 150 rpm until OD_600_ = 1.0. Then, 1 μL of *P. aeruginosa* PAO1 bacterial medium was inoculated into the center of swarming solid medium (0.8% nutrient broth (BD), 0.5% glucose, 0.3% agar (BD)) and cultured at 30 °C for 16 h. Then, the colonies of the swarming plate were picked and added to the center of the cooled swarming solid medium (0.8% nutrient broth (BD), 0.5% glucose, 0.5% agar (BD)), which had been supplemented with compounds and cultured at 30 °C for 24 h [40].

#### 3.2.7. *C. elegans* Survival Assay

In TSB medium (1.5% tryptone, 0.5% soy peptone, 0.5% NaCl, pH = 7.2 + 0.2), *P. aeruginosa* PAO1 was cultivated overnight at 37 °C. After uniformly spreading it on PGS agar plates (1% peptone, 0.15 M sorbitol, 1% NaCl, 1% glucose, and 1.7% agar) with and without the test compound, a 20 μL broth culture of *P. aeruginosa* was cultivated for 18 h at 37 °C to create a bacterial lawn. The negative control was the PGS agar plate coated with *E. Coli* OP50. Next, 50 wildtype *C. elegans* N2 strain-synchronized nematodes (L4 stage) were chosen and placed onto the plates. Using a stereomicroscope, the plates were counted every 12 h while being incubated at 20 °C. When the nematodes in the study were immobile, they were deemed dead. The number of nematodes that survived was tabulated to generate a survival curve [41].

#### 3.2.8. Cytotoxicity Test

1. Cell Culture

The cell line used in this study was mouse mononuclear macrophage leukemia cell RAW 264.7. The cells were cultured in DMEM complete medium (basal medium containing 10% fetal bovine serum, 100 U/mL penicillin, and 100 mg/mL streptomycin) at 37 °C in a 5% CO_2_ incubator. When the cells covered 80% of the bottom of the plate, they were passaged more than three times.

2. CCK8 Detection of Cell Proliferation

Adherent RAW 264.7 cells were digested to detach the cells. Digestion was stopped via the addition of PBS and centrifuging at 1000 rpm for 5 min. The supernatant was discarded, and the cells were resuspended in 1 mL of medium for cell counting. Based on the counting results, the cell suspension was diluted. Then, 100 μL of the diluted cell suspension was added to a 96-well plate, with approximately 1 × 104 cells per well. The cells were then placed in the incubator to allow for adherence.

One well without cells received 100 μL of complete medium as a blank control, and one well with cells received 100 μL of complete medium as a negative control. Simultaneously, a concentration gradient of the compound was prepared, and 100 μL of the compound and complete medium mixture were added to the wells. The compounds were incubated for 12 h at final concentrations of 162.50 μM, 81.25 μM, and 40.00 μM.

After incubation, the medium was removed, and 100 μL of 10% CCK8 solution was added to each well. The absorbance at 450 nm (OD_450_) was measured at 0.5 h, 1 h, and 2 h until OD_450_ = 1.

Cell viability was calculated using the following formula: cell activity = (OD_450_ compound − OD_450_ blank)/(OD_450_ negative control − OD_450_ blank) x 100%

#### 3.2.9. Pharmacokinetics

Beijing Pharmaron Inc. carried out pharmacokinetic studies relating to compound **Y-31** in mice. All of the animals were kept in a pathogen-free barrier animal facility, and the company’s Animal Care and Use Committee approved the experiments. On the day of administration, the sample solution was made into a 5, 20 mg/kg solution. Following a single dosage of compound **Y-31** either intravenously (5 mg/kg, IV) or orally (20 mg/kg, PO) at 0.25, 0.5, 1, 2, 4, 6, 8, and 24 h post-administration, blood samples were taken from CD1 male mice (*n* = 3). After that, the plasma was centrifuged for five seconds at 4000 g and 4 °C to yield. The plasma was diluted 3 times with water. For quantitative analysis, a measurement of 2 µL of diluted supernatant was put into the LC/MS/MS apparatus. Throughout the entire trial, no unusual clinical symptoms were noticed. Phoenix WinNonlin 8.0 software was applied to calculate the pharmacokinetic parameters.

## 4. Conclusions

In order to develop better biofilm bacterial inhibitors of PA, we carried out this study in two parts. First, based on the three-dimensional structure of transcription regulatory protein CviR and the characteristics of small-molecule quorum-sensing inhibitors, we took AOZ-1 as the lead compound with a better effect in terms of quorum-sensing inhibition, used the principle of bioelectron isoplatoon, and combined the previous work undertaken by our research group. A series of 3-amino-2-oxazolidinone target compounds were designed via the structural modification of the lead compound. SAR analysis was also carried out. It was discovered that when the length of the Linker is three carbon atoms linked to one oxygen atom, and when Ring is a benzene ring and the benzene ring is linked to halogen atoms, the QS inhibition effect was better. Compound **Y-26** satisfies all SAR conditions and thus has better inhibitory activity. Second, based on the structure of compound AOZ-1, we sought to find novel inhibitors with new parent core structures. By replacing the parent core of 3-amino-2-oxazolidinone with a six-membered ring structure, we designed and synthesized new parent 3-amino-tetrahydro-l,3-oxazin-2-one compounds. It was found that the new parent structure compound **Y-31** had a specific inhibitory effect on the QS of CV026, and it was stronger than AOZ-1. The results of SAR relationship analysis for its Linker and Rings were also consistent with the SAR of AOZ-1.

Because biofilms are one of the important causes of PA resistance, the inhibitory effects of compounds **Y-15**, **Y-25**, **Y-26**, **Y-29**, **Y-31**, **Y-37**, and **Y-39** on the biofilm of *Pseudomonas aeruginosa* PAO1 were tested. From the test results, it was found that compound **Y-31**, with a new parent nucleus structure, had the highest biofilm inhibition rate. Additionally, the compound **Y-31** had no inhibitory effect on bacterial growth activity; therefore, the bacteria had no survival pressure and fewer bacterial resistance mutations. Thus, the new parent compound **Y-31** was further subjected to a selectivity study for virulence factor and swarming determination in *P. aeruginosa* PAO1 in vitro. The new parent nucleus compound **Y-31** significantly inhibited the virulence factors and swarming of *P. aeruginosa* PAO1, and this inhibition is concentration-dependent. This study employed wildtype *C. elegans* N2 as an in vivo model to further investigate the compound’s inhibitory effect on *P. aeruginosa* QS. The new parent nucleus compound **Y-31** could prolong the lifespan of infected nematodes and, when combined with Tob trihydrate, can greatly enhance the survival of nematodes.

We also performed cytotoxicity experiments and in vivo pharmacokinetics experiments on new parent nucleus compounds of **Y-31**. The experimental results showed that the new parent nucleus **Y-31** is a compound with no toxic side effects, a fast onset time, a long retention time, and high bioavailability. 

In conclusion, we designed and synthesized a series of new oxazolidinones and 3-amino-1, 3-oxazinan-2-ketones. Compound **Y-31**, with a new parent nucleus structure, was found to have a certain inhibitory effect on *Pseudomonas aeruginosa* PAO1. Additionally, it is stable under alkaline conditions.

## Figures and Tables

**Table 1:** The table shows the anti-CV026 QS activities of 3-amino-2-oxazolidinone compounds.

Number	Structure	IC_50_ (µM)
**Y-1**	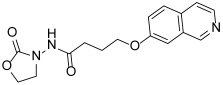	198.93 ± 13.88
**Y-2**	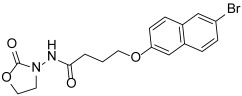	NA
**Y-3**	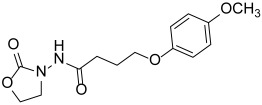	208.33 ± 7.26
**Y-5**	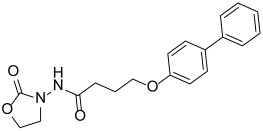	125.17 ± 16.05
**Y-8**	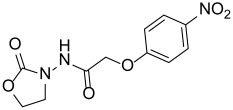	NA
**Y-9**	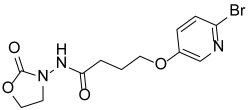	167.77 ± 7.28
**Y-11**	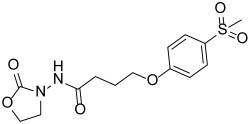	NA
**Y-15**	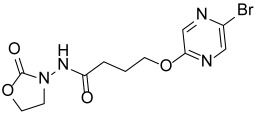	91.33 ± 3.02
**Y-16**	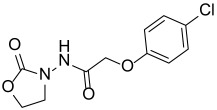	NA
**Y-17**	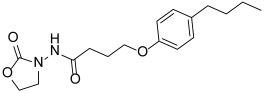	>260
**Y-18**	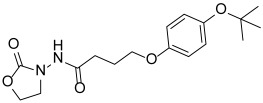	>260
**Y-19**	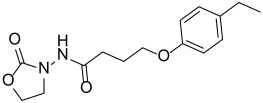	152.6 ± 8.78
**Y-24**	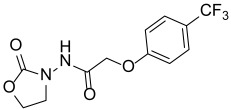	NA
**Y-25**	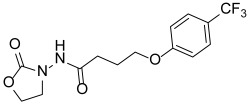	62.62 ± 3.27
**Y-26**	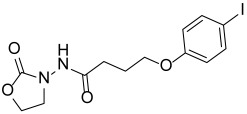	27.56 ± 1.25
**Y-28**	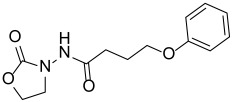	231.73 ± 5.84
**Y-29**	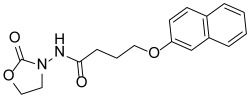	26.14 ± 2.52
**Y-33**	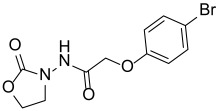	NA
**Y-37**	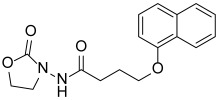	102.08 ± 4.36
**Y-38**	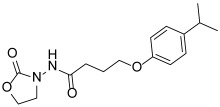	>260
**Y-39**	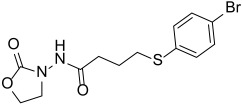	47.44 ± 1.74
**Y-40**	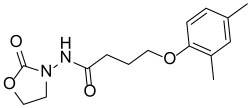	180.5 ± 1.77
**Y-41**	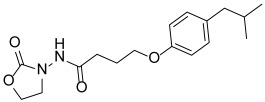	NA
**C_10_-HSL**	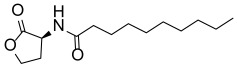	0.15 ± 0.024

Note: The results are reported as mean ± standard deviation (SD) of three independent tests. NA: not analyzed. IC_50_: the half-maximal inhibitory concentration.

**Table 2:** The table shows the anti-CV026 QS activities of 3-amino-tetrahydro-l,3-oxazin-2-one compounds.

Number	Structure	IC_50_ (µM)
**Y-4**	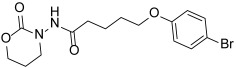	>260
**Y-6**	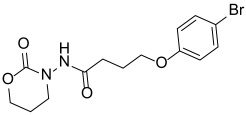	>260
**Y-10**	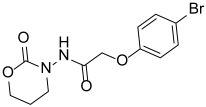	NA
**Y-20**	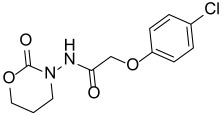	NA
**Y-22**	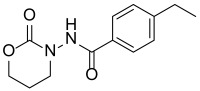	NA
**Y-31**	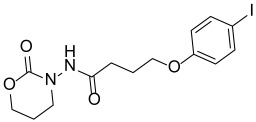	91.55 ± 3.35
**Y-32**	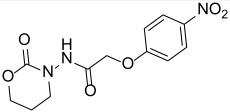	NA
**Y-34**	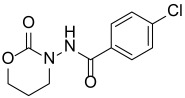	NA
**Y-35**	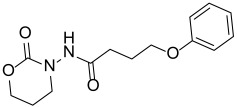	NA

Note: Results are reported as mean ± standard deviation (SD) of three independent tests. NA = not analyzed. IC_50_ = the half-maximal inhibitory concentration.

**Table 3:** The table shows the inhibition rates of PAO1 virulence factors by compound **Y-31** at different concentrations.

	Concentration	0 (μM)	40 (μM)	81.5 (μM)	162.5 (μM)
Virulence Factors					
Pyocyanin		0%	10%	17.1%	22.5%
Rhamnolipid		0%	0.4%	5.8%	6.1%
Elastase		0%	11.3%	20.0%	26.7%

Notes: (Inhibition rate = (OD_blank_ − OD_sample_)/OD_blank_ × 100%).

**Table 4:** The table shows compound **Y-31** IV and PO pharmacokinetic profiles in male CD1 mice.

Route	Dose (mg/kg)	AUC (h × ng/mL)	T_1/2_ (h)	T_max_ (h)	C_max_ (ng/mL)	F (%)
IV	5	935	0.13	0.08	3290	NA
PO	20	1100	5.18	0.25	1279	30.4

Notes: (IV: intravenous injection; PO: peros; AUC: area under curve; T_1/2_: half-life; T_max_: the time to reach the peak concentration; C_max_: the peak concentration of a drug in the body; F: bioavailability).

## Data Availability

All data are contained within the article or Supplementary Materials. The numerical data represented in Figures are available upon request from the corresponding author.

## Supplementary Materials

The following supporting information can be downloaded at: https://www.mdpi.com/article/10.3390/molecules29102211/s1, Material for experimental procedures: Specific steps in the synthesis of the compound Table S1: Inhibition rate of biofilm by different compounds at different concentrations. Figures: The ^1^H-NMR and the ^13^C NMR spectrum of compounds.
