# Supplementary material for: Design, Synthesis, and Biological Evaluation of the Quorum-Sensing Inhibitors of Pseudomonas aeruginosa PAO1
Source: Molecules. 2024 May 8;29(10):2211. doi: 10.3390/molecules29102211 (PMC11123961; doi:10.3390/molecules29102211)
Supplement: Supplementary file 1 [file molecules-29-02211-s001.zip › Supplementary figures.pdf]

Supplemental information

Design, synthesis, and biological evaluation of the quorum-sensing inhibitors of *Pseudomonas aeruginosa* PAO1

1 National Engineering Research Center for the Emergency Drug, State Key Laboratory of Toxicology and Medical Countermeasures, Beijing Institute of Pharmacology and Toxicology, Beijing 100850, China; yanxinlin11@163.com (X.Y.) houshi28@126.com (S.H.); jiajiachang@126.com (J.C.);
2 School of Pharmaceutical Sciences, Jilin University, Changchun 130021, China; xing-cheng21@mails.jlu.edu.cn (C.X.)
3 School of Life Sciences, Jilin University, Changchun, 130012, China; yuanyuanz21@mails.jlu.edu.cn (Y.Z)
* Correspondence: xiaojunhai@139.com (J.X); Tel.:01066931634

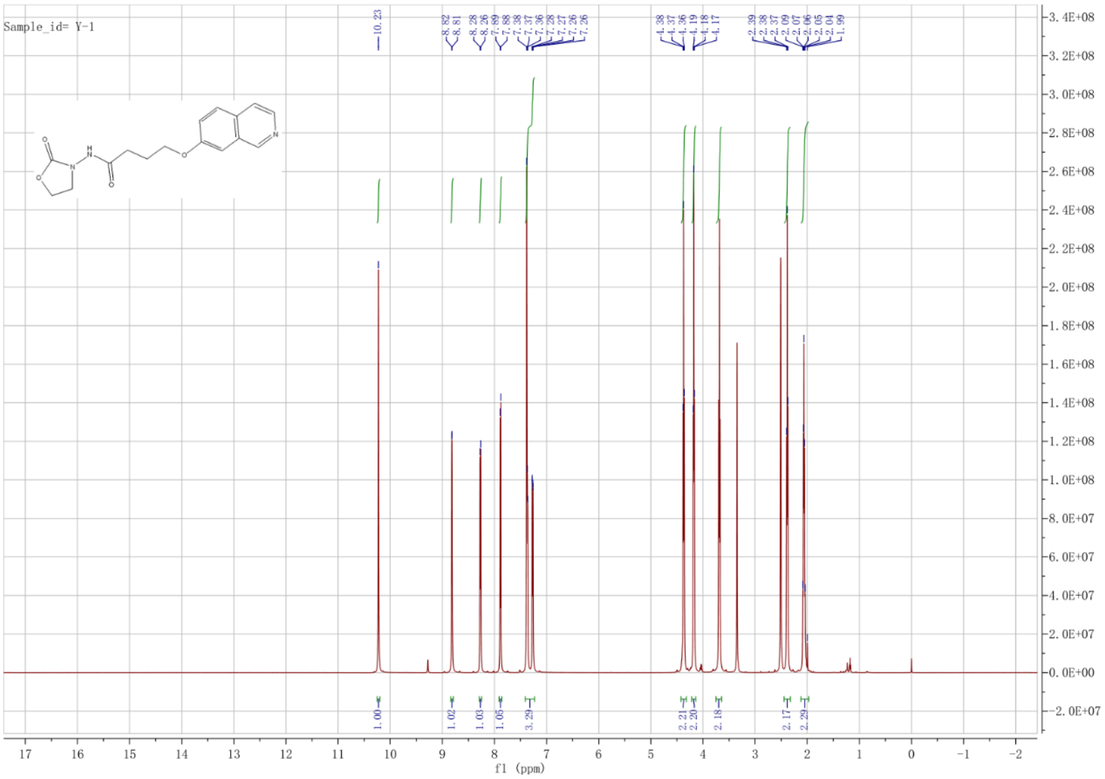

The ^{1}H-NMR spectrum of compound Y-1

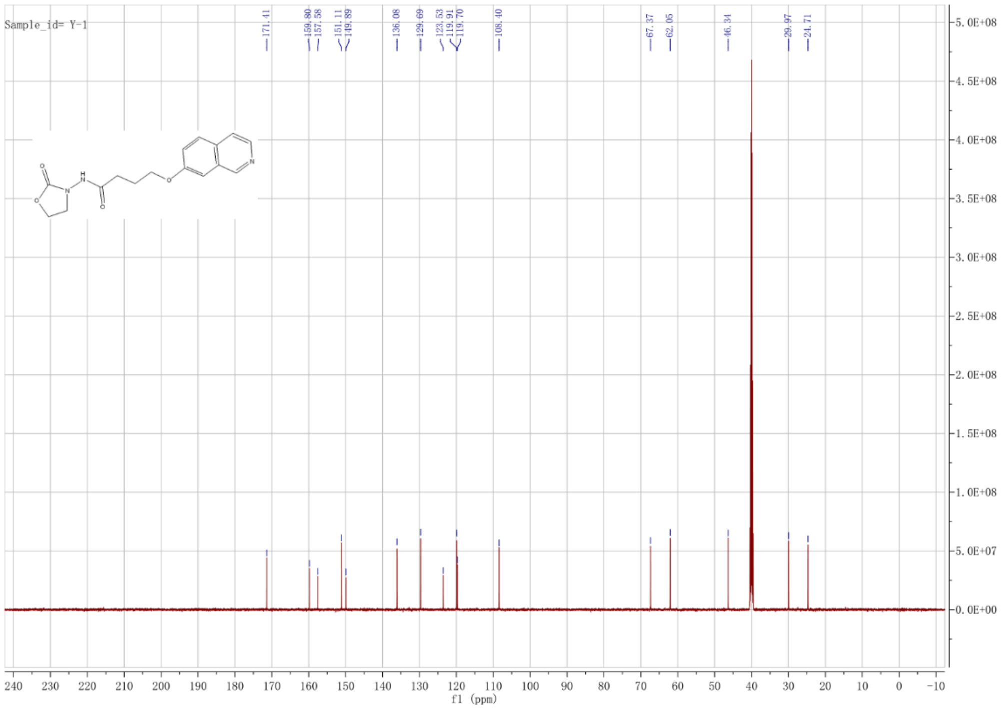

The ^{13}C NMR spectrum of compound Y-1

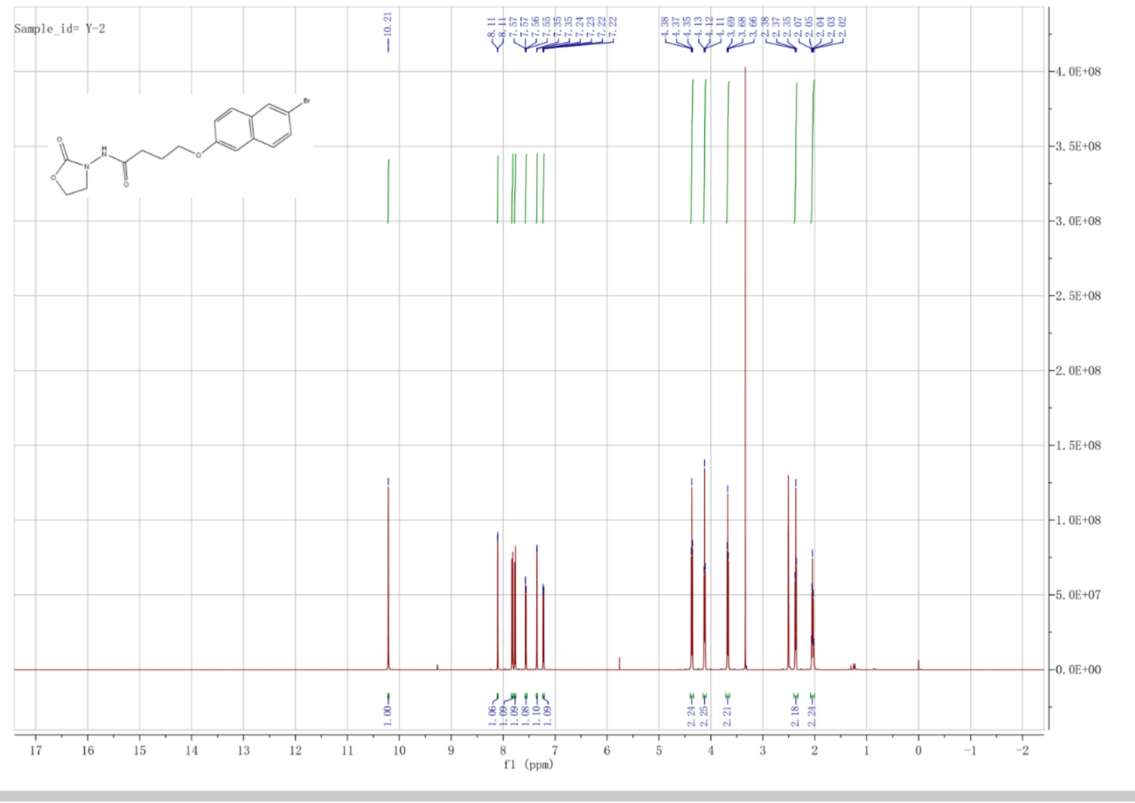

The ^{1}H-NMR spectrum of compound Y-2

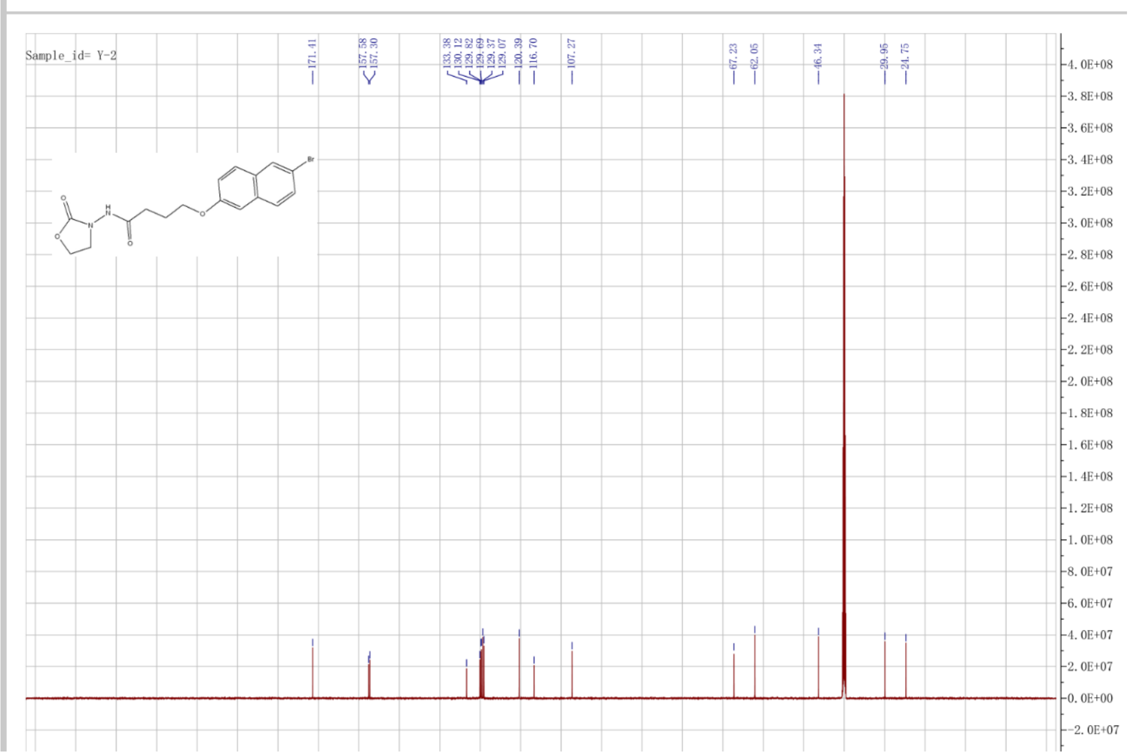

The ^{13}C NMR spectrum of compound Y-2

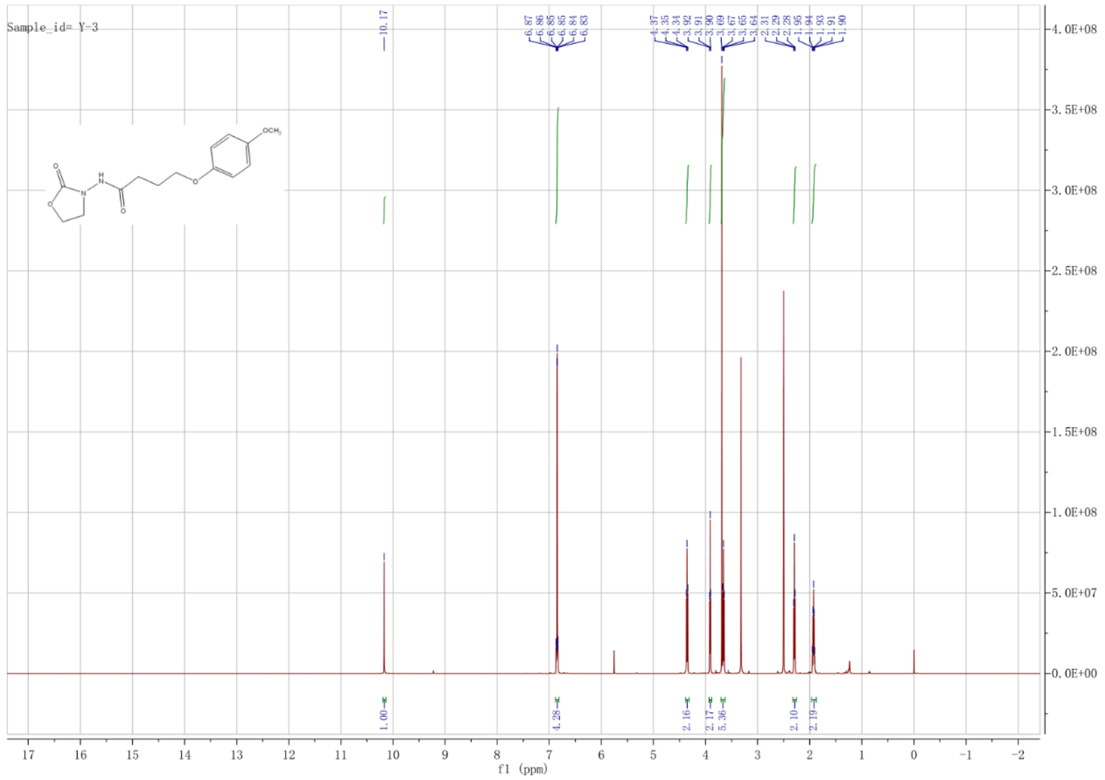

The ^{1}H-NMR spectrum of compound Y-3

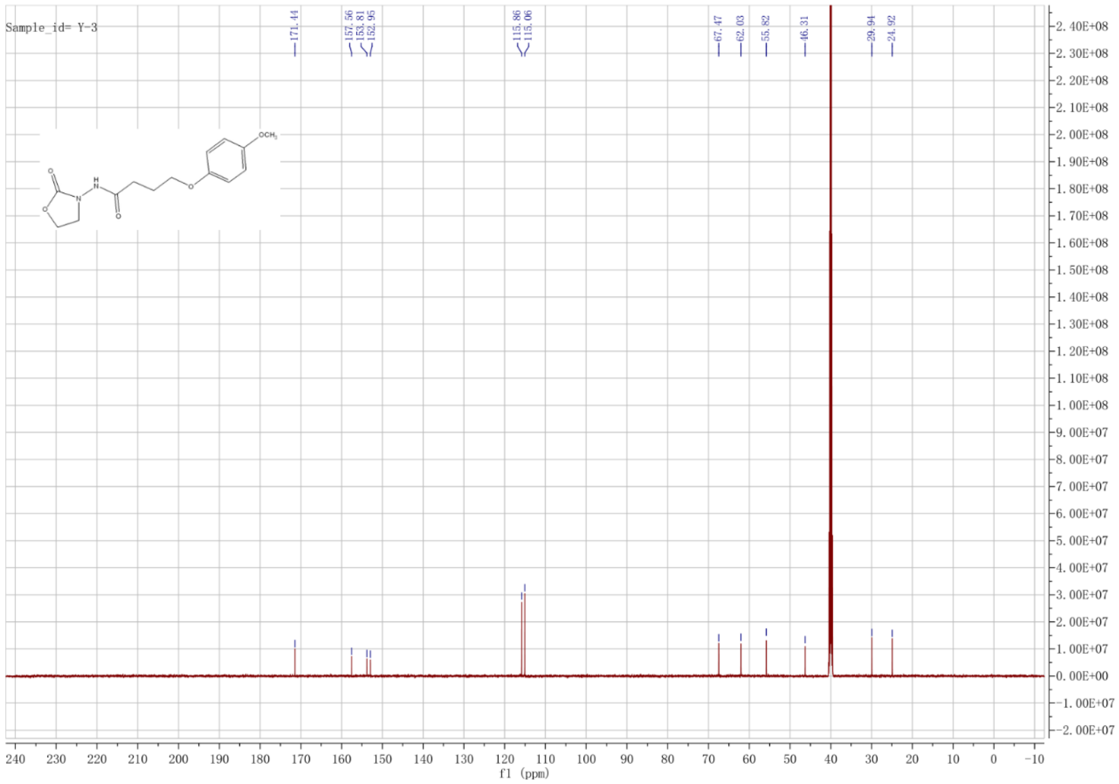

The ^{13}C NMR spectrum of compound Y-3

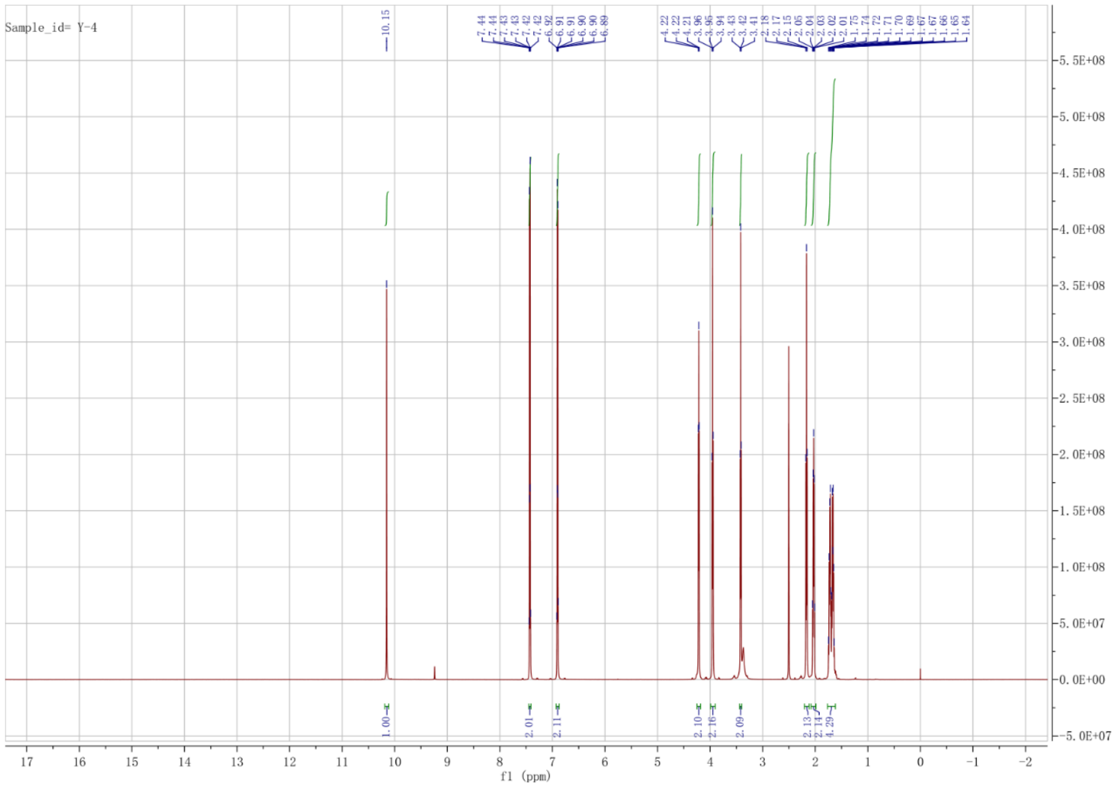

The ^{1}H-NMR spectrum of compound Y-4

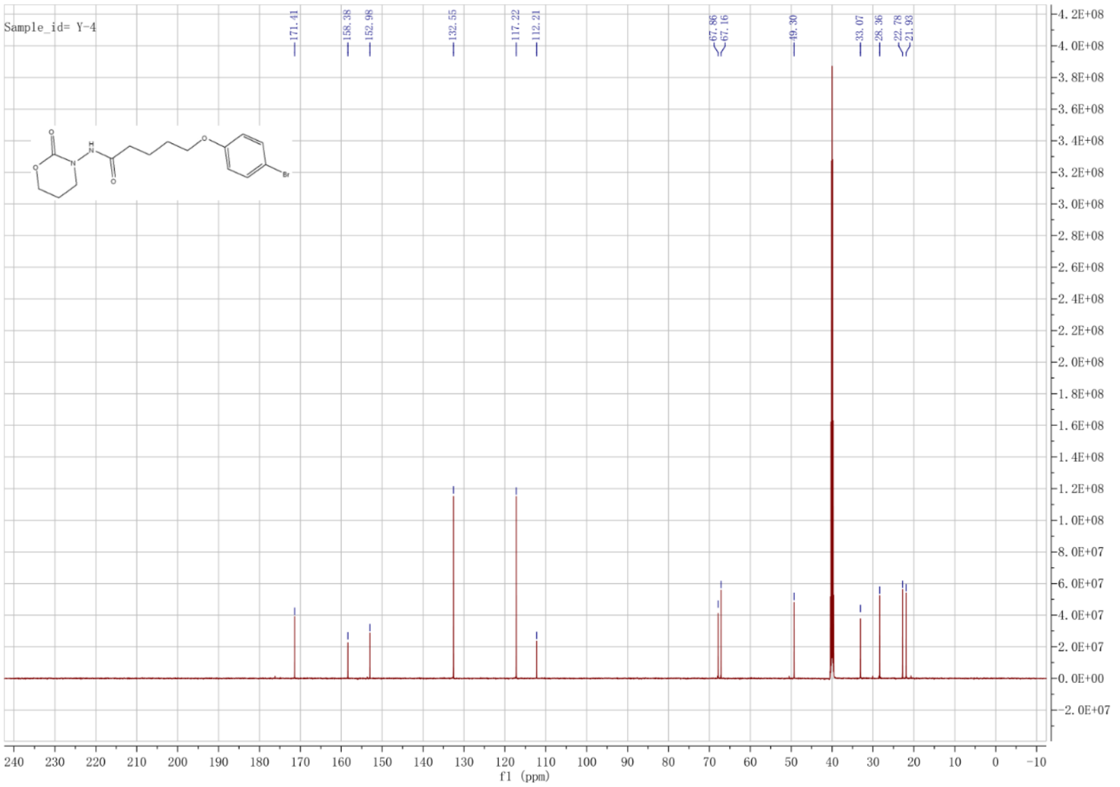

The ^{13}C NMR spectrum of compound Y-4

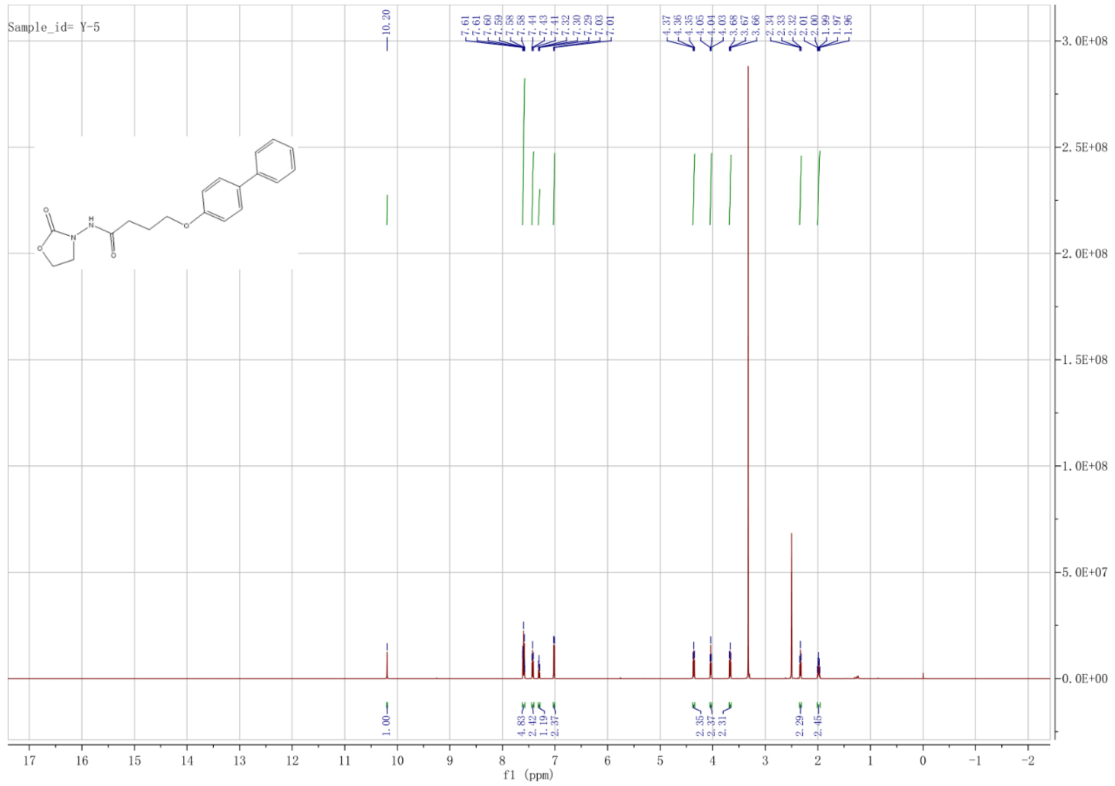

The ^{1}H-NMR spectrum of compound Y-5

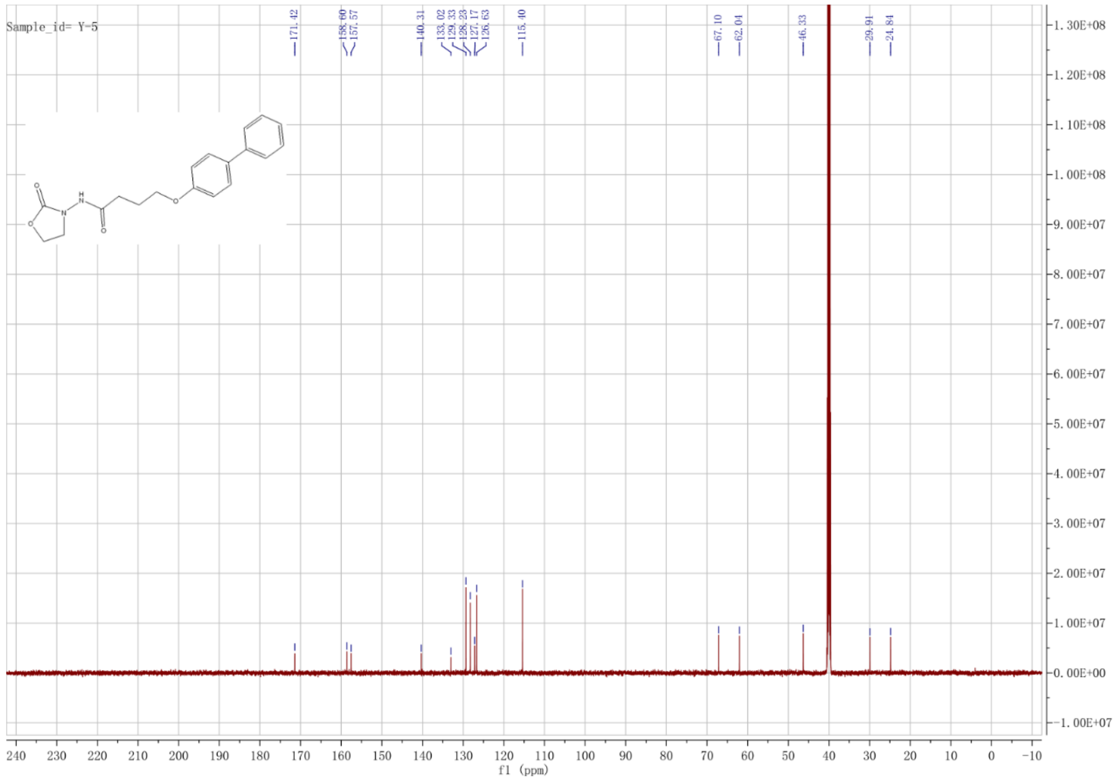

The ^{13}C NMR spectrum of compound Y-5

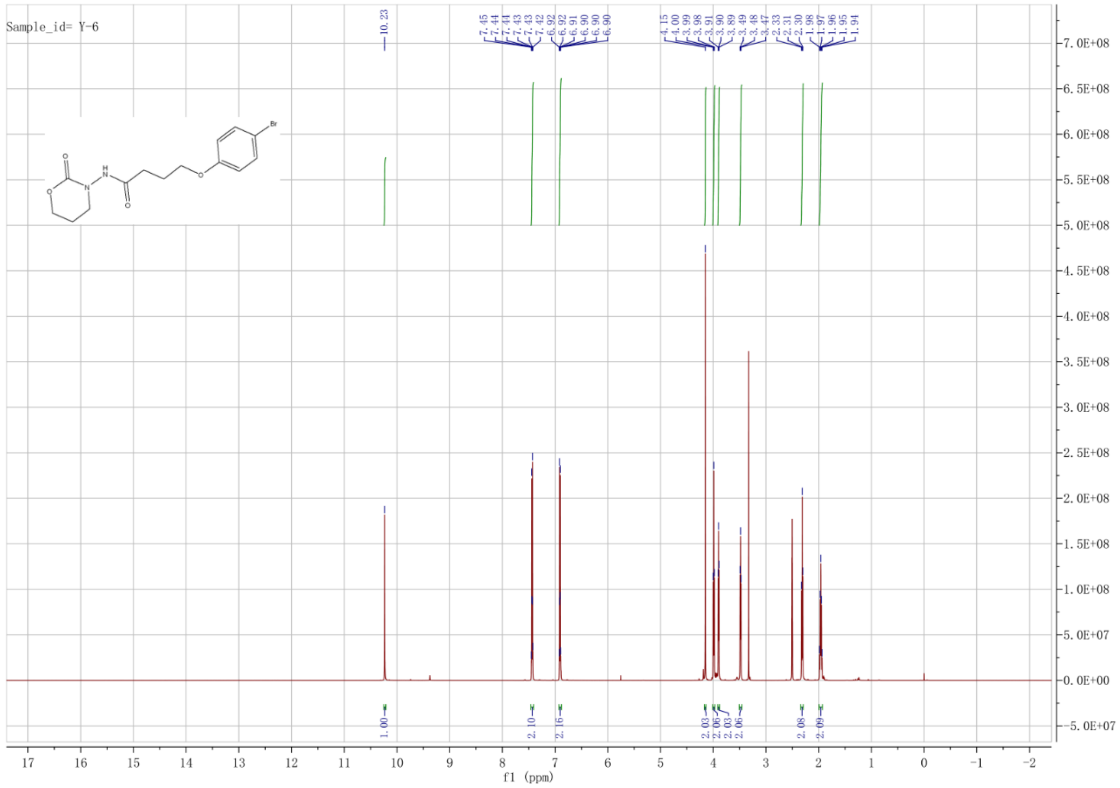

The ^{1}H-NMR spectrum of compound Y-6

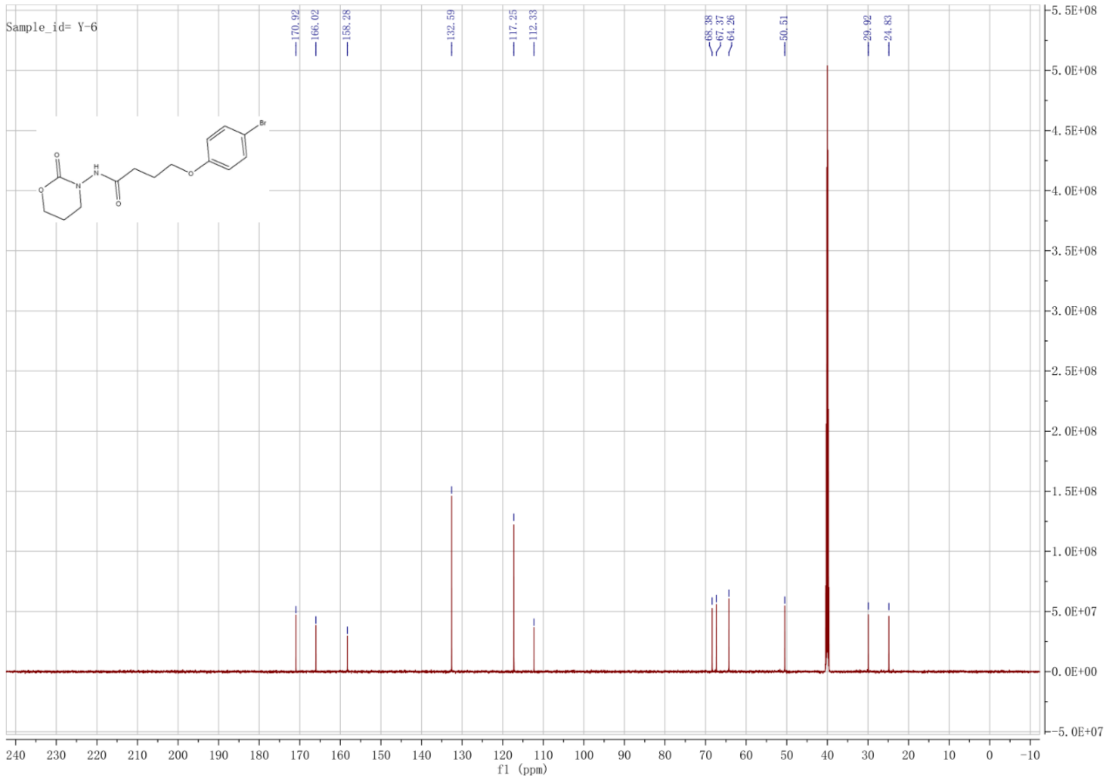

The ^{13}C NMR spectrum of compound Y-6

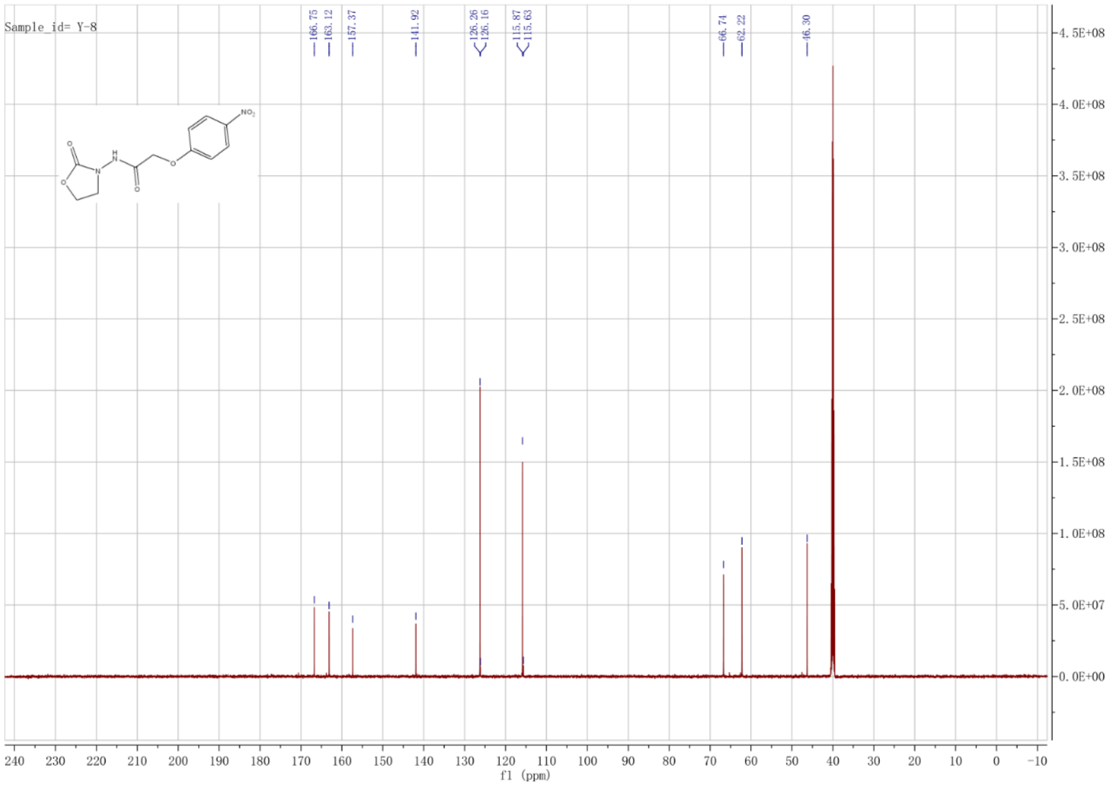

The ^{1}H-NMR spectrum of compound Y-8

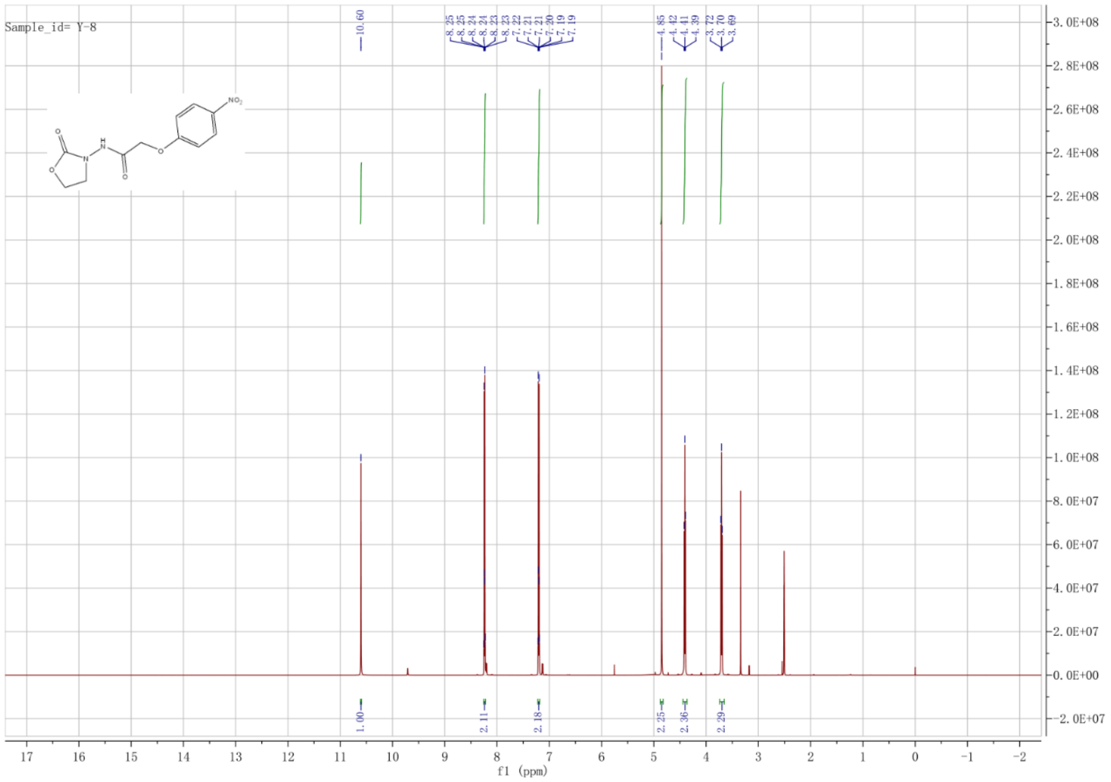

The ^{13}C NMR spectrum of compound Y-8

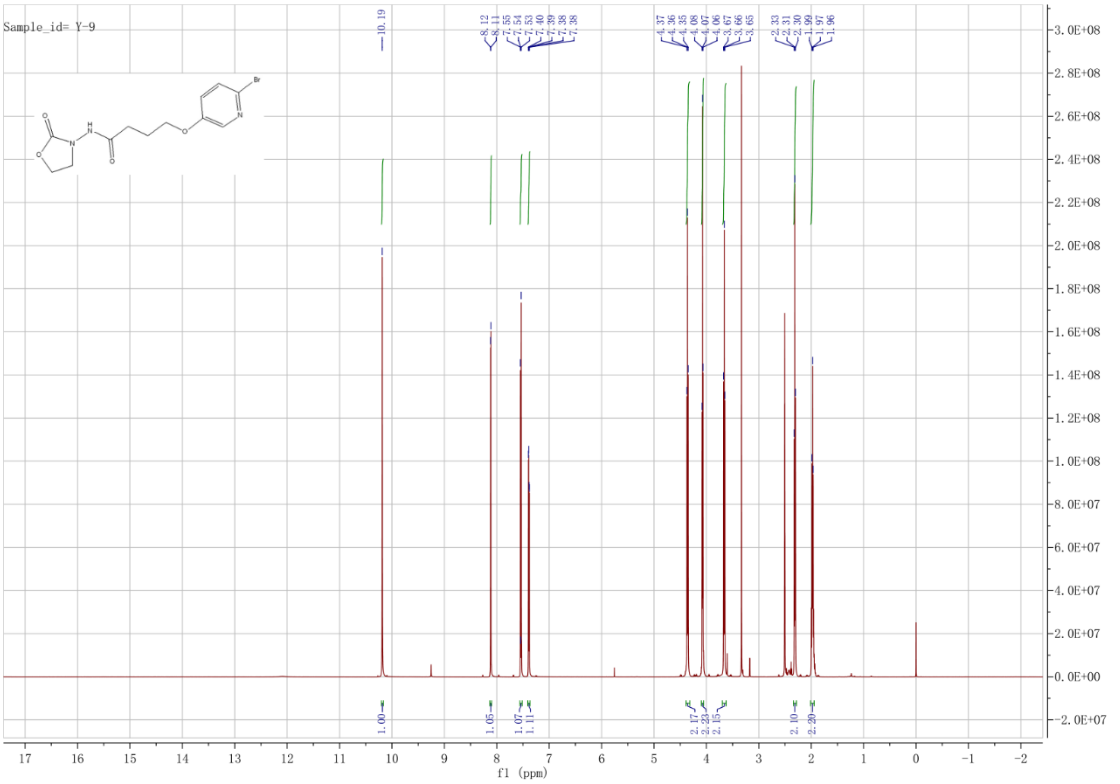

The ^{1}H-NMR spectrum of compound Y-9

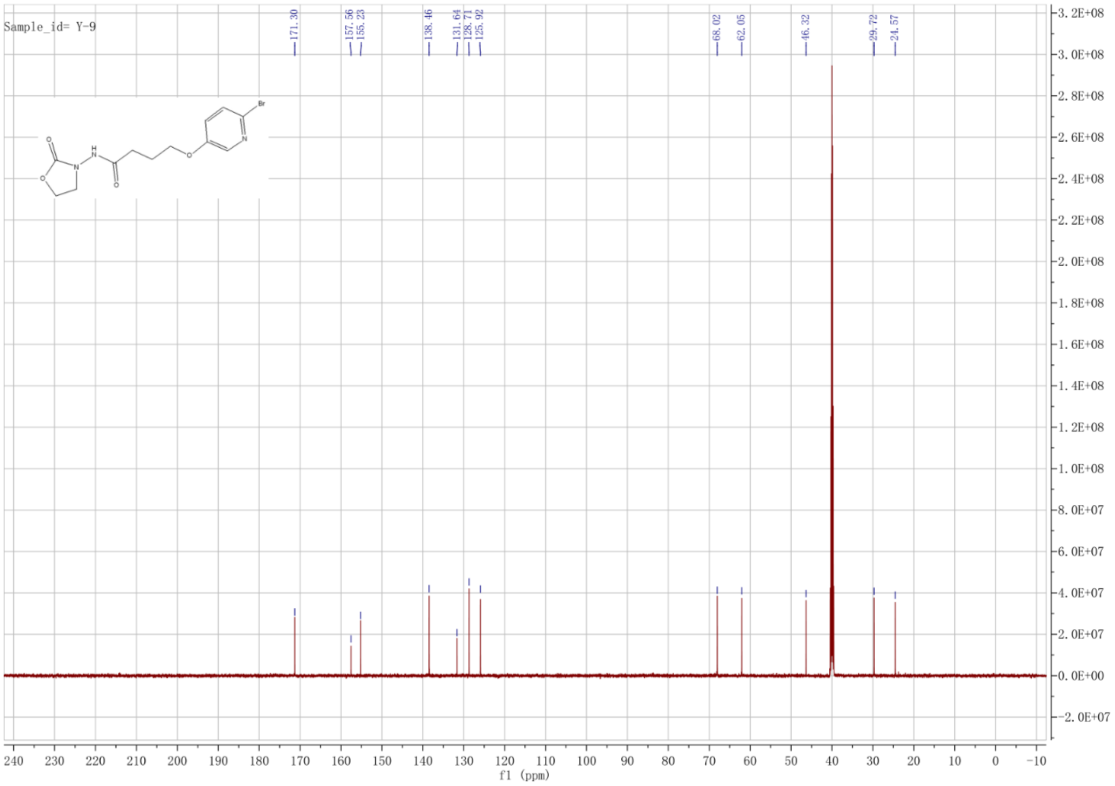

The ^{13}C NMR spectrum of compound Y-9

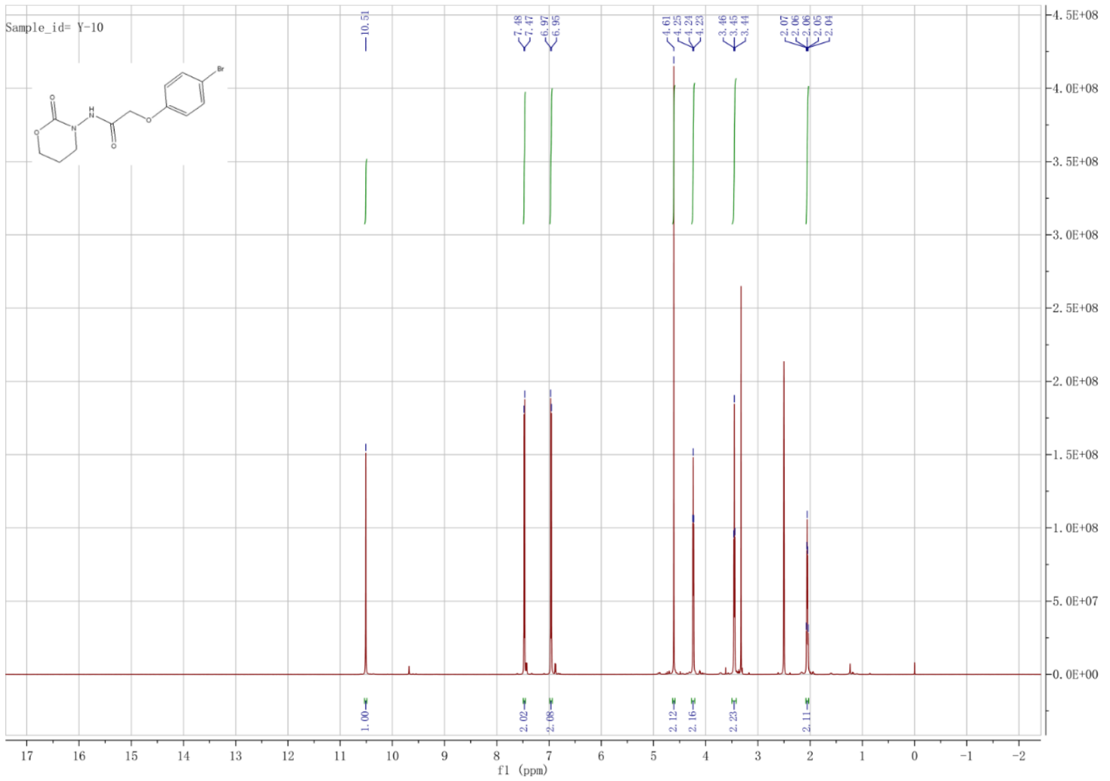

The ^{1}H-NMR spectrum of compound Y-10

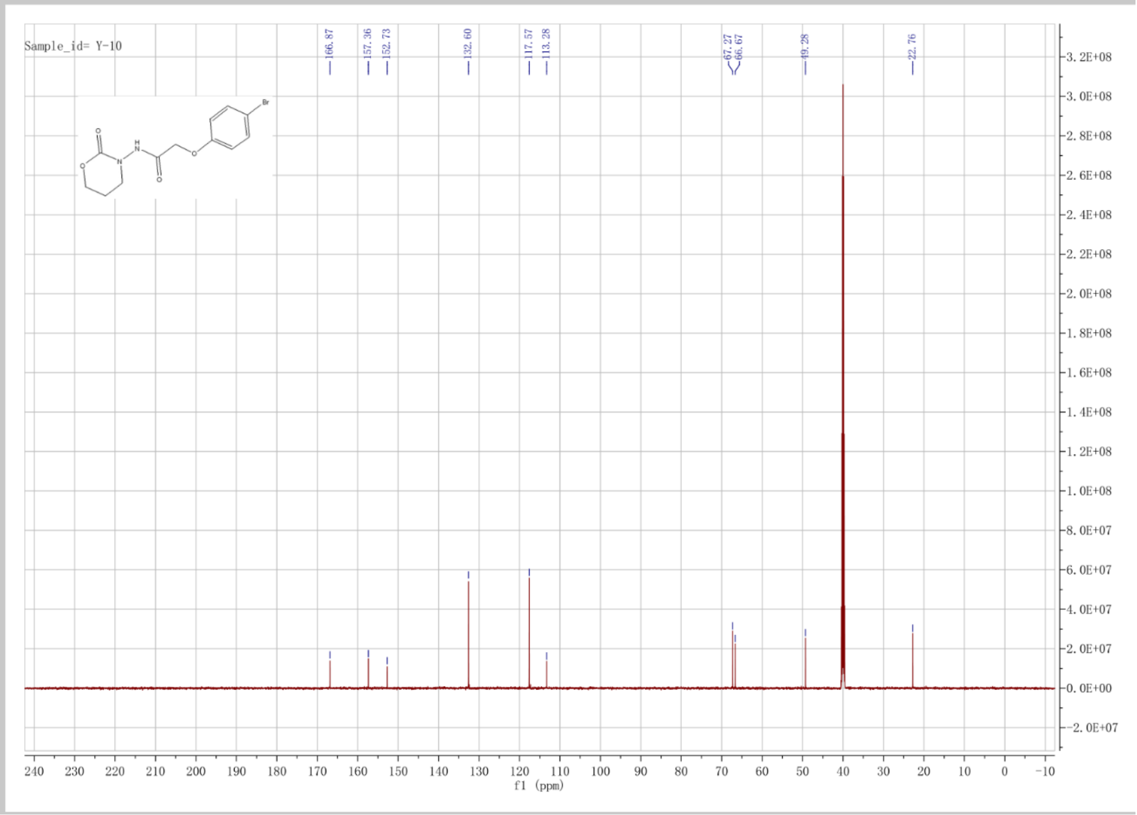

The ^{13}C NMR spectrum of compound Y-10

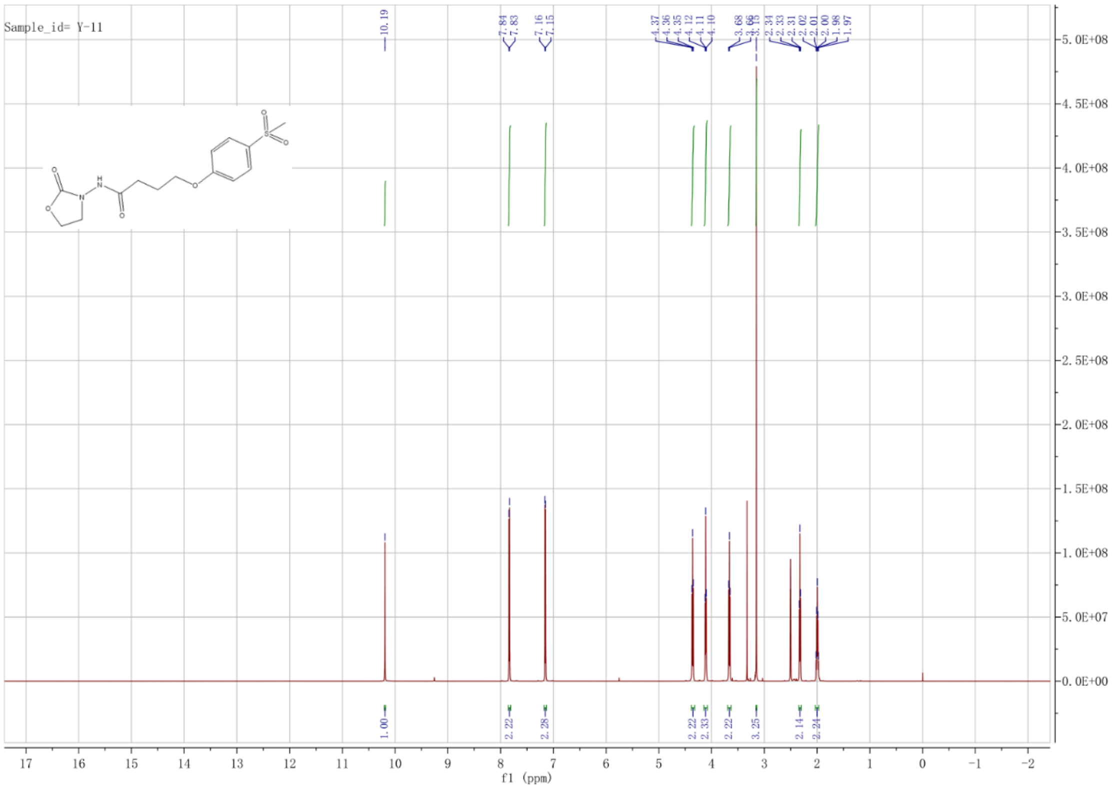

The ^{1}H-NMR spectrum of compound Y-11

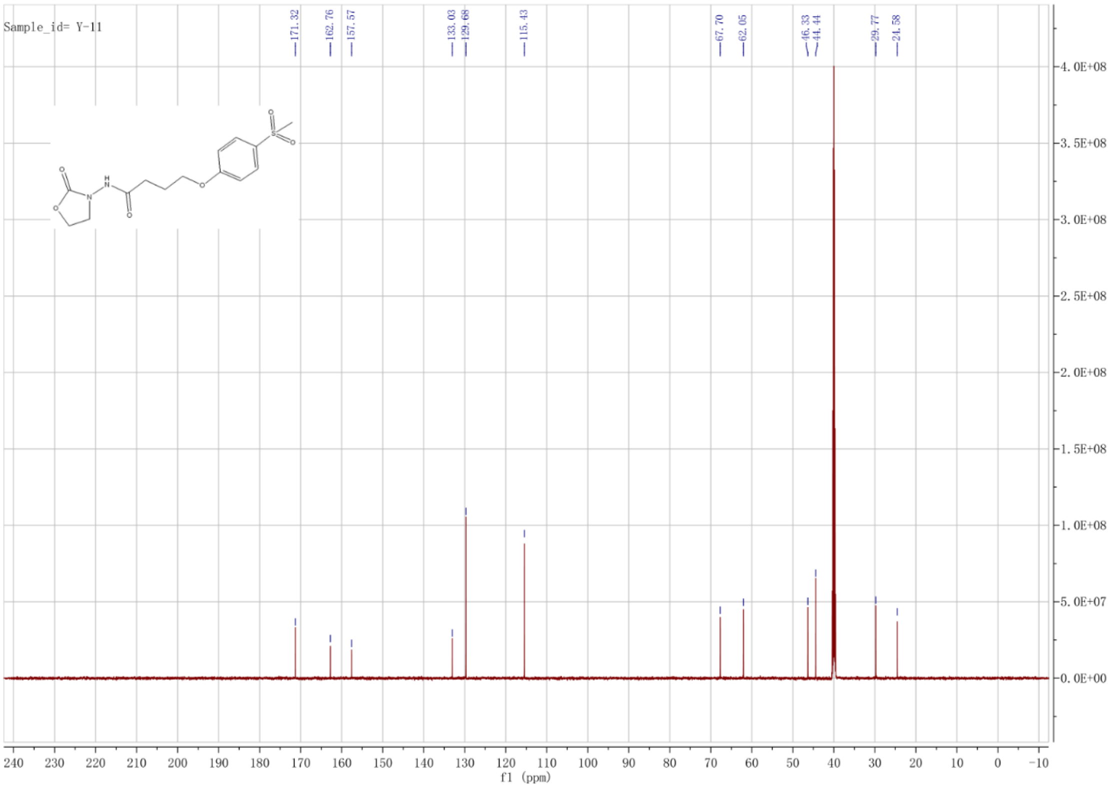

The ^{13}C NMR spectrum of compound Y-11

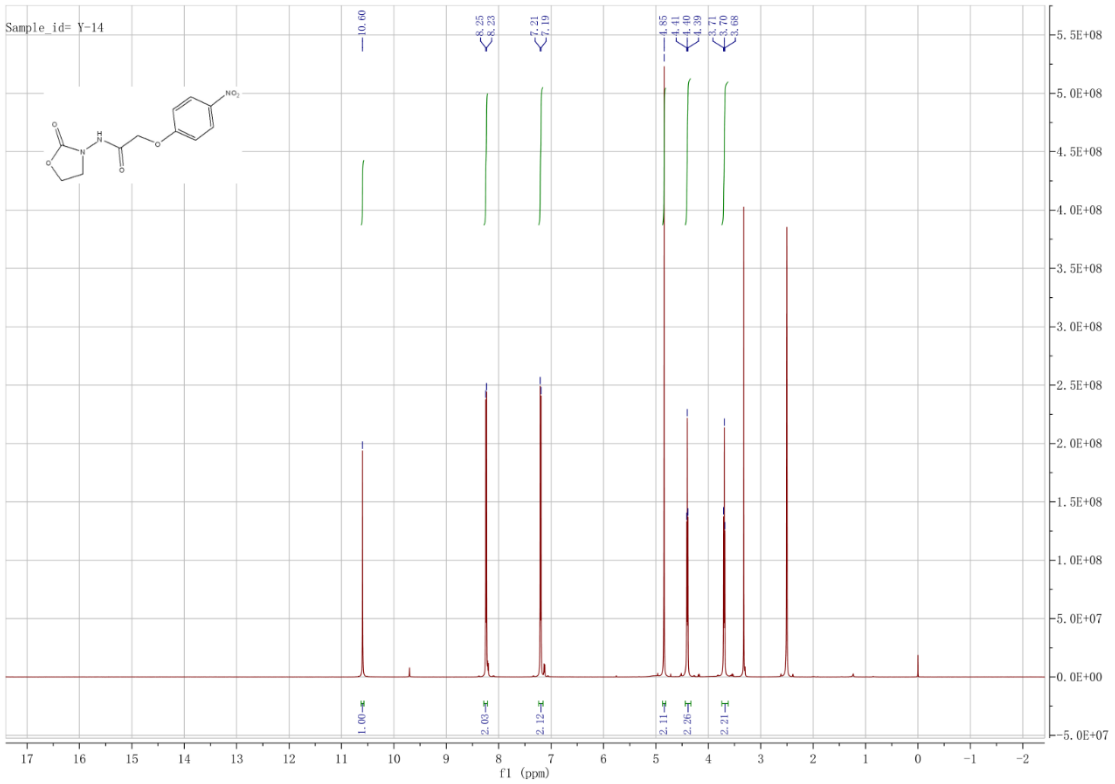

The 1H-NMR spectrum of compound Y-14

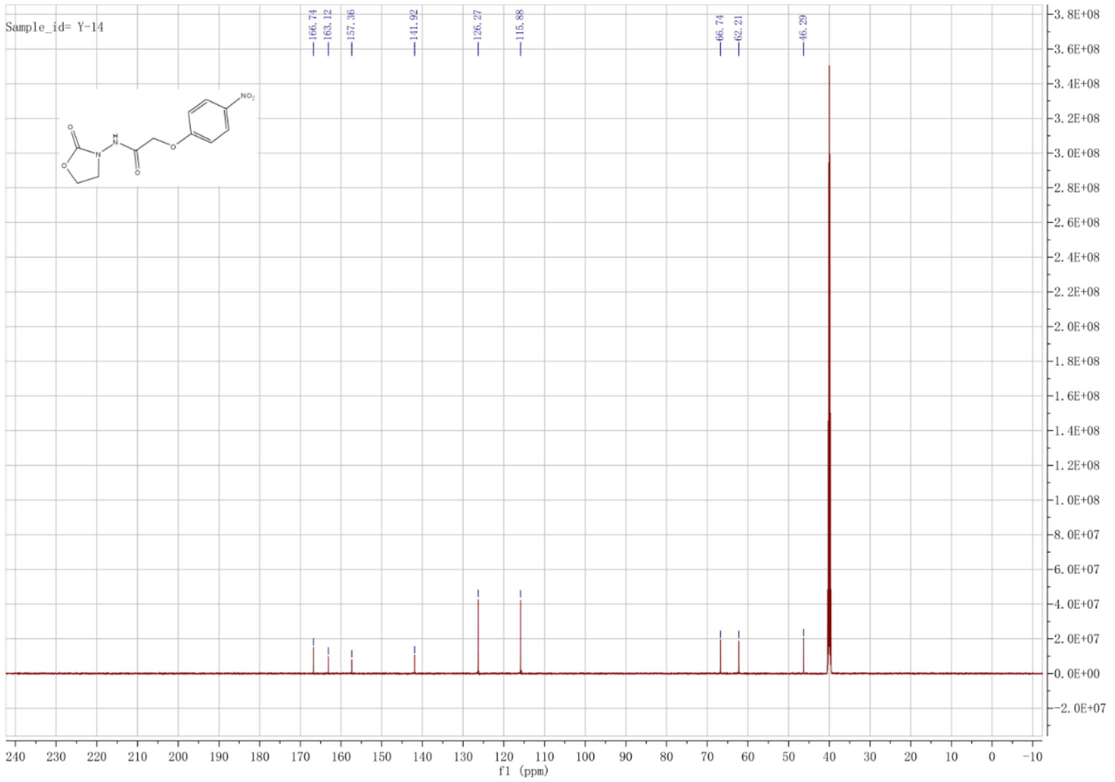

The ^{13}C NMR spectrum of compound Y-14

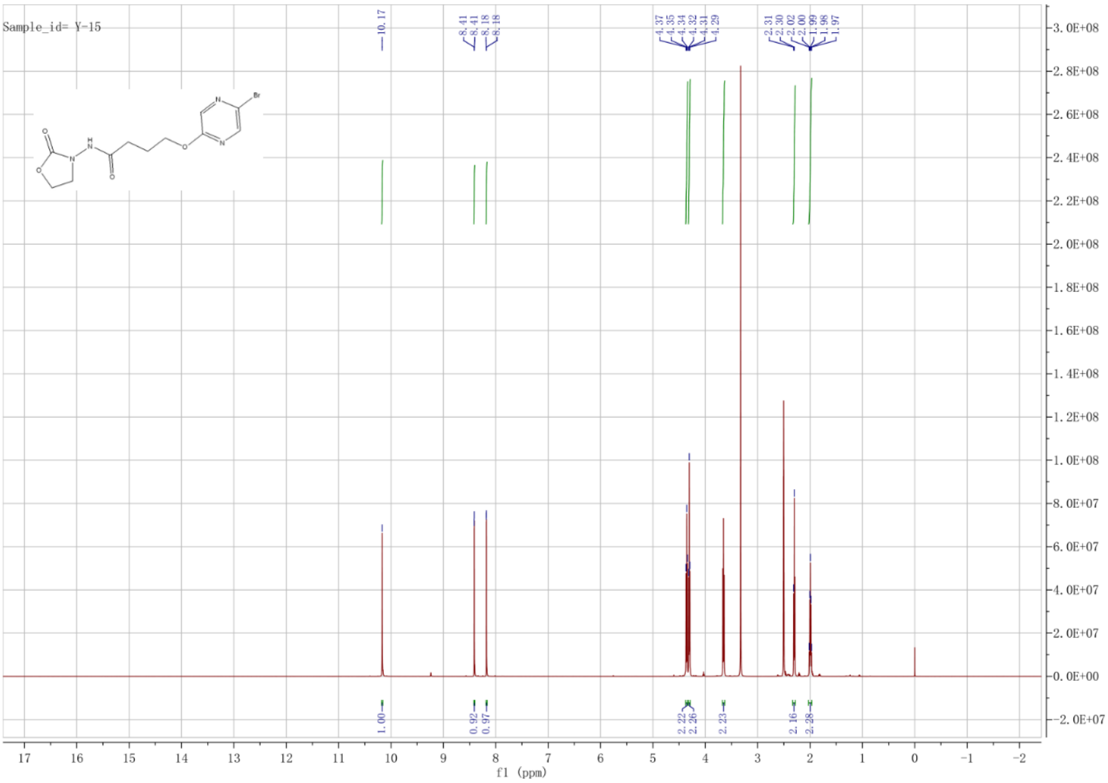

The 1H-NMR spectrum of compound Y-15

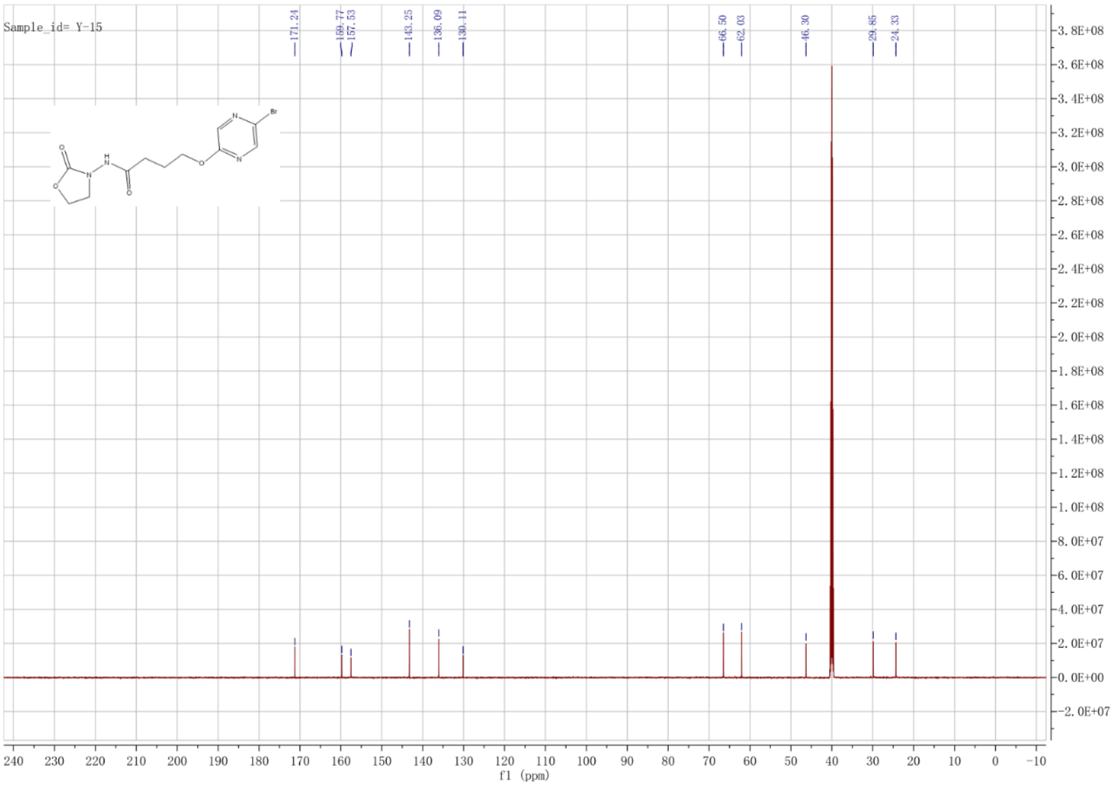

The ^{13}C NMR spectrum of compound Y-15

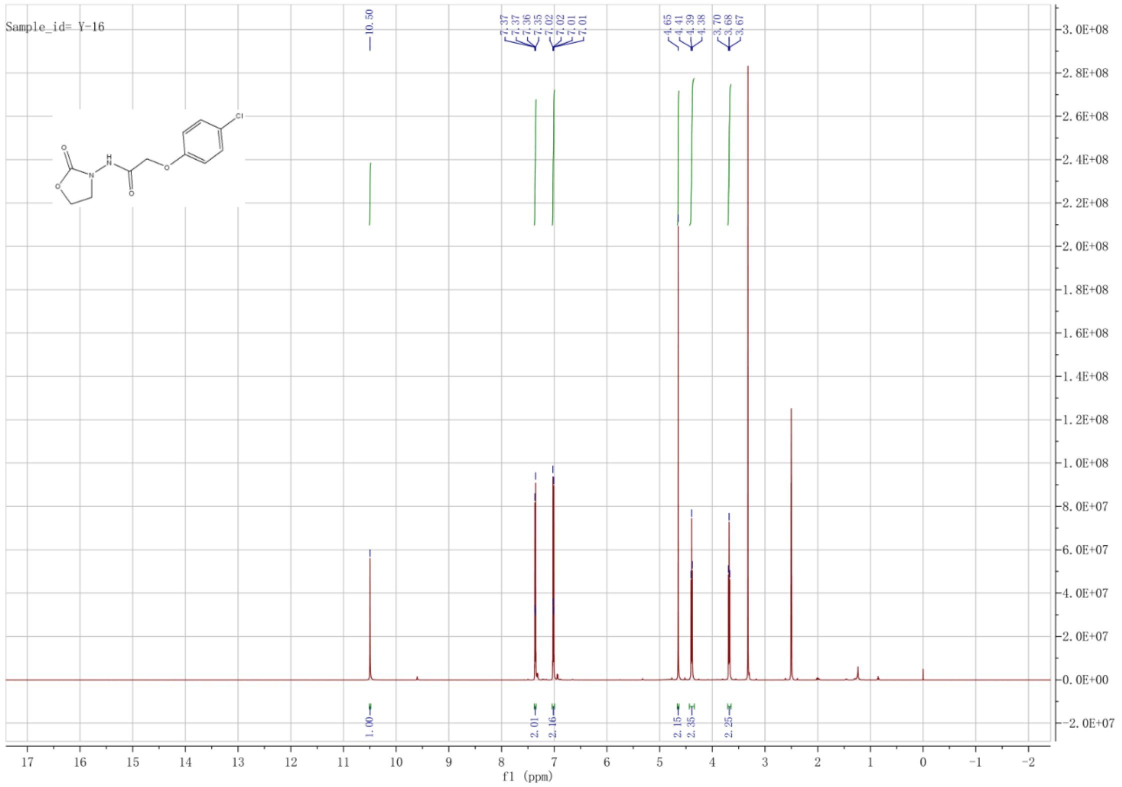

The 1H-NMR spectrum of compound Y-16

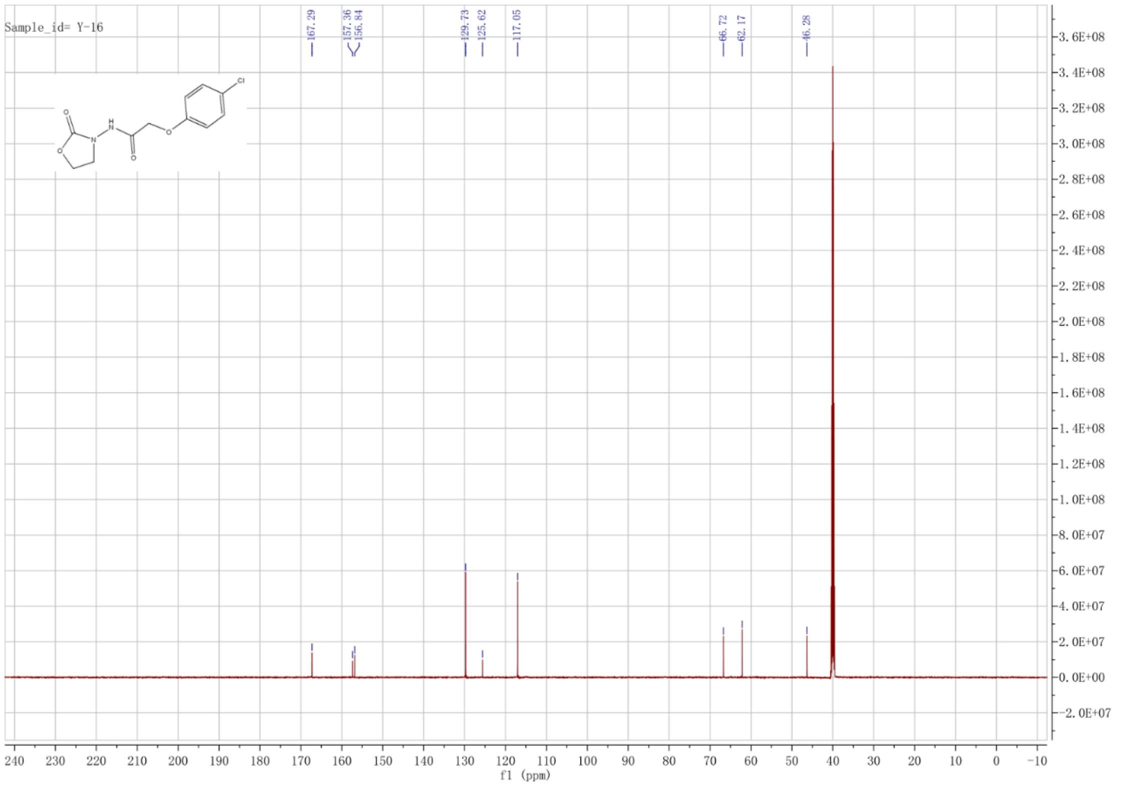

The ^{13}C NMR spectrum of compound Y-16

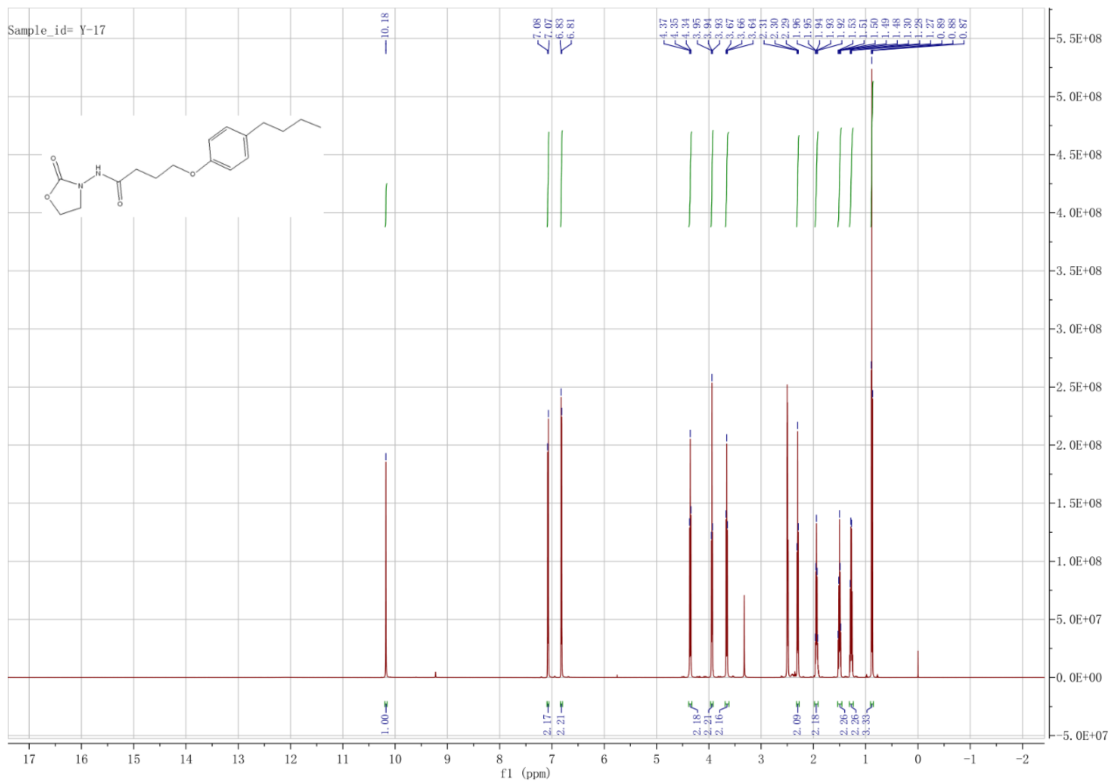

The 1H-NMR spectrum of compound Y-17

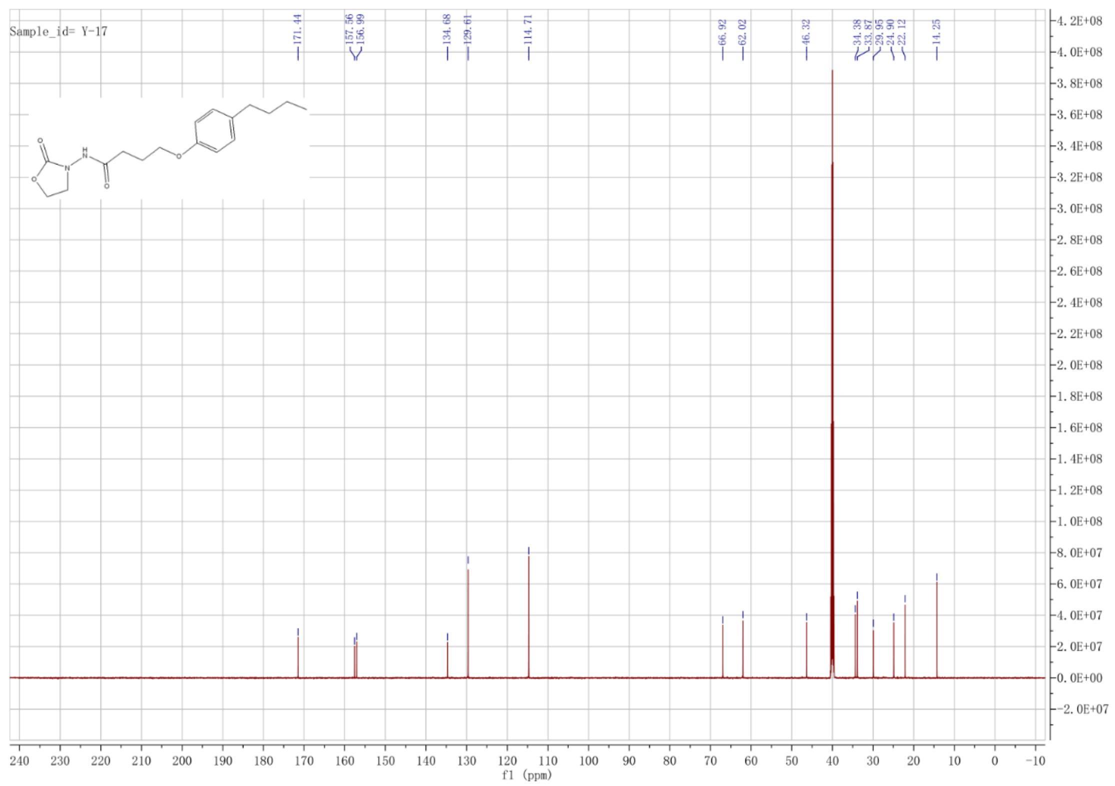

The ^{13}C NMR spectrum of compound Y-17

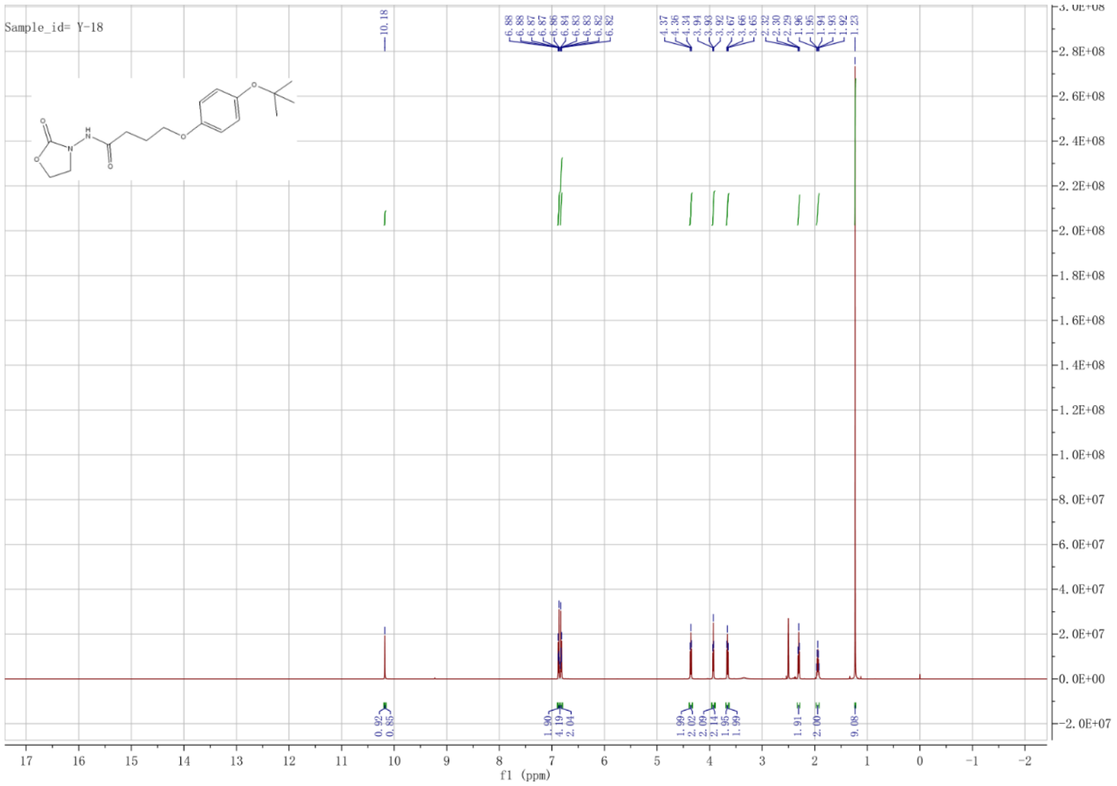

The 1H-NMR spectrum of compound Y-18

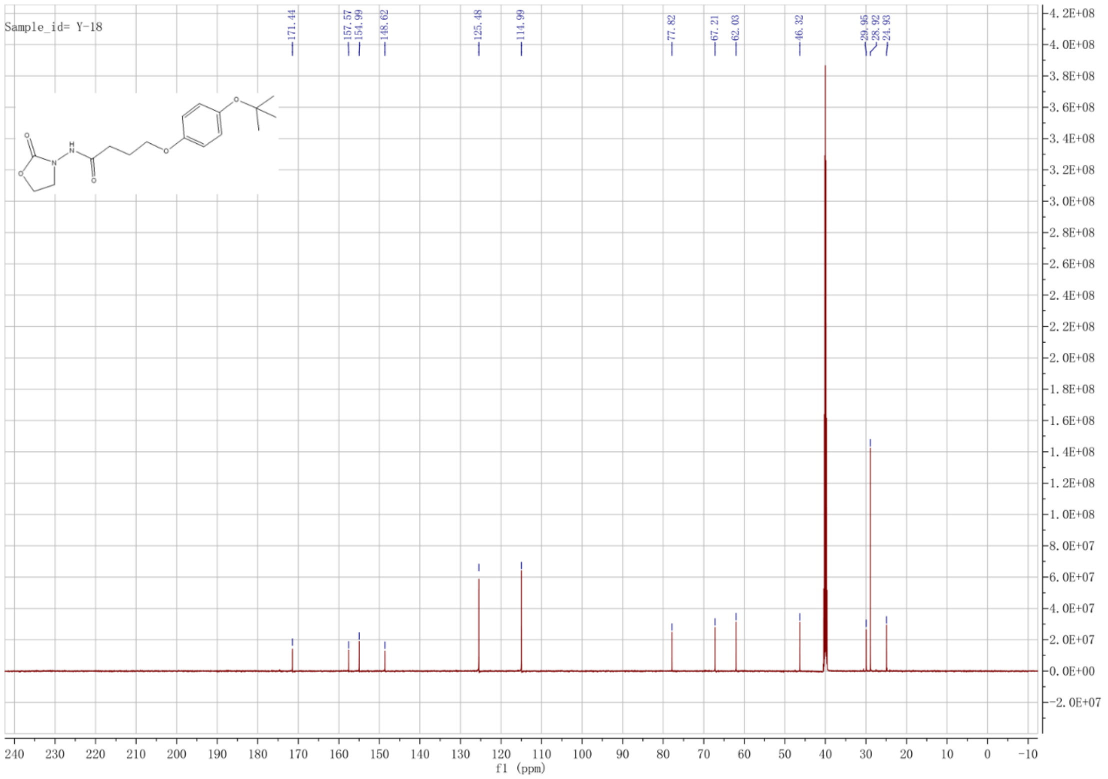

The ^{13}C NMR spectrum of compound Y-18

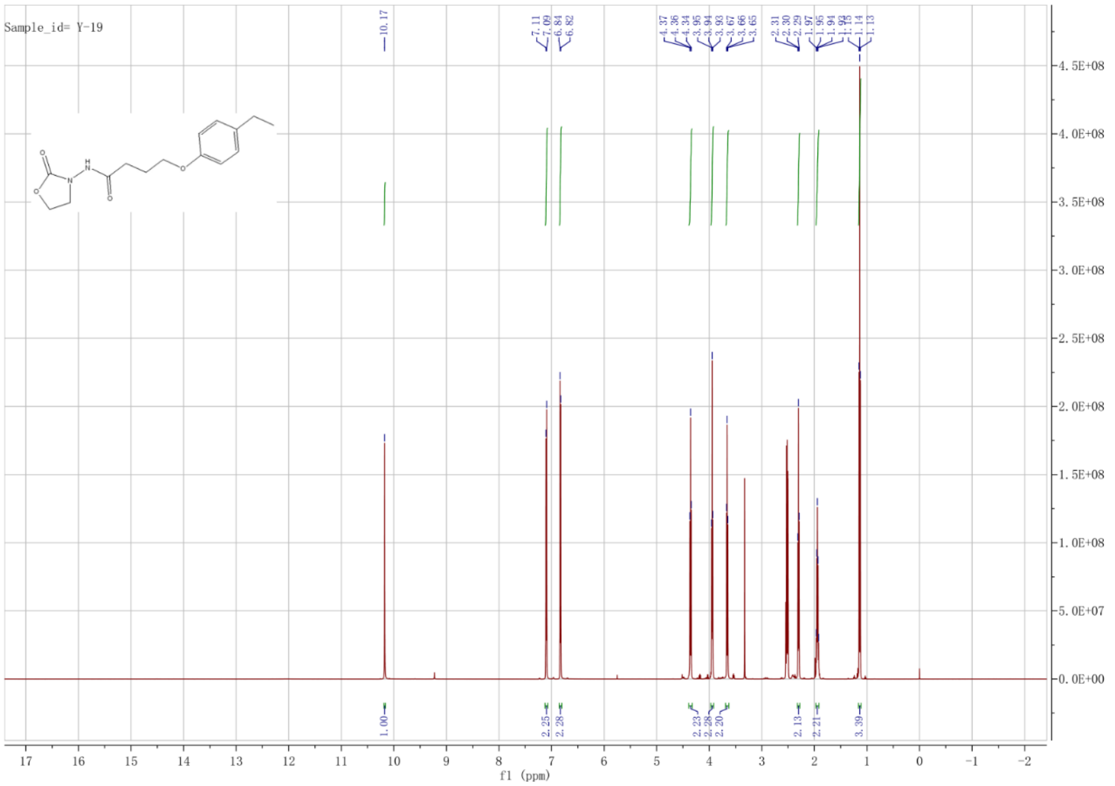

The 1H-NMR spectrum of compound Y-19

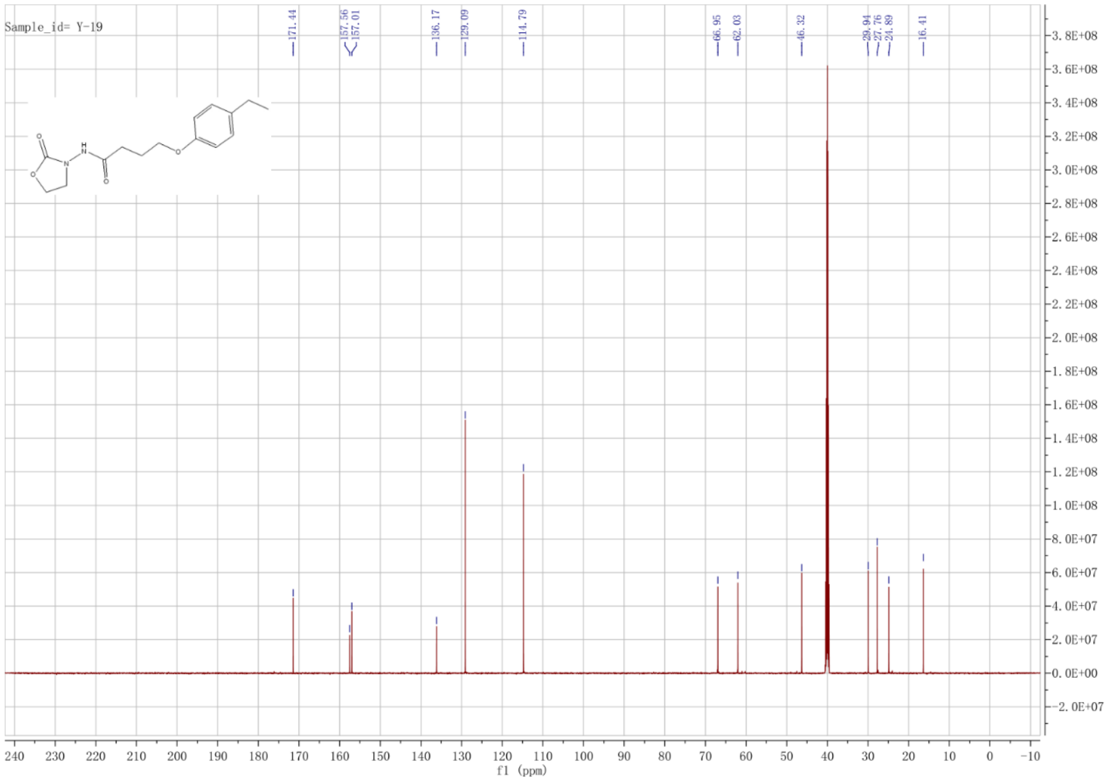

The ^{13}C NMR spectrum of compound Y-19

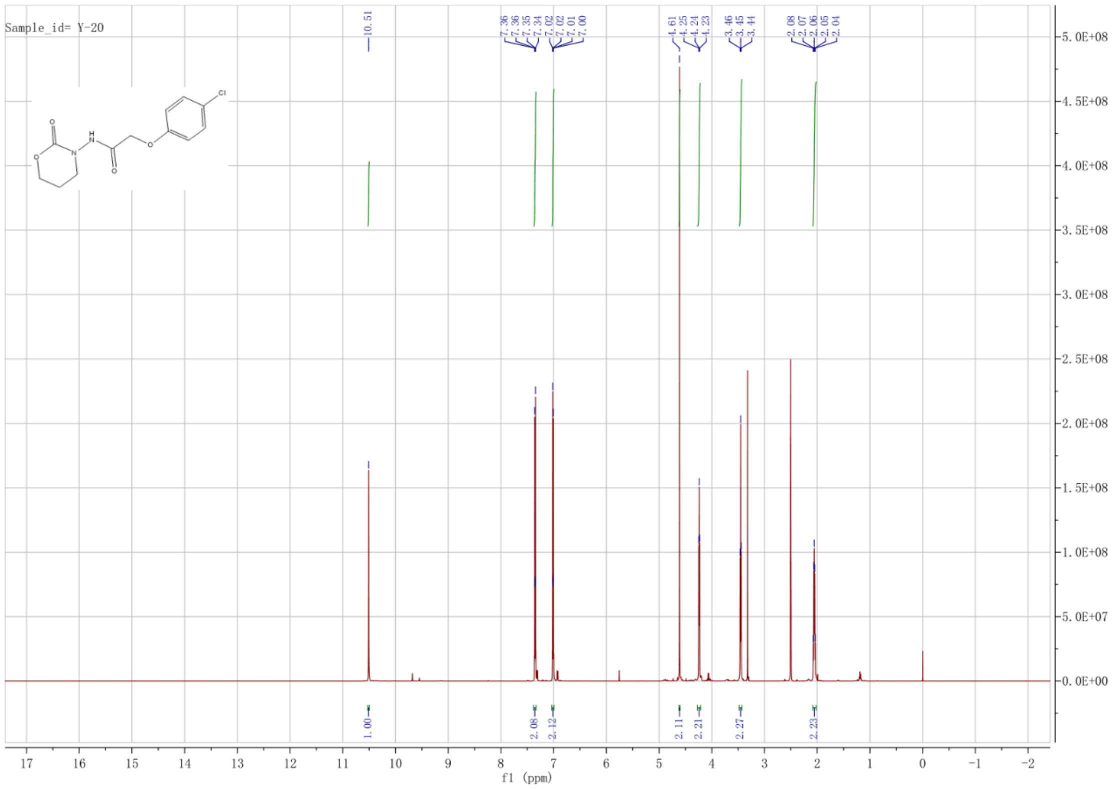

The 1H-NMR spectrum of compound Y-20

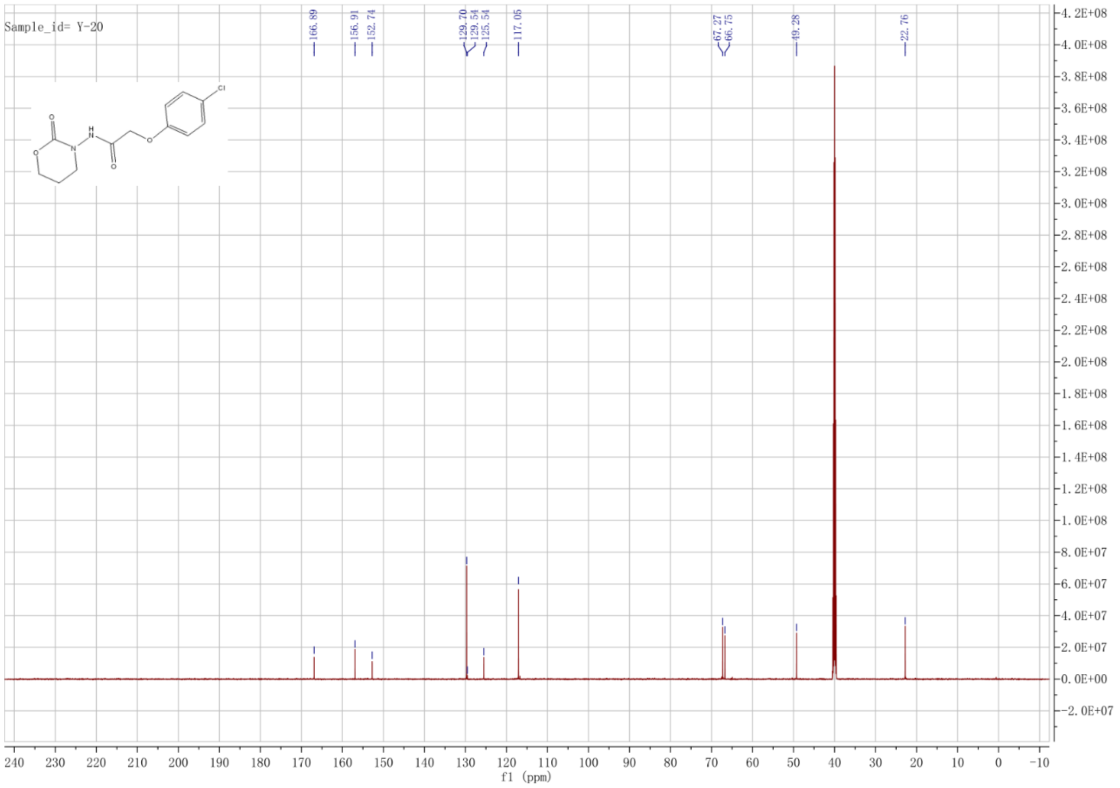

The ^{13}C NMR spectrum of compound Y-20

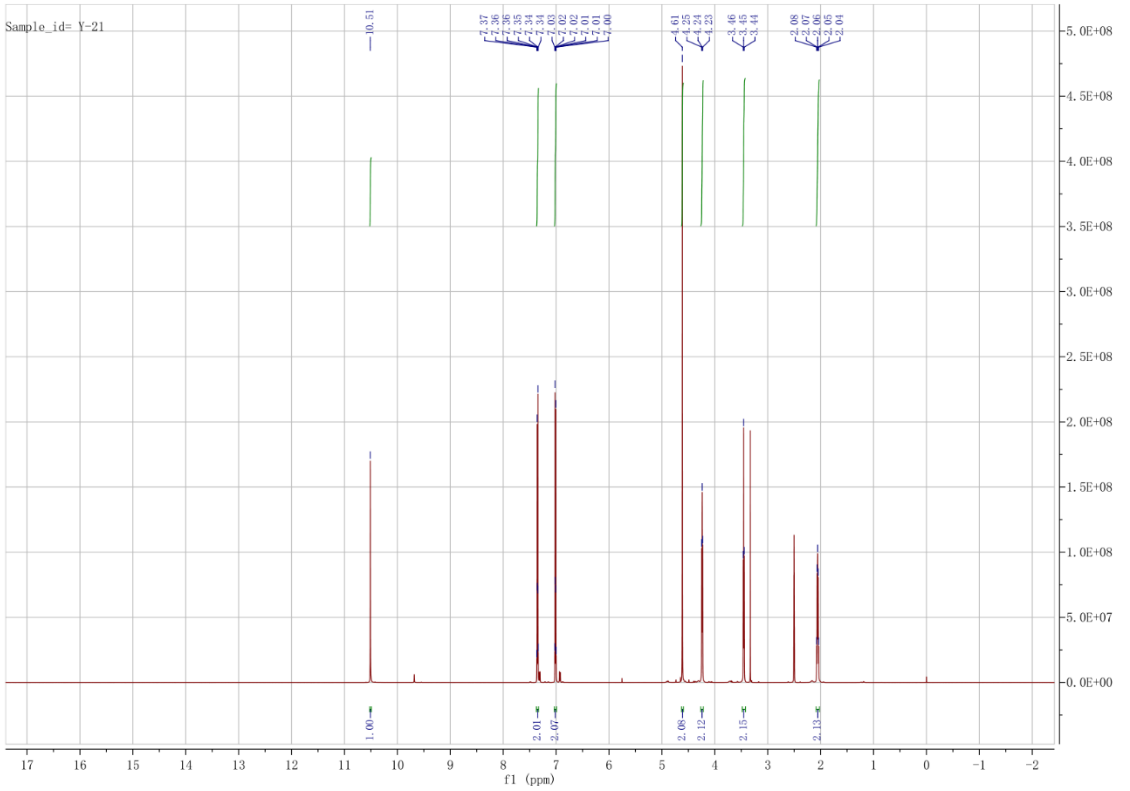

The 1H-NMR spectrum of compound Y-21

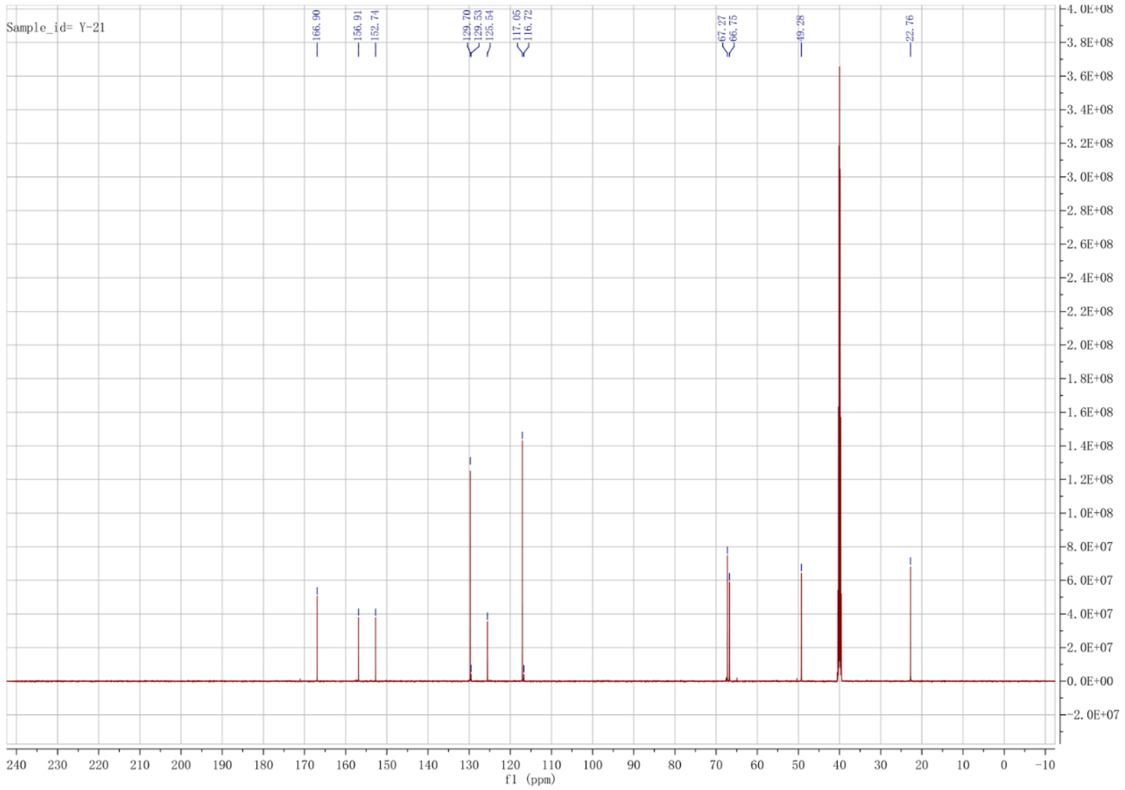

The ^{13}C NMR spectrum of compound Y-21

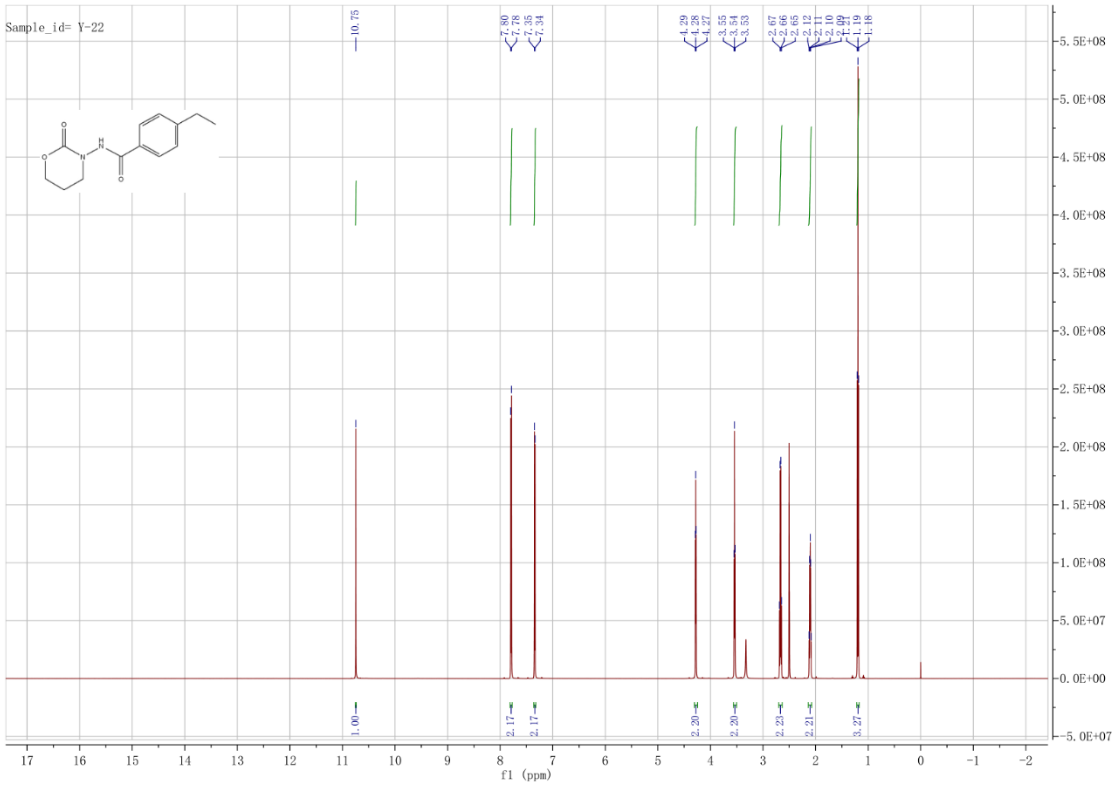

The 1H-NMR spectrum of compound Y-22

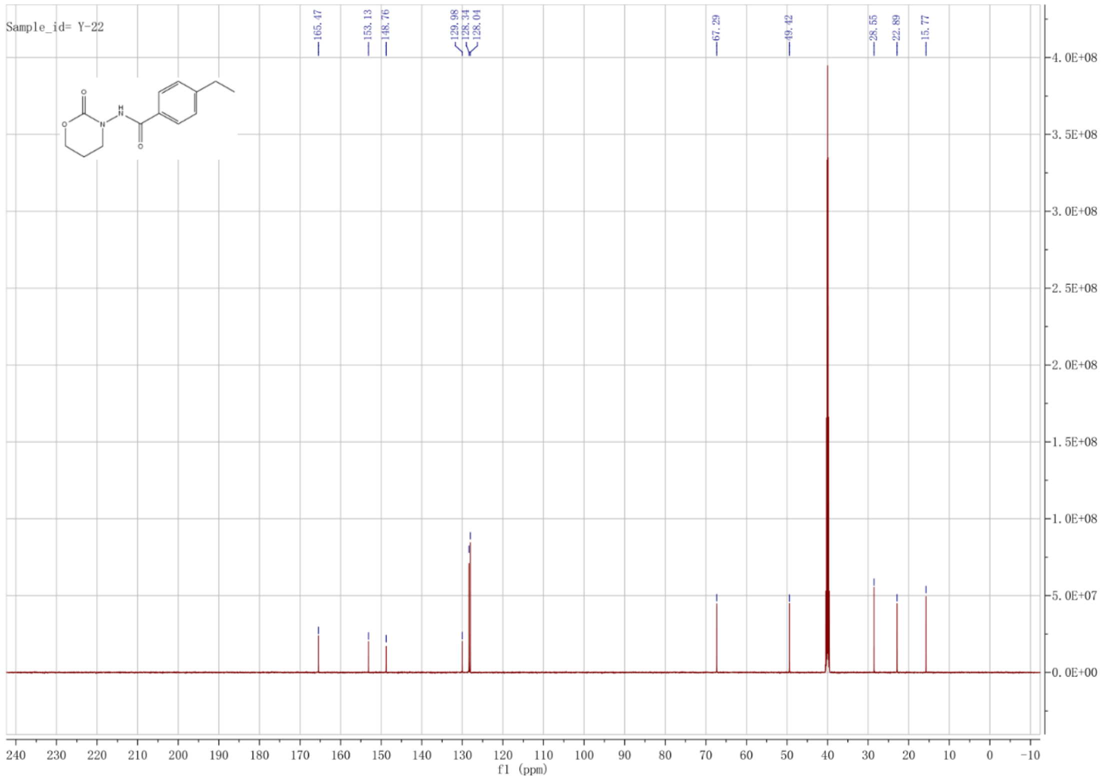

The ^{13}C NMR spectrum of compound Y-22

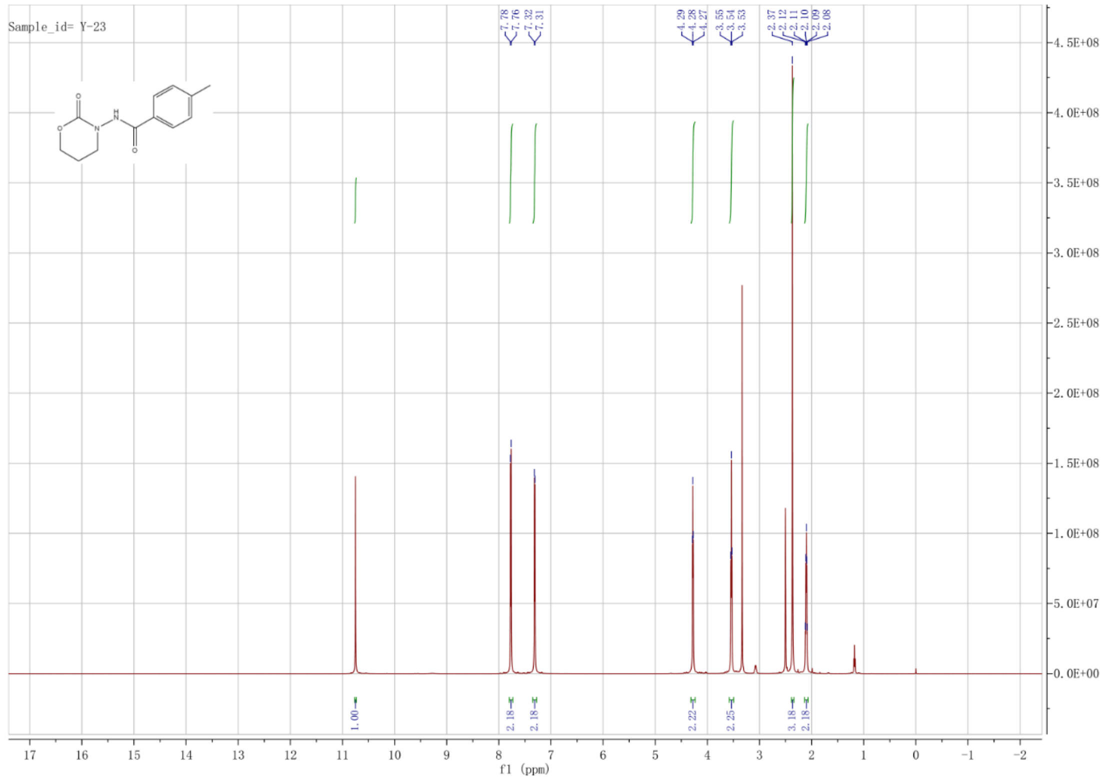

The 1H-NMR spectrum of compound Y-23

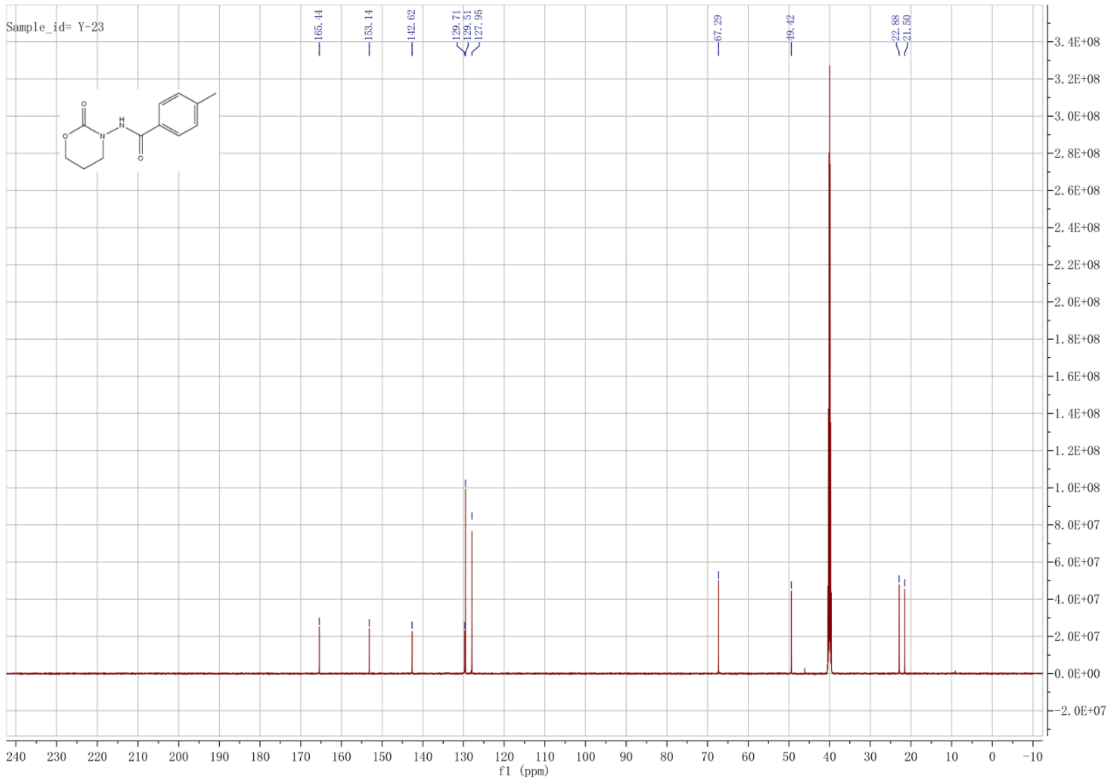

The ^{13}C NMR spectrum of compound Y-23

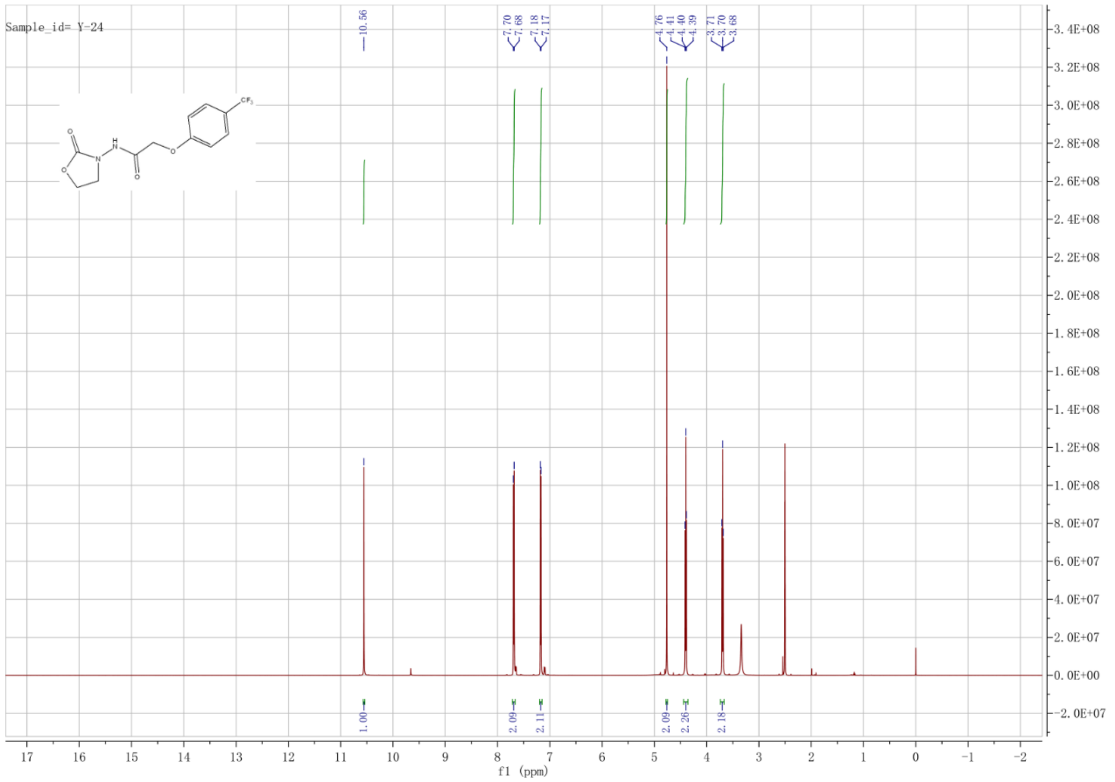

The 1H-NMR spectrum of compound Y-24

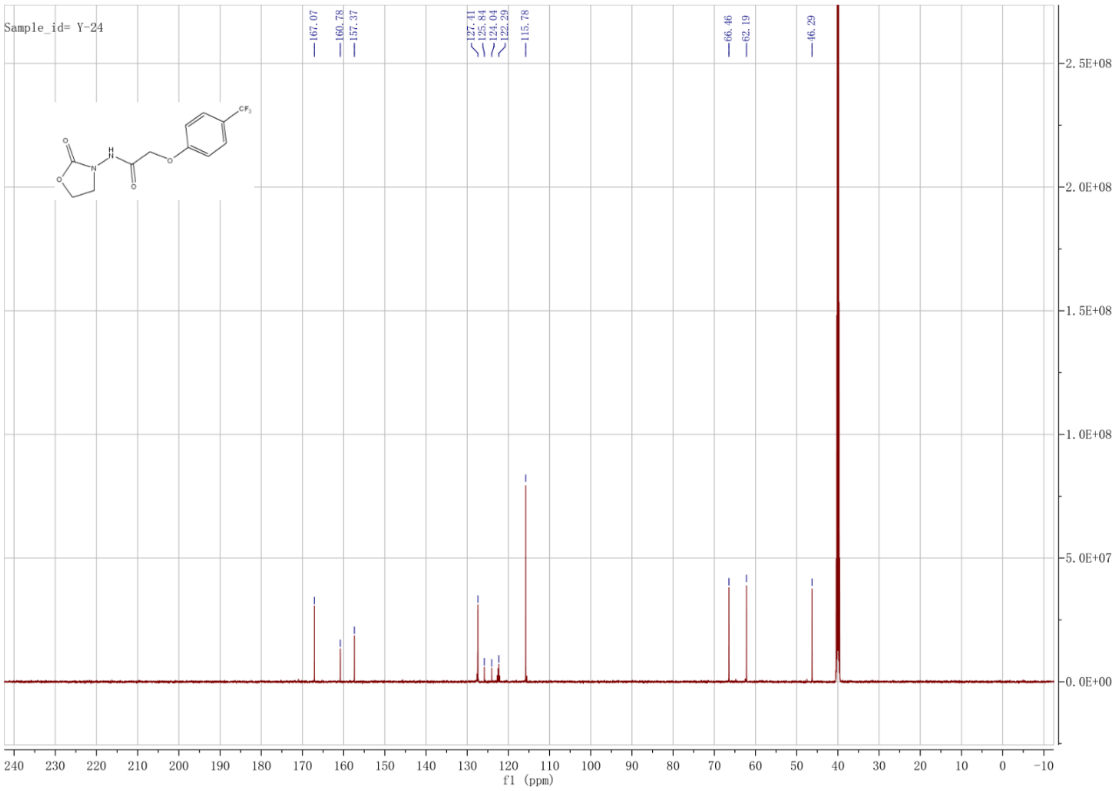

The ^{13}C NMR spectrum of compound Y-24

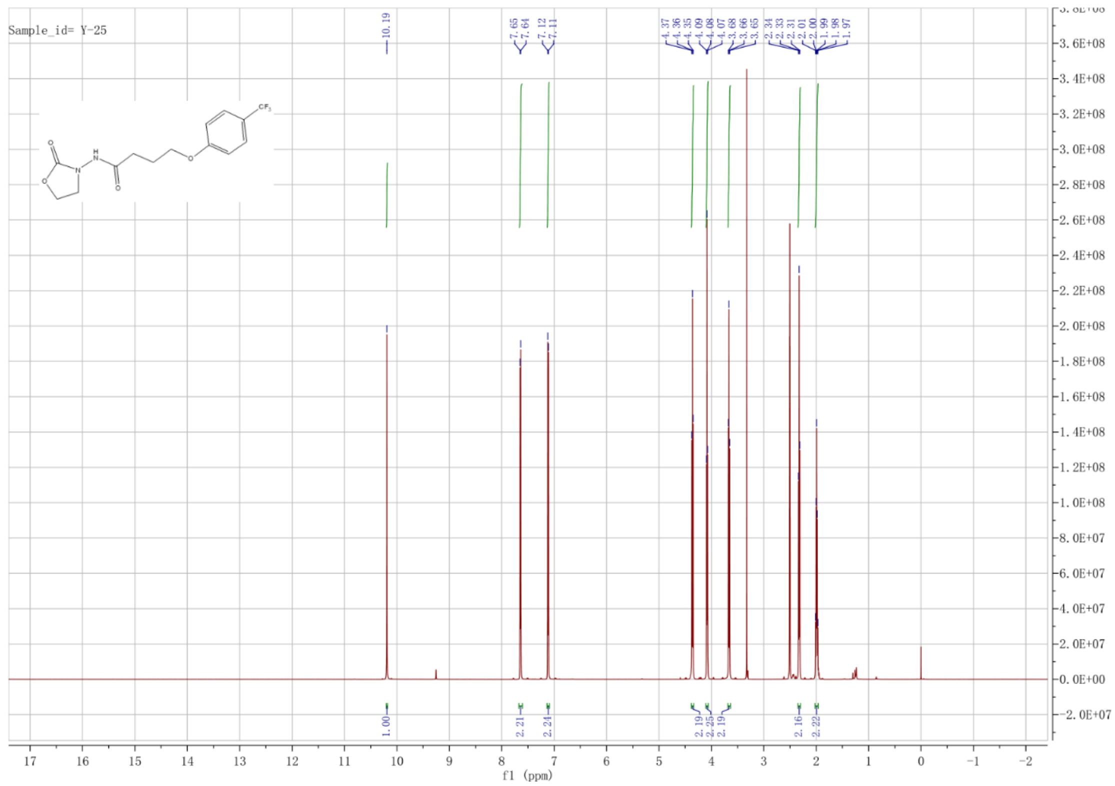

The 1H-NMR spectrum of compound Y-25

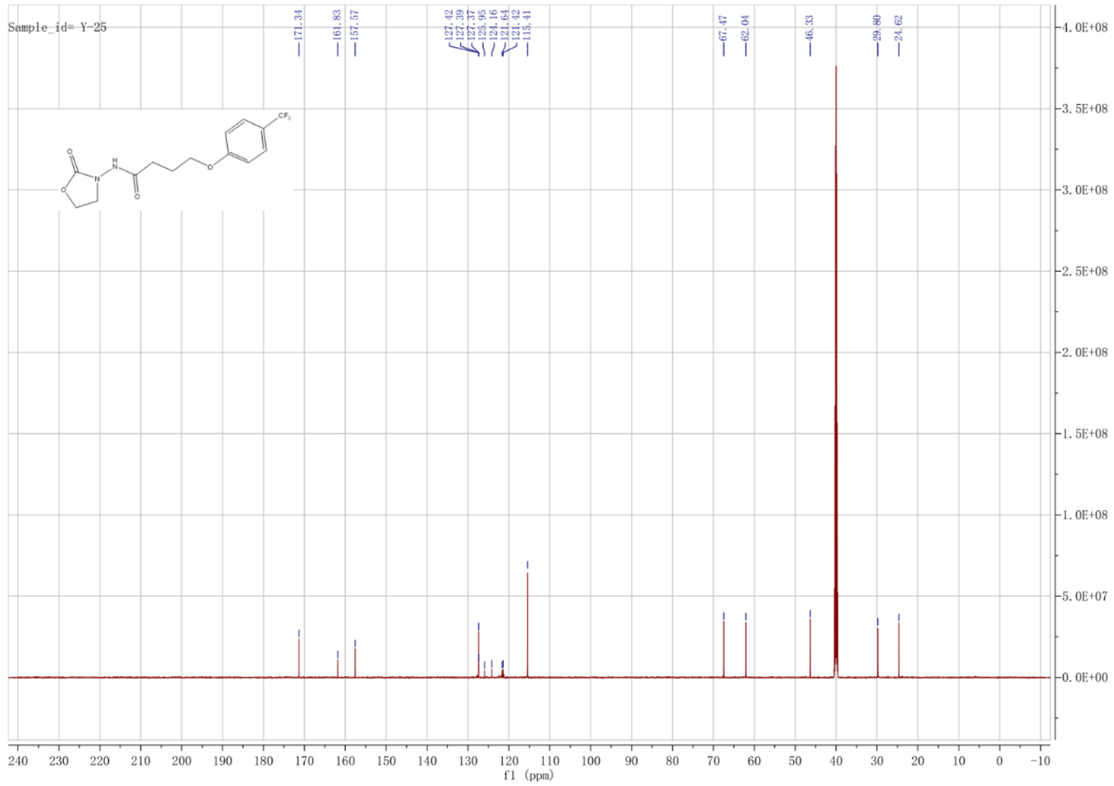

The ^{13}C NMR spectrum of compound Y-25

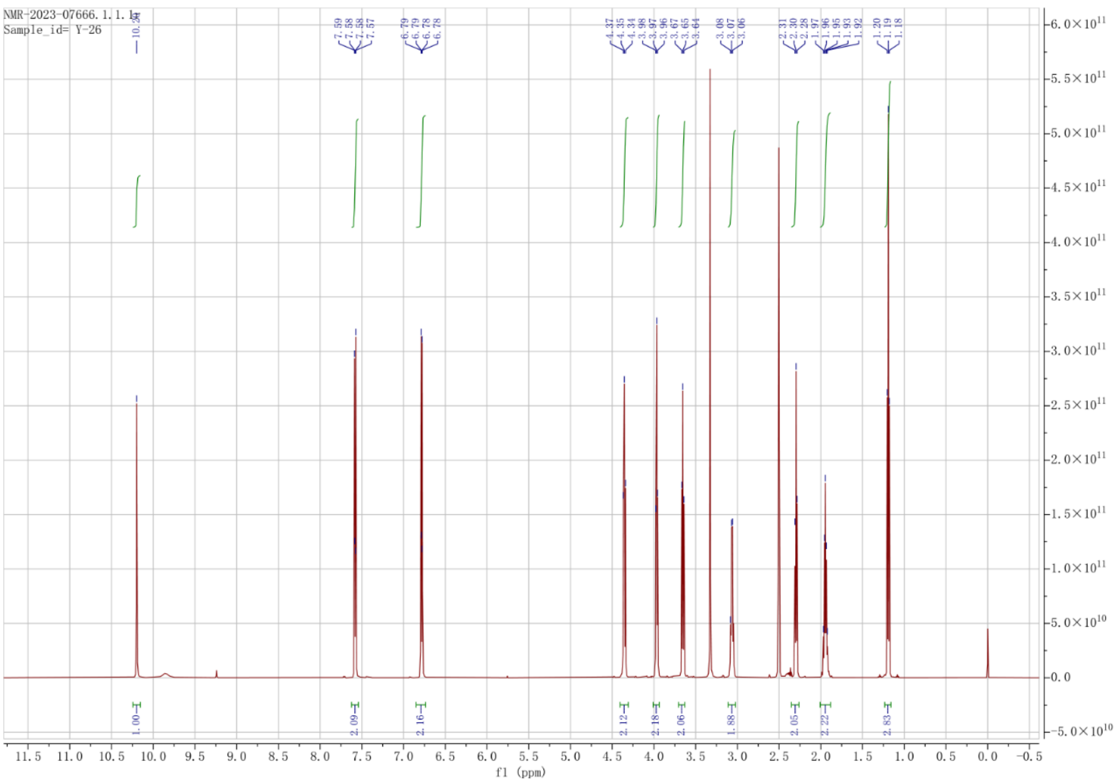

The 1H-NMR spectrum of compound Y-26

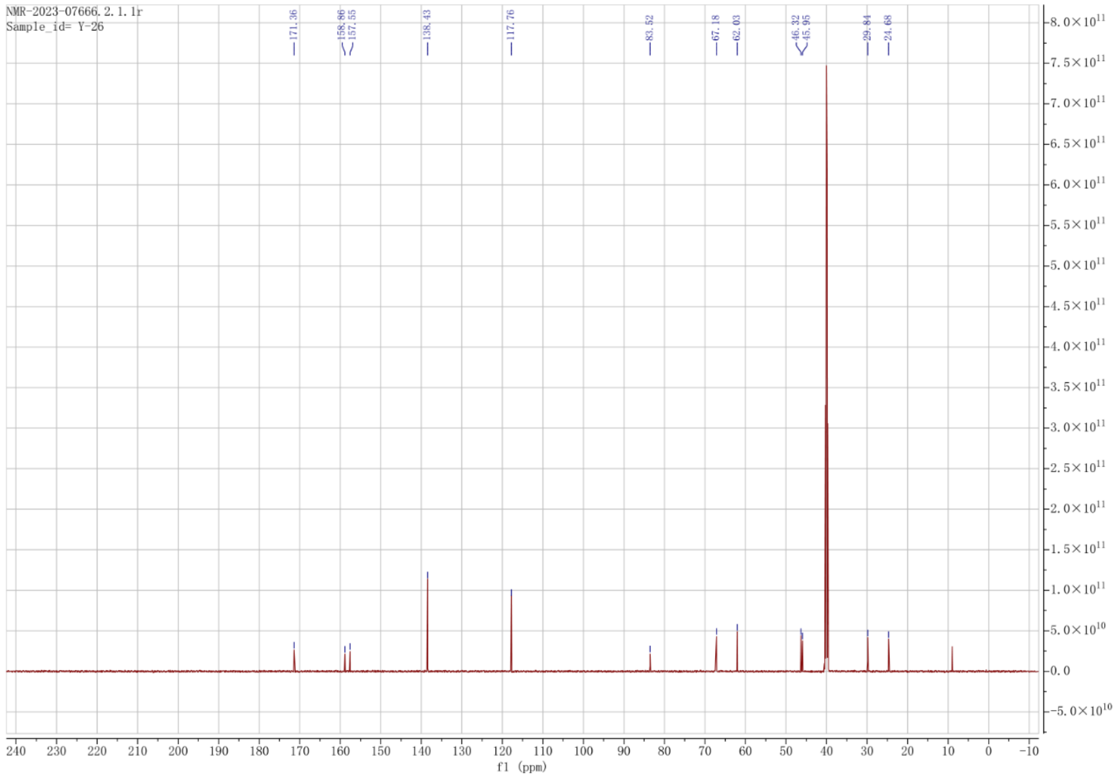

The ^{13}C NMR spectrum of compound Y-26

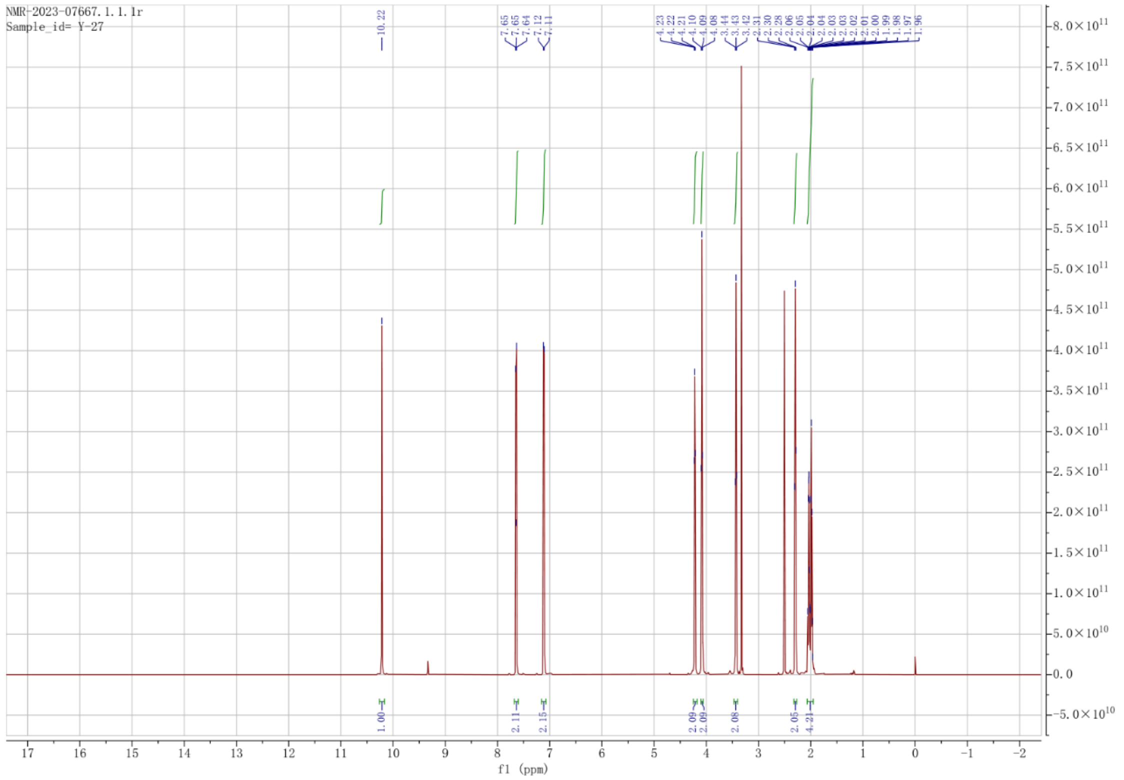

The 1H-NMR spectrum of compound Y-27

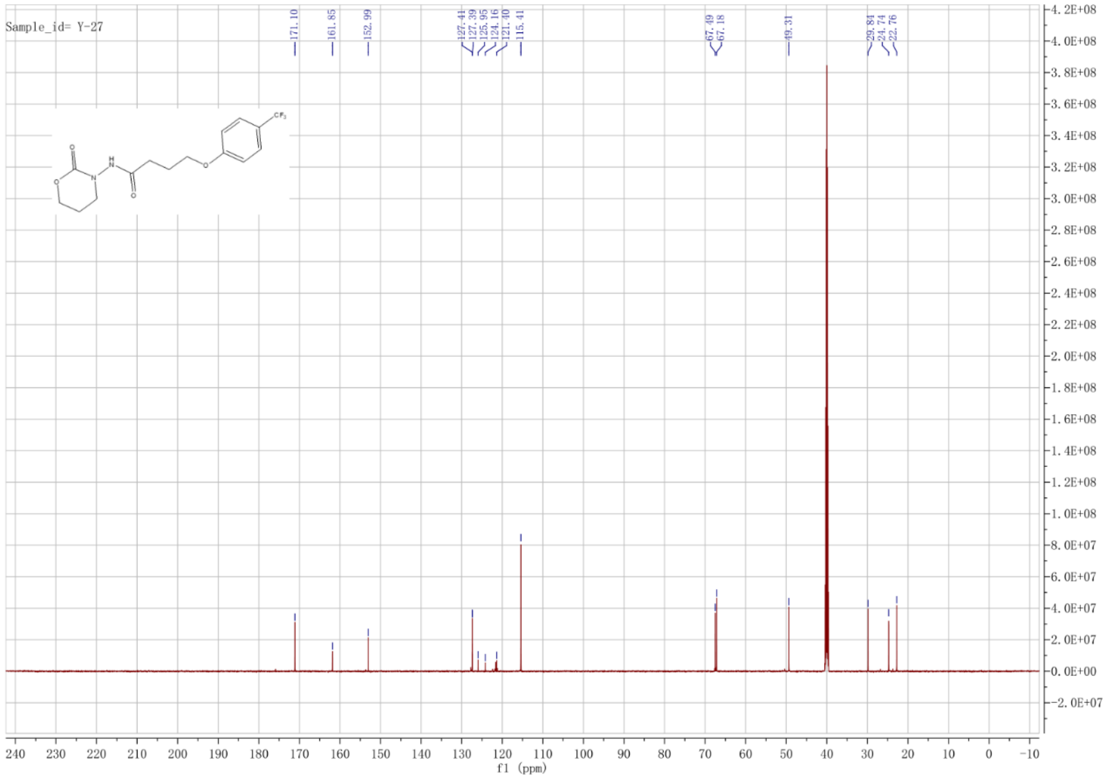

The ^{13}C NMR spectrum of compound Y-27

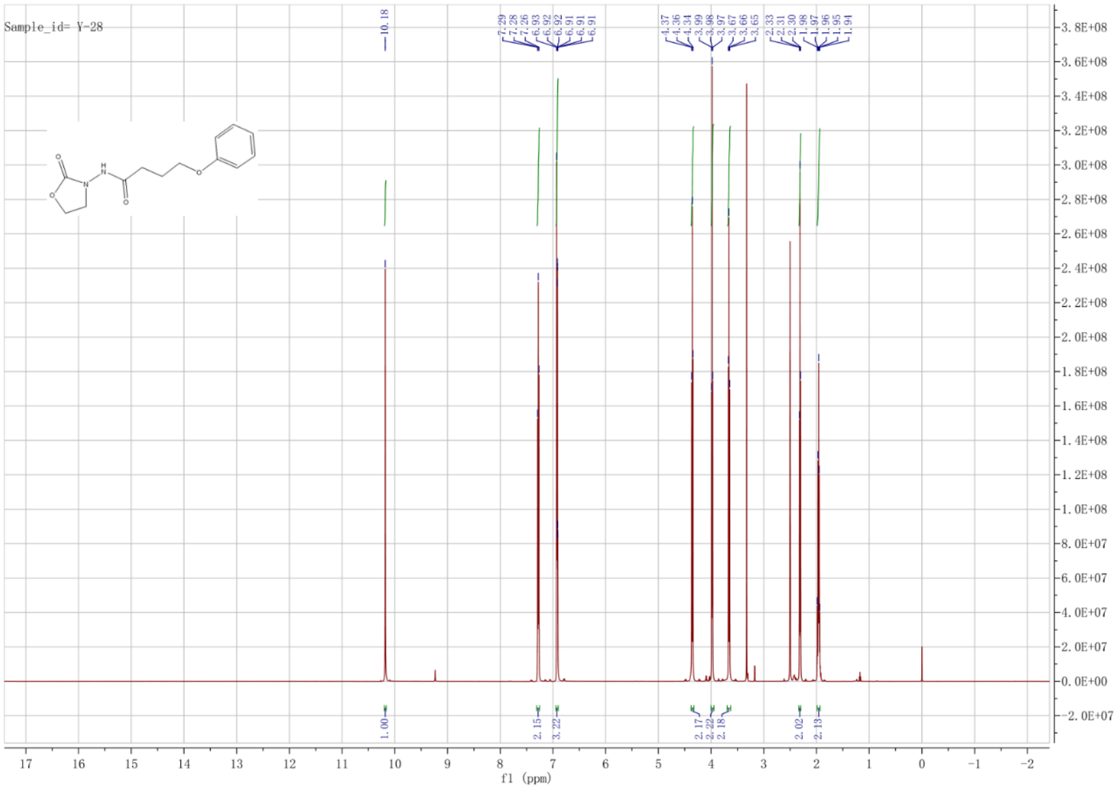

The 1H-NMR spectrum of compound Y-28

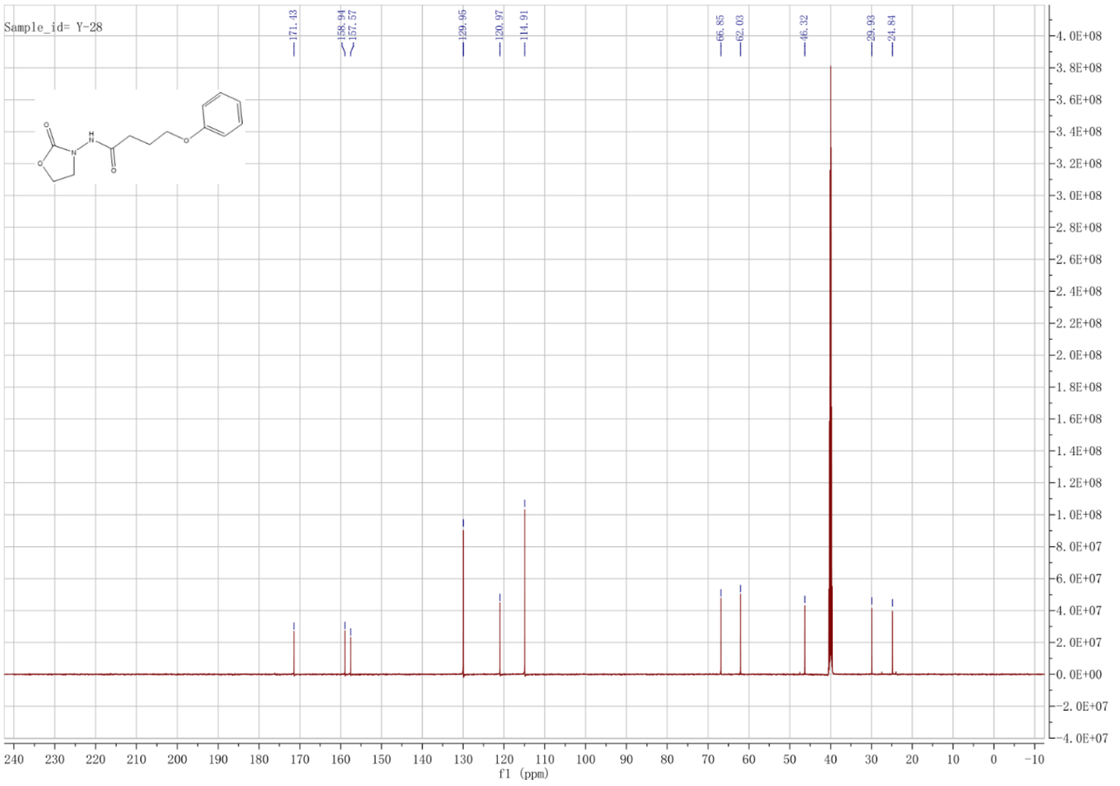

The ^{13}C NMR spectrum of compound Y-28

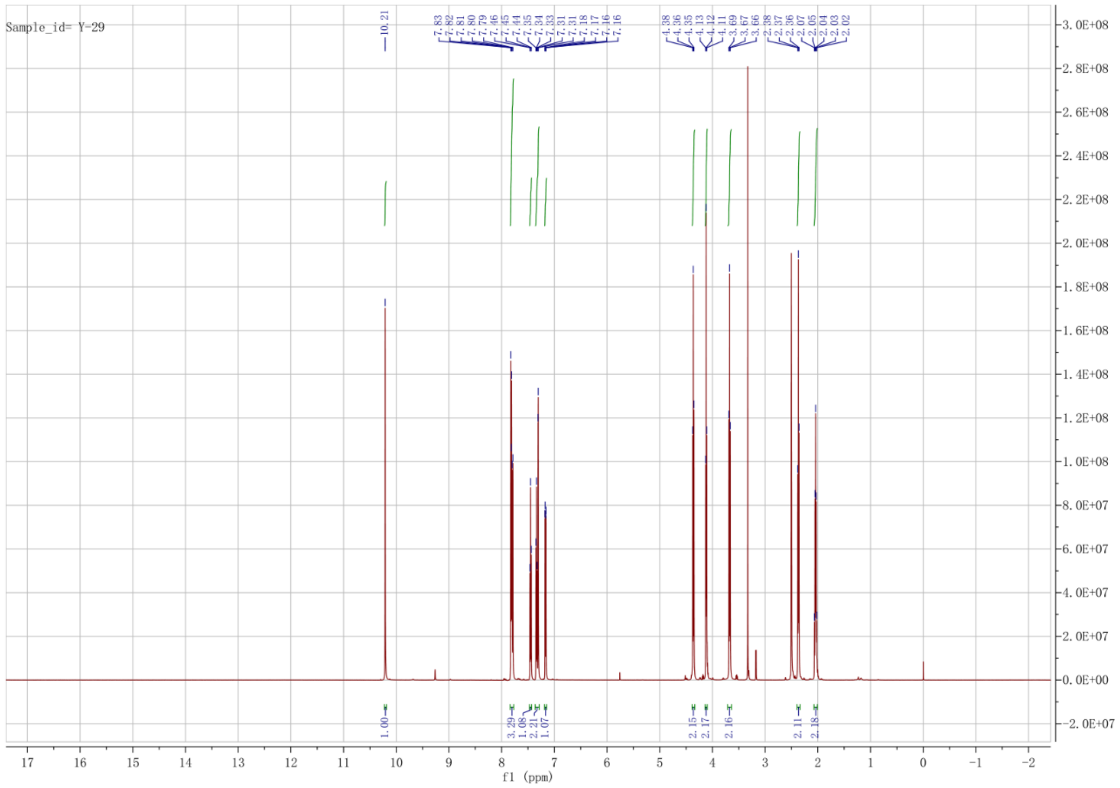

The 1H-NMR spectrum of compound Y-29

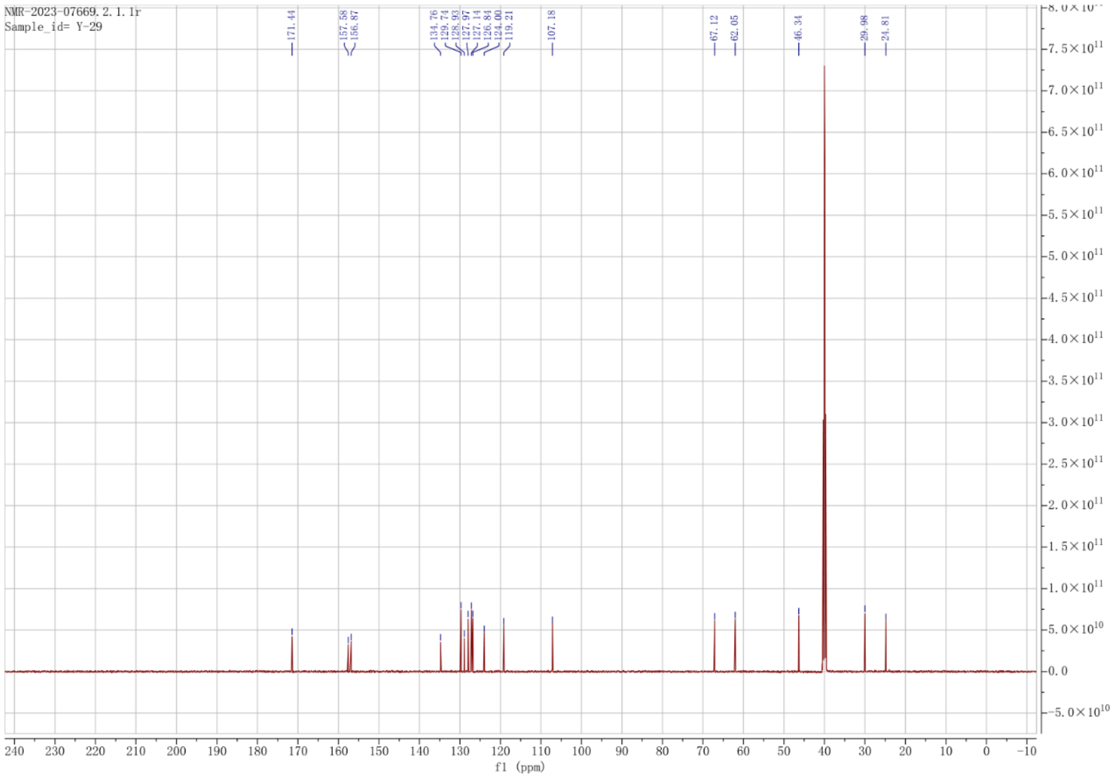

The ^{13}C NMR spectrum of compound Y-29

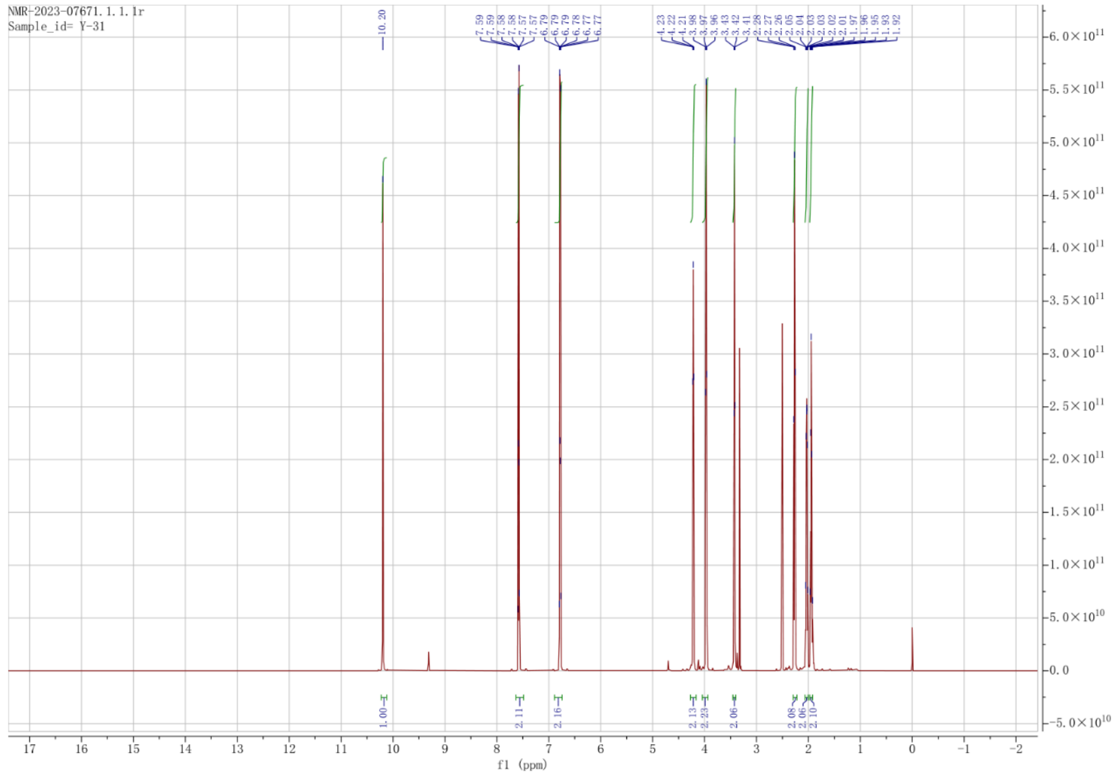

The 1H-NMR spectrum of compound Y-31

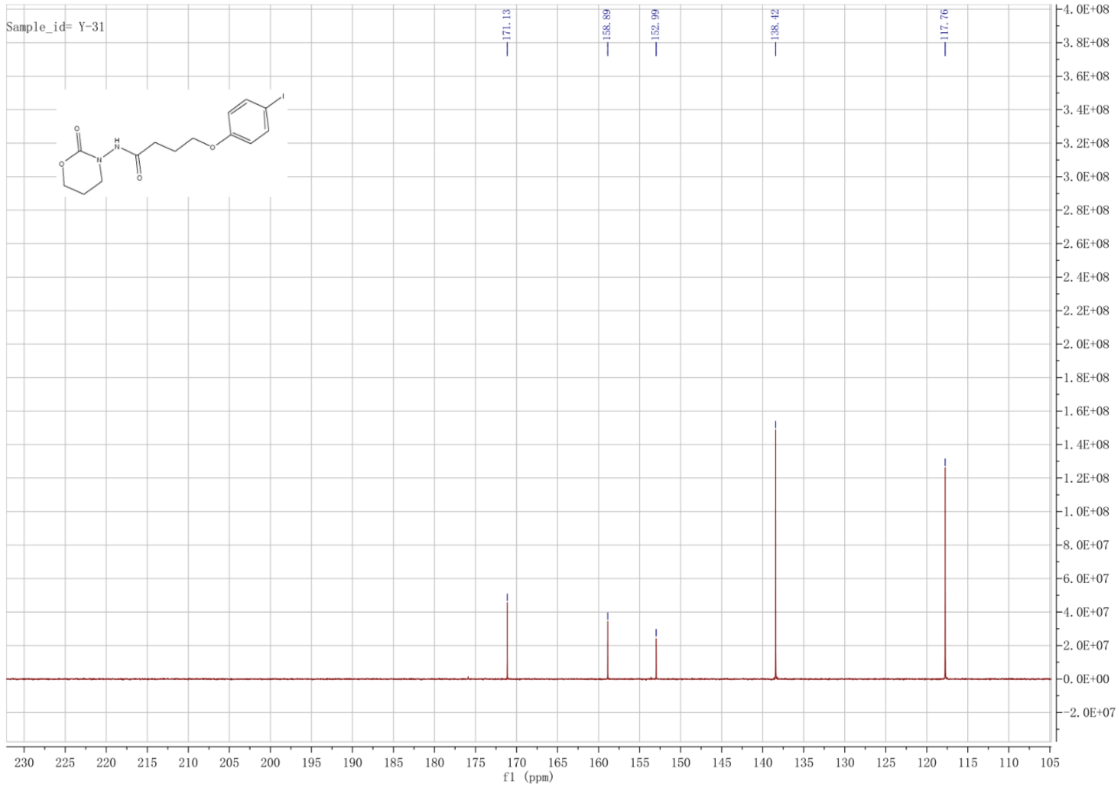

The ^{13}C NMR spectrum of compound Y-31

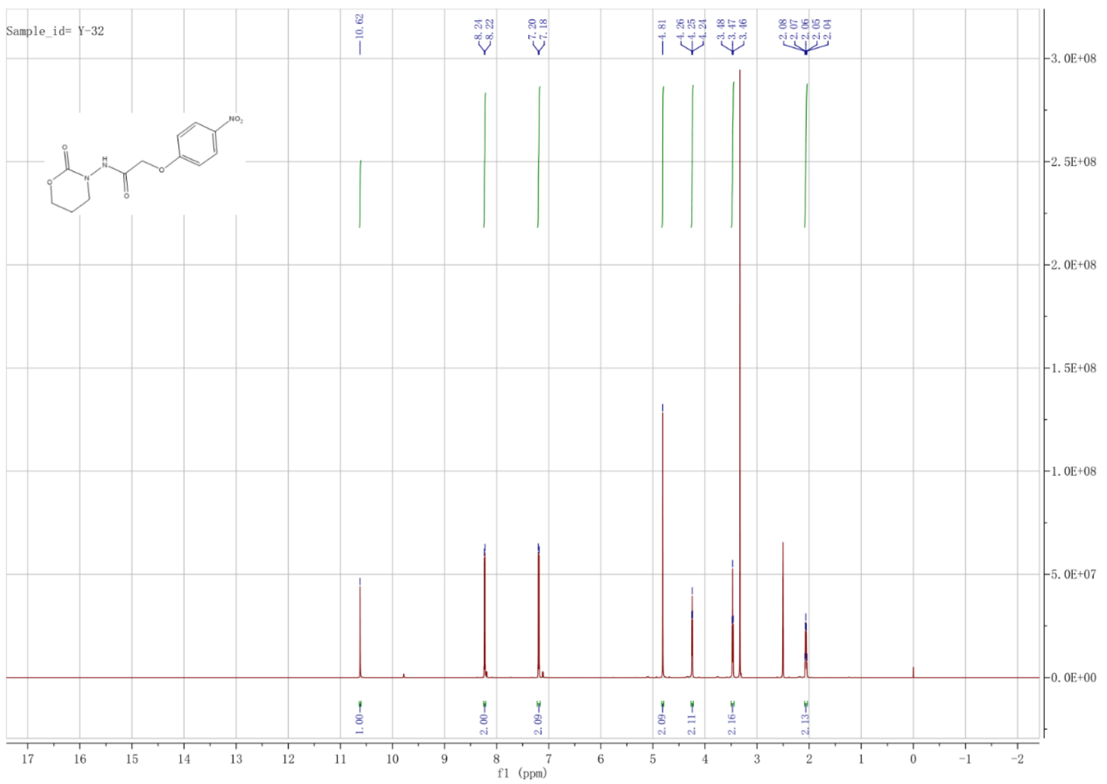

The 1H-NMR spectrum of compound Y-32

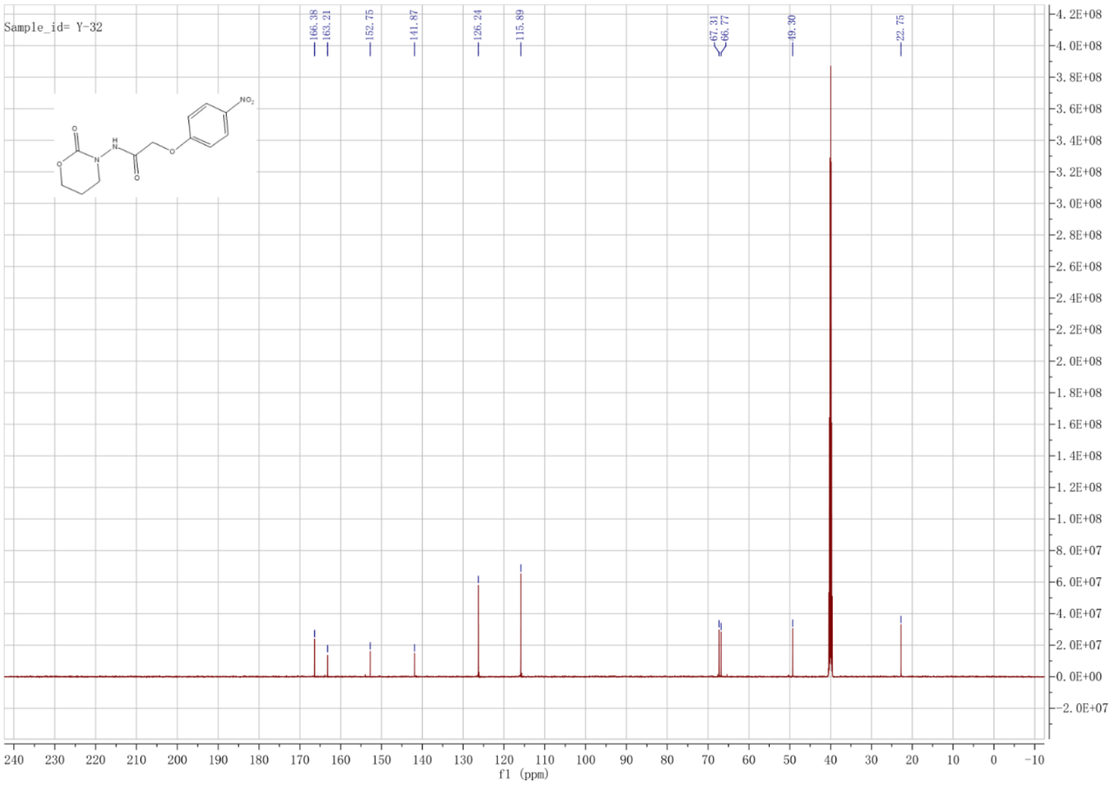

The ^{13}C NMR spectrum of compound Y-32

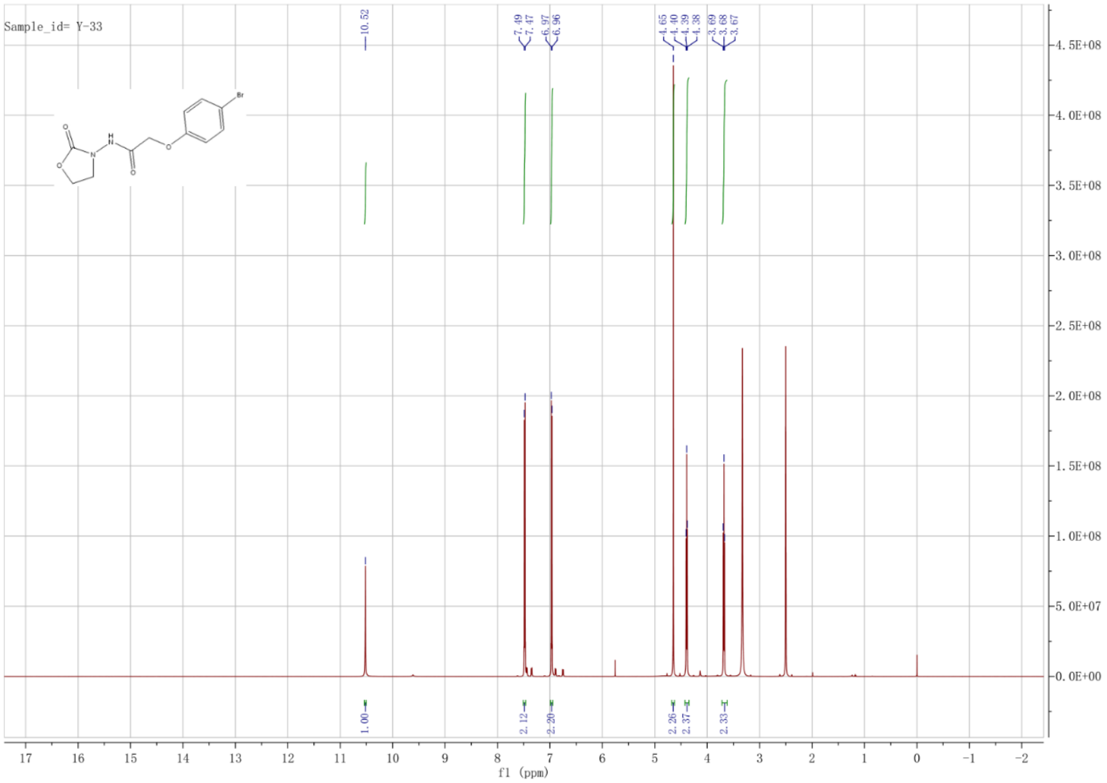

The 1H-NMR spectrum of compound Y-33

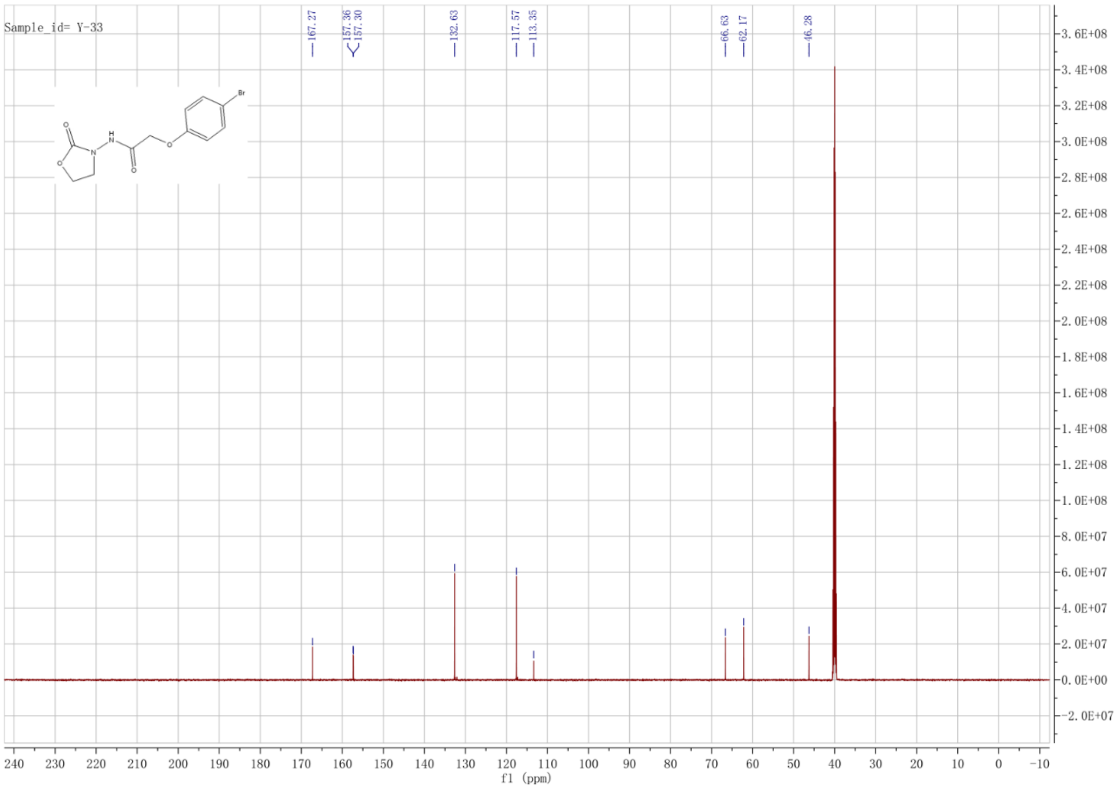

The ^{13}C NMR spectrum of compound Y-33

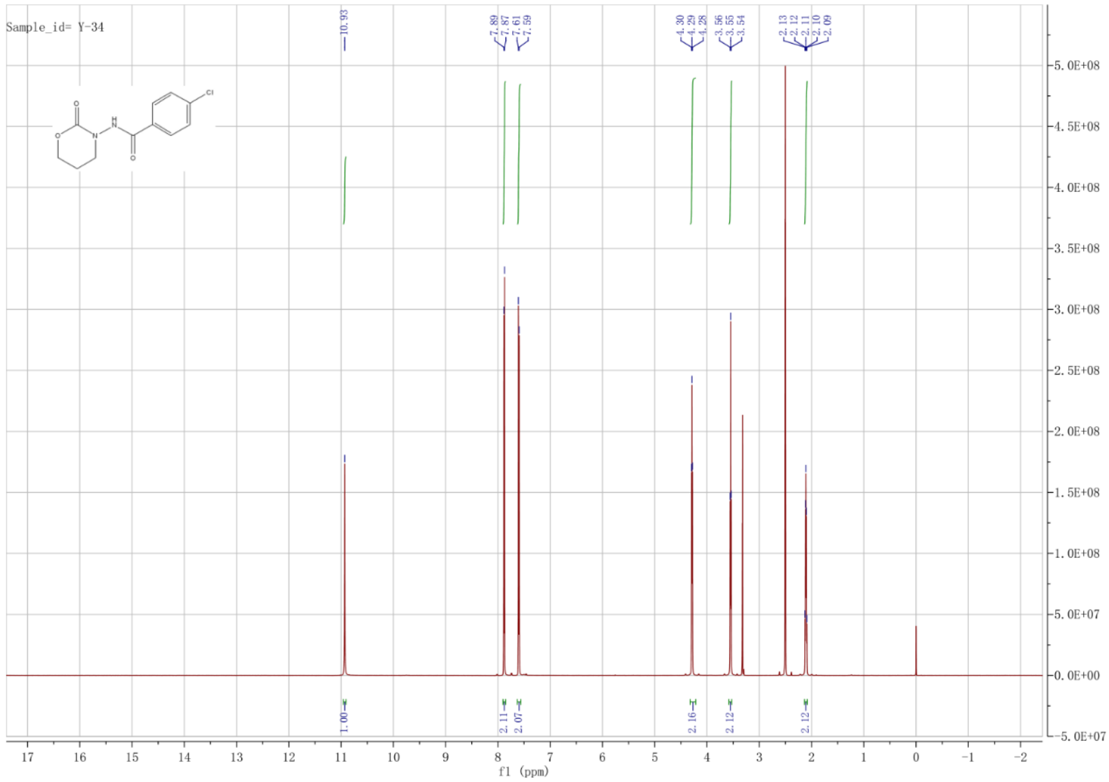

The 1H-NMR spectrum of compound Y-34

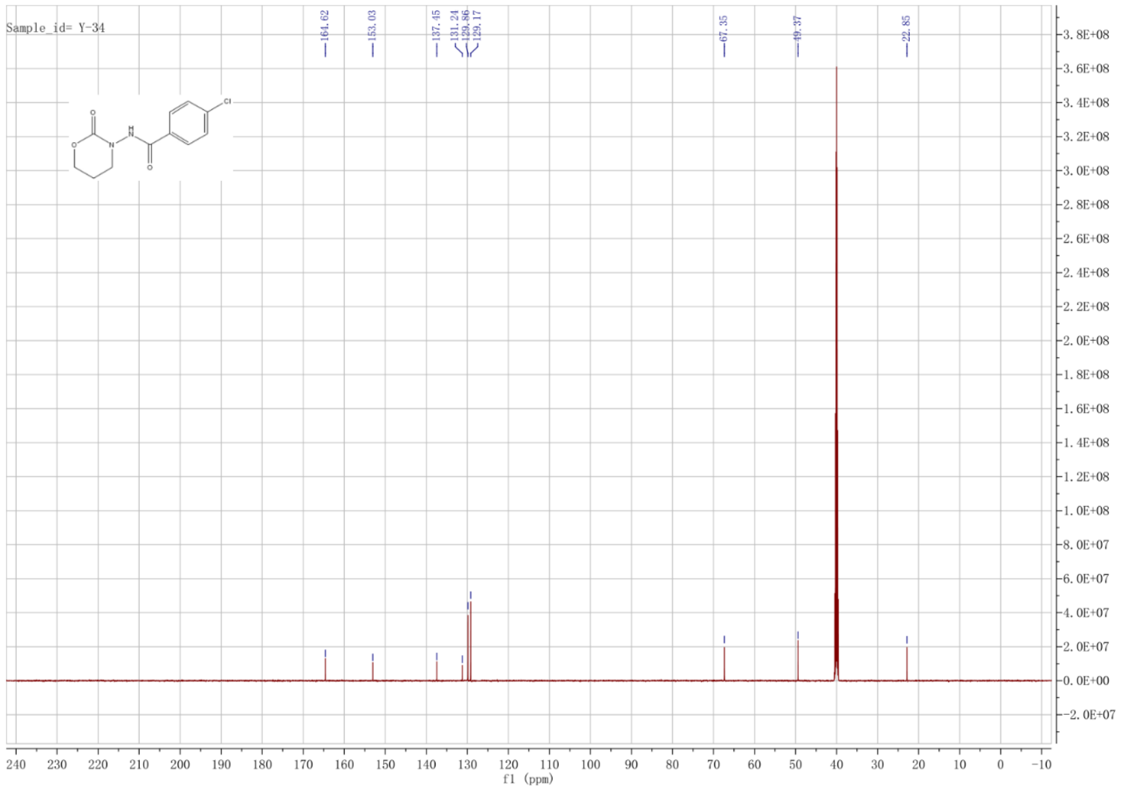

The ^{13}C NMR spectrum of compound Y-34

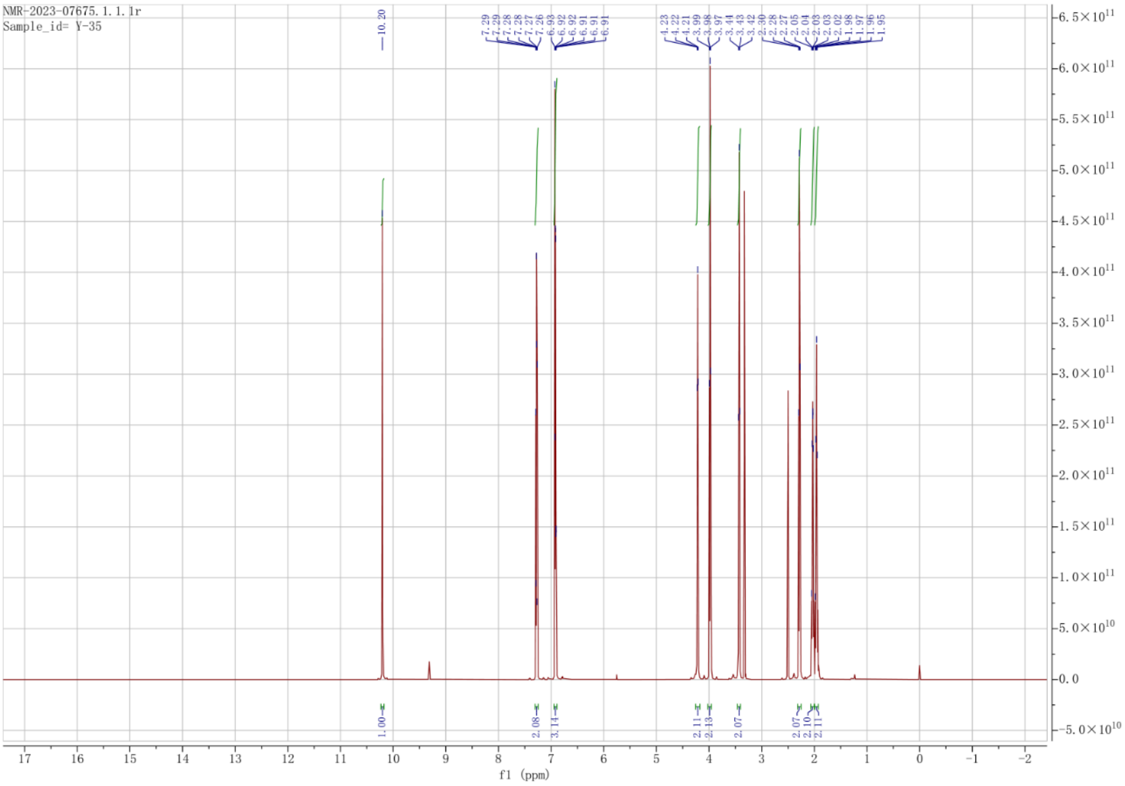

The 1H-NMR spectrum of compound Y-35

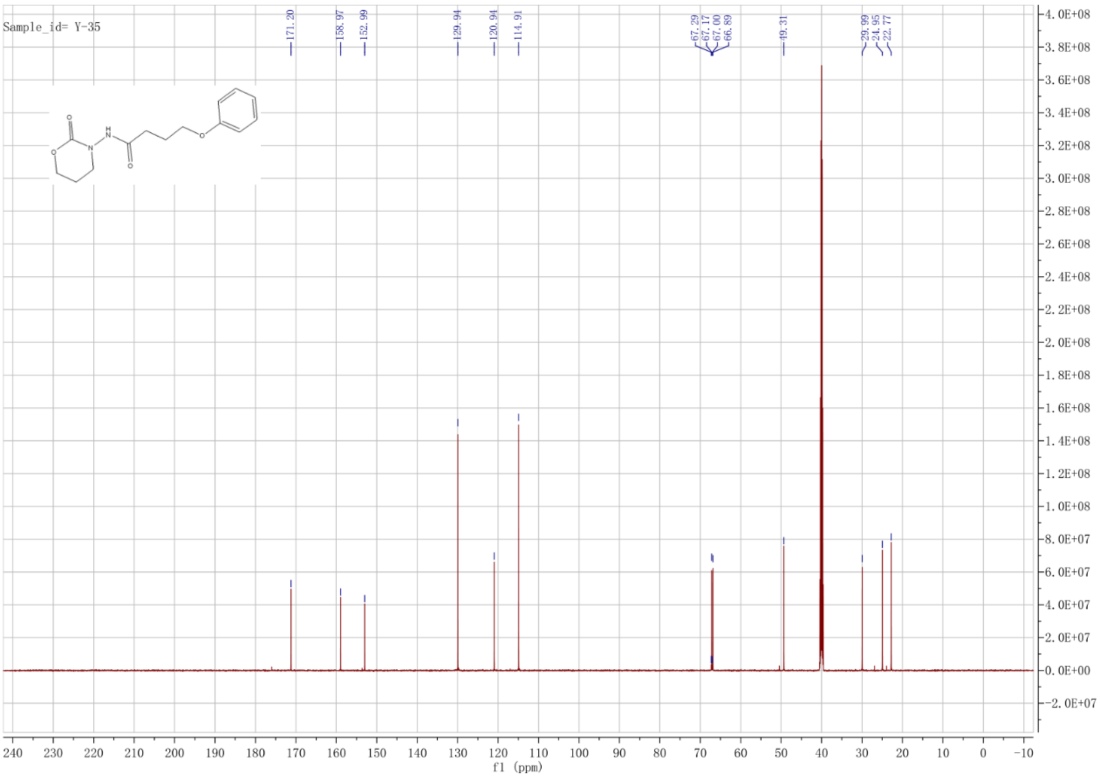

The ^{13}C NMR spectrum of compound Y-35

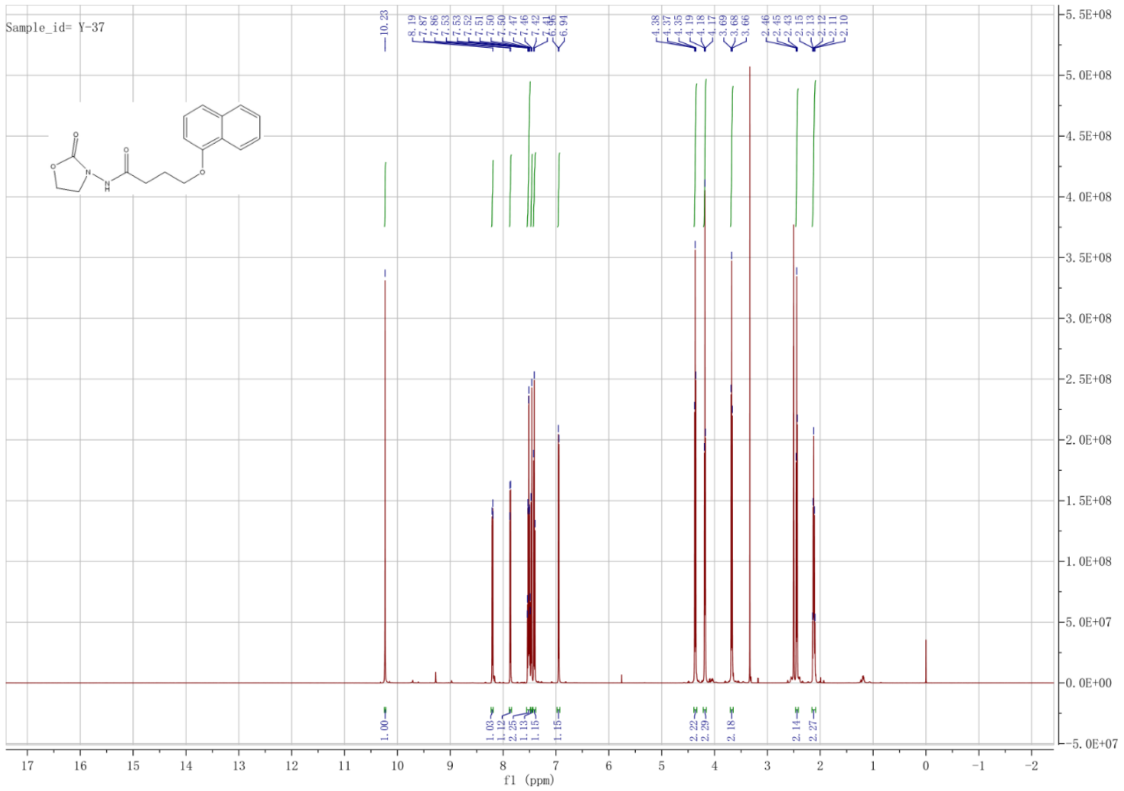

The 1H-NMR spectrum of compound Y-37

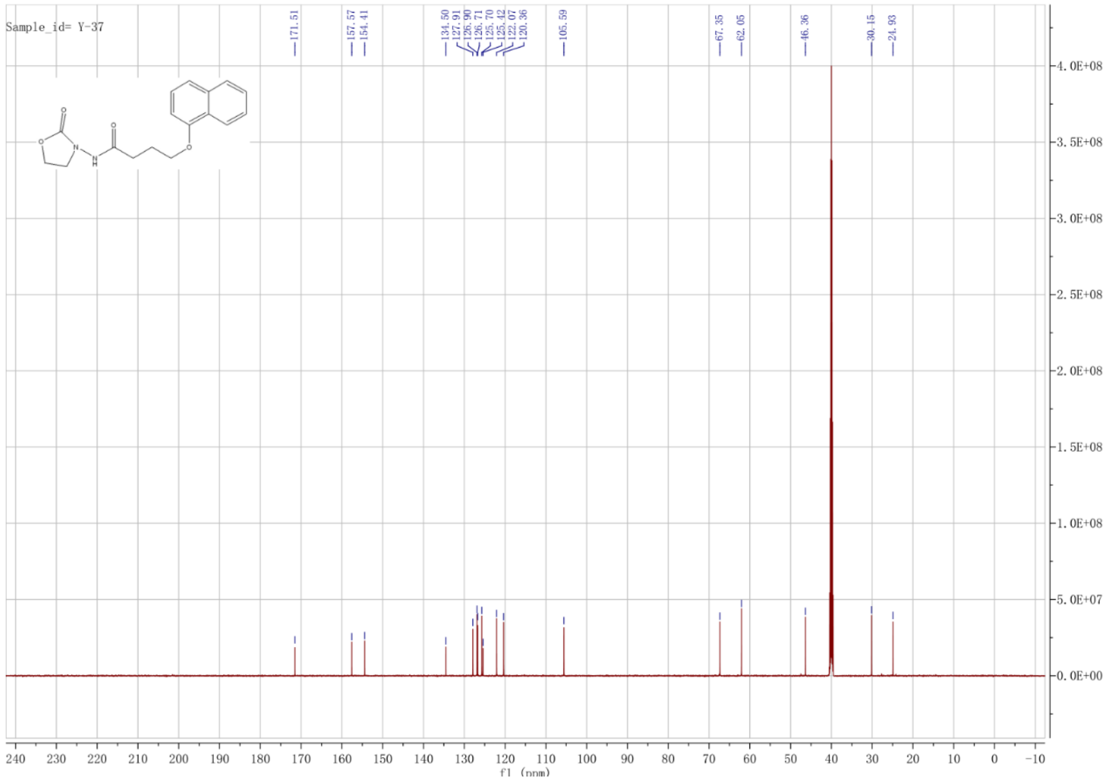

The ^{13}C NMR spectrum of compound Y-37

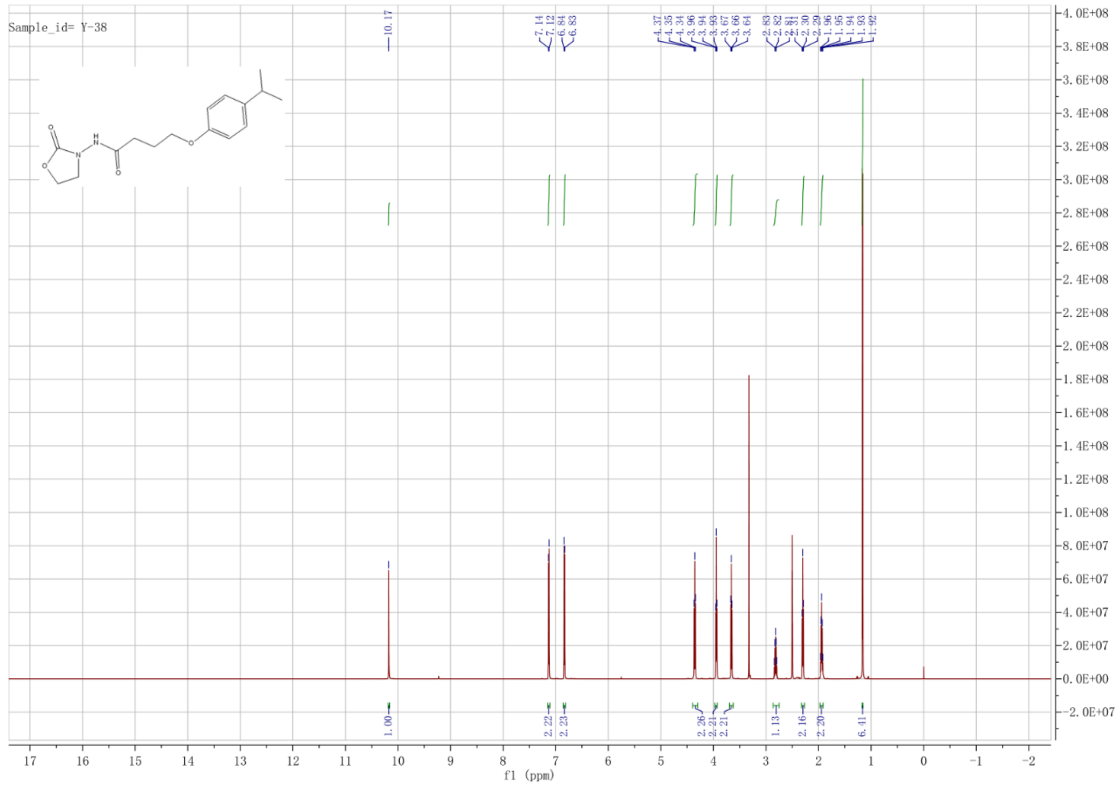

The 1H-NMR spectrum of compound Y-38

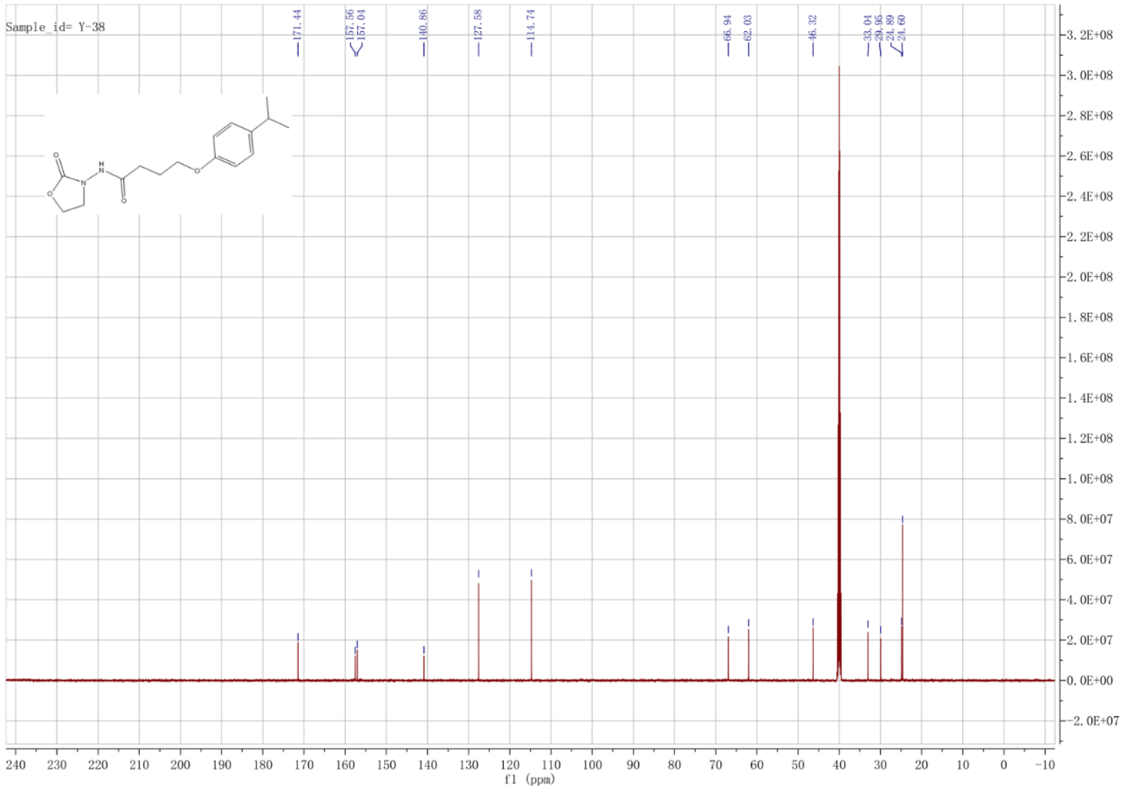

The ^{13}C NMR spectrum of compound Y-38

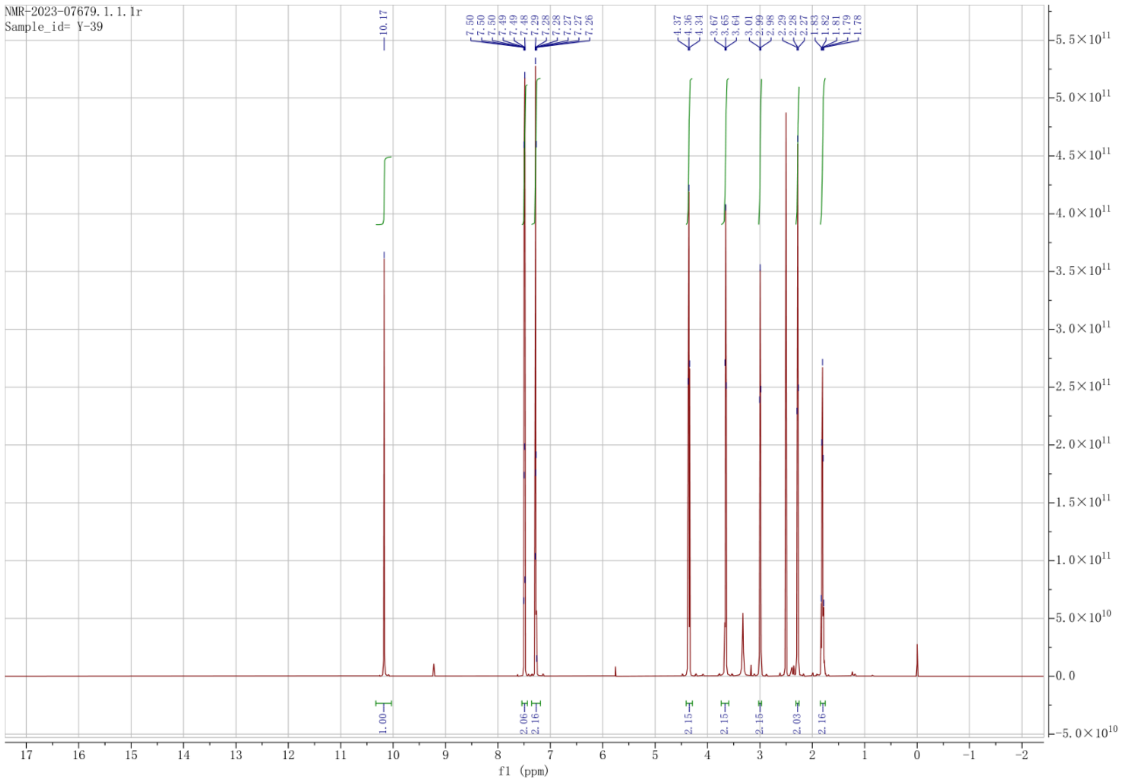

The 1H-NMR spectrum of compound Y-39

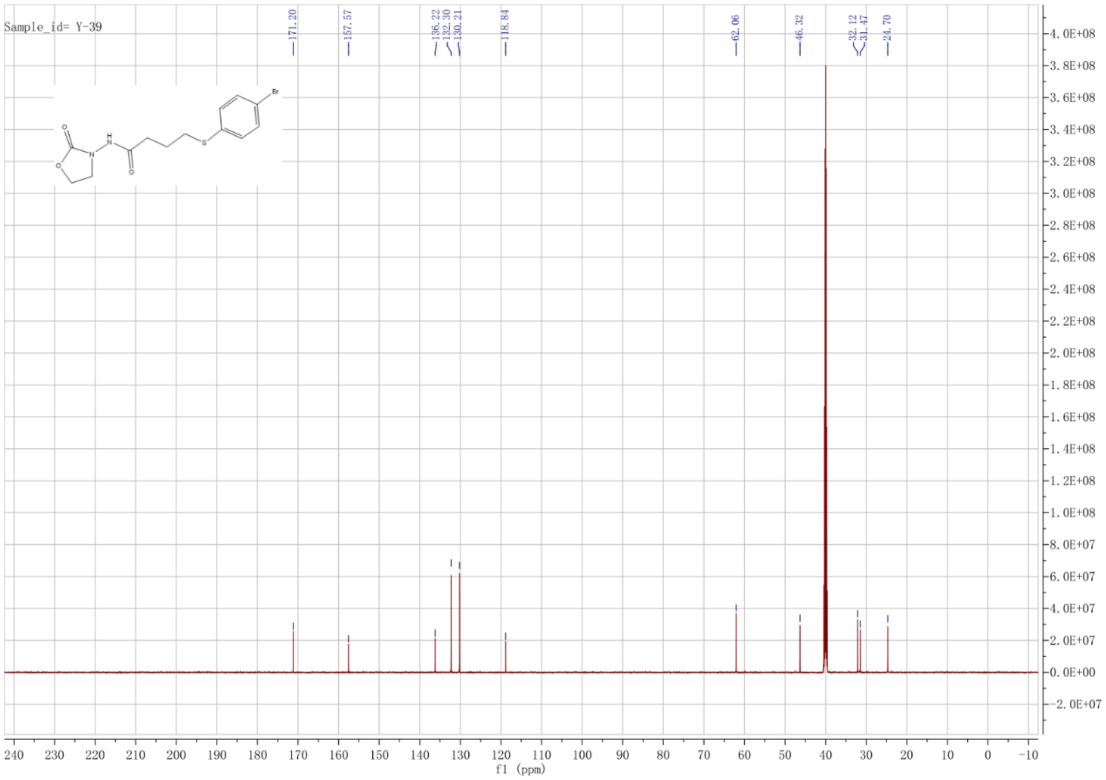

The ^{13}C NMR spectrum of compound Y-39

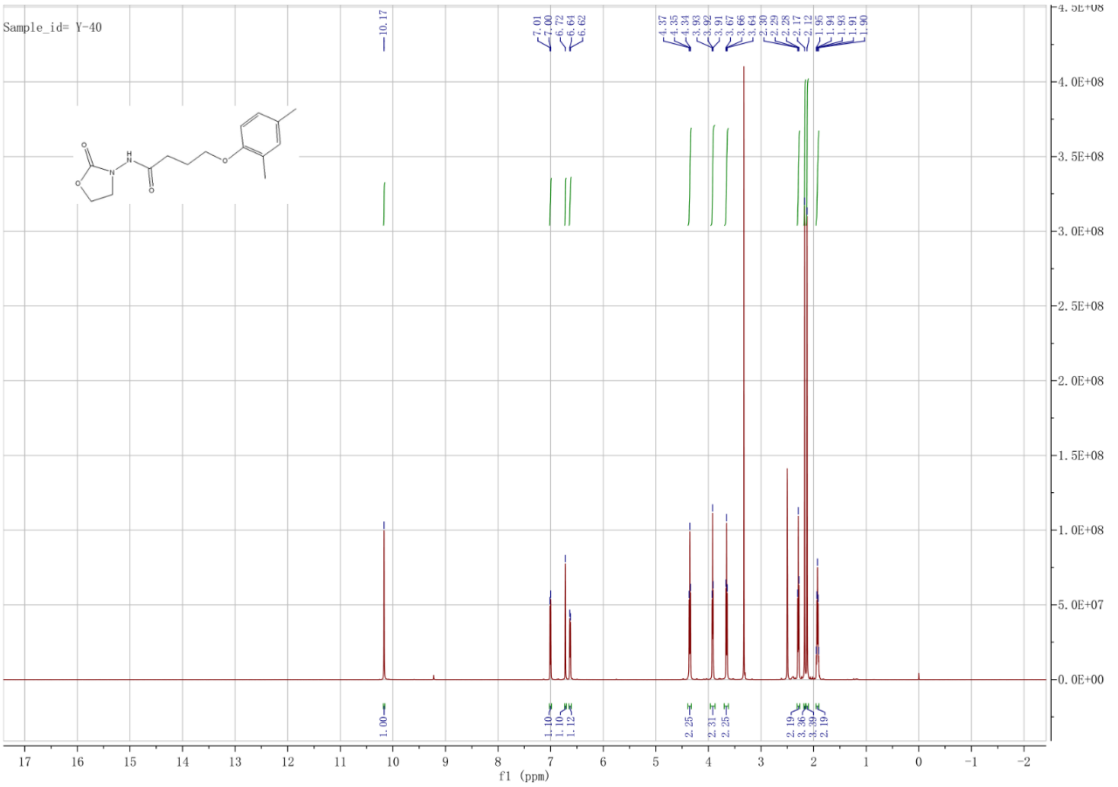

The 1H-NMR spectrum of compound Y-40

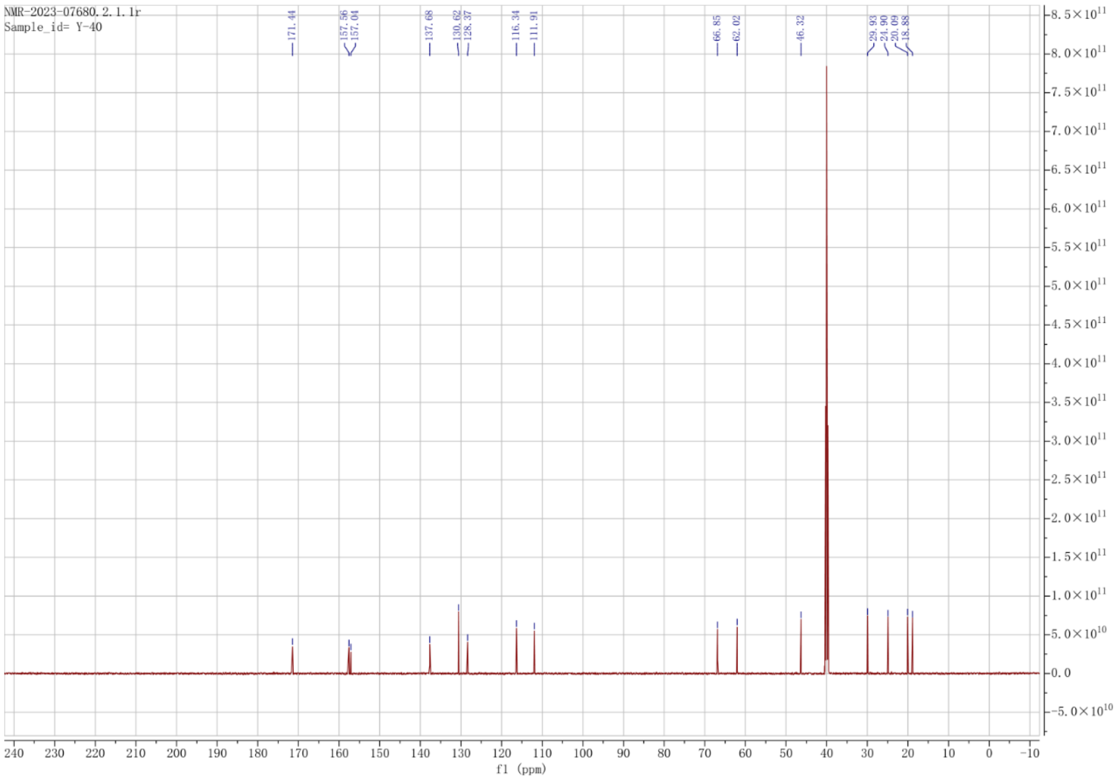

The ^{13}C NMR spectrum of compound Y-40

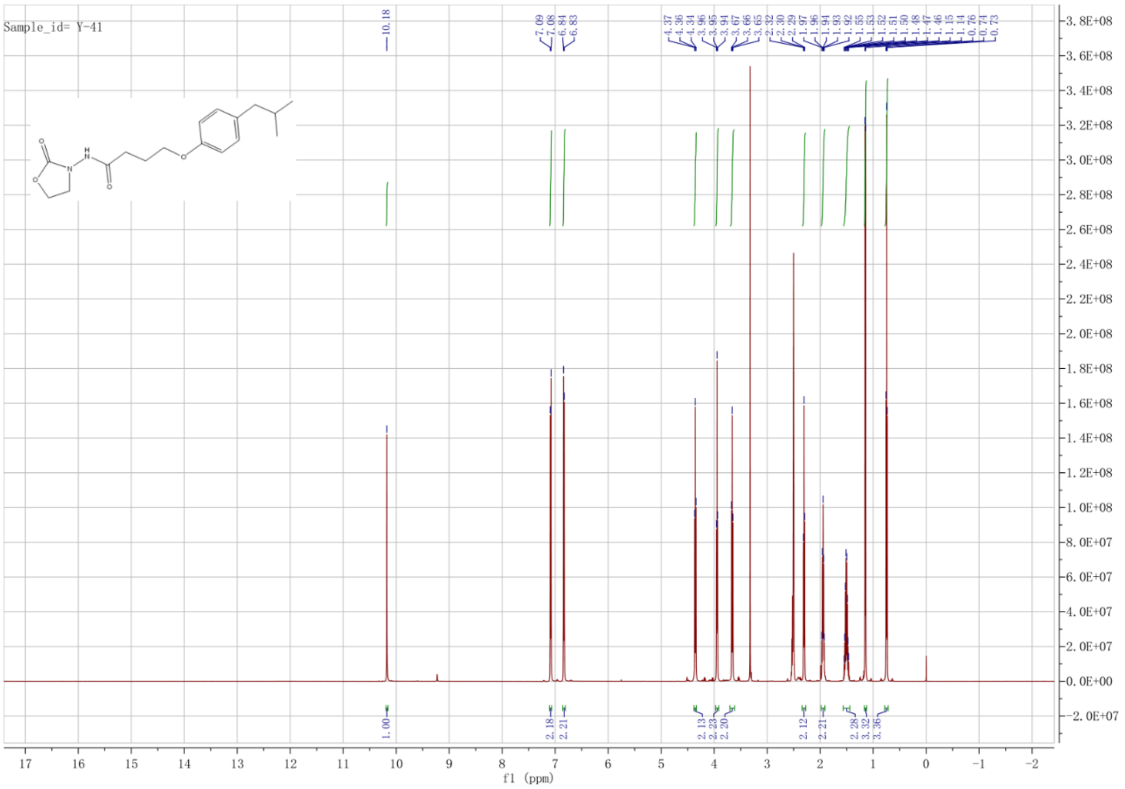

The 1H-NMR spectrum of compound Y-41

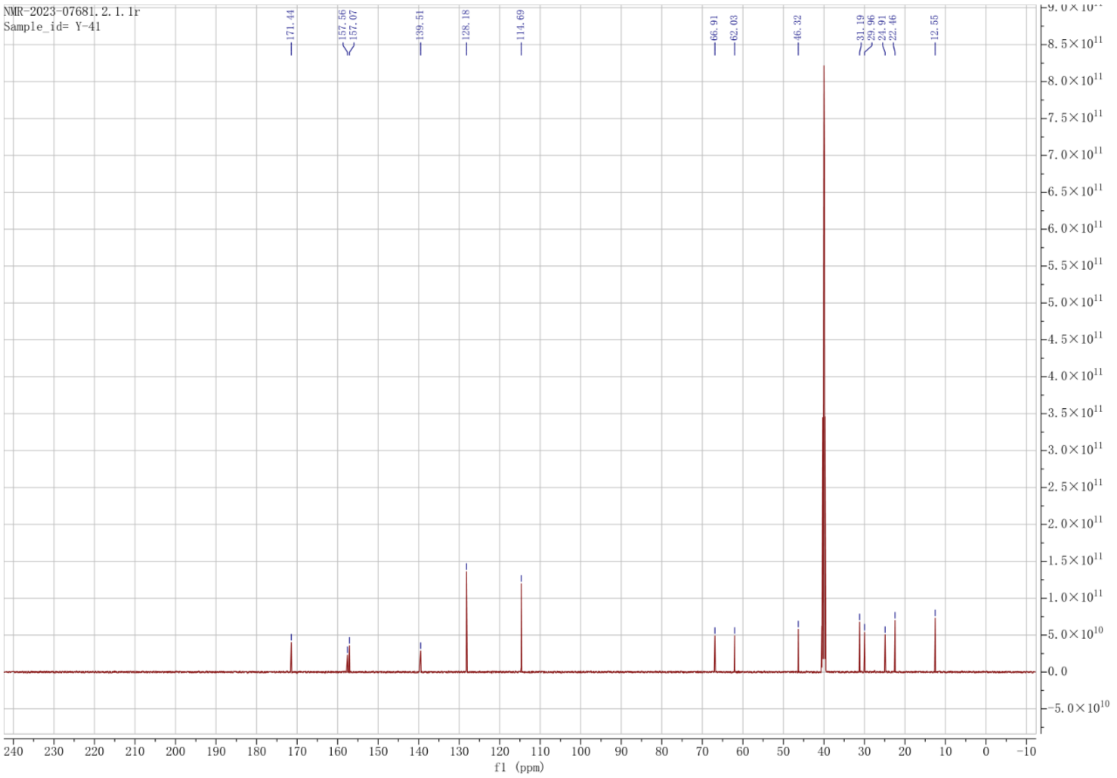

The ^{13}C NMR spectrum of compound Y-41
