# Supplementary material for: Design, Synthesis, and Biological Evaluation of the Quorum-Sensing Inhibitors of Pseudomonas aeruginosa PAO1
Source: Molecules. 2024 May 8;29(10):2211. doi: 10.3390/molecules29102211 (PMC11123961; doi:10.3390/molecules29102211)
Supplement: Supplementary file 1 [file molecules-29-02211-s001.zip › Supplementary material for experimental procedures (1).pdf]

Supplemental information

Design, synthesis, and biological evaluation of the quorum-sensing inhibitors of *Pseudomonas aeruginosa* PAO1

1 National Engineering Research Center for the Emergency Drug, State Key Laboratory of Toxicology and Medical Countermeasures, Beijing Institute of Pharmacology and Toxicology, Beijing 100850, China; yanxinlin11@163.com (X.Y.) houshi28@126.com (S.H.); jiajiachang@126.com (J.C.);

2 School of Pharmaceutical Sciences, Jilin University, Changchun 130021, China; xing-cheng21@mails.jlu.edu.cn (C.X.)

3 School of Life Sciences, Jilin University, Changchun, 130012, China; yuanyuanz21@mails.jlu.edu.cn (Y.Z)

* Correspondence: xiaojunhai@139.com (J.X); Tel: 01066931634

3.1.1. Synthesis of 3-amino-2-oxazolidinone

First, a 250 mL three-neck container was used to hold 38.01 g of 2-hydrazine ethanol (0.50 mol), 78.39 g of diethyl carbonate (0.65 mol), and 7.50 g of sodium methoxide (0.15 mol). For four hours, the solution was heated under reflux. The solid precipitate was filtered, and the solids were dried after cooling to room temperature. After drying, the solvent was evacuated. The products underwent column chromatography (methanol/dichloromethane *V*/*V* 1:40). Ethanol was used to recrystallize the dry solid and white solid. With a weight of 39.58 g, the white solid yielded 77.61%. ESI-MS *m*/*z*: 103.0506 [M + H].

3.1.2. Synthesis of Target Compound **Y-1**

Synthesis of Ethyl 4-(Isoquinolin-7-yloxy) Butanoate

A 100 mL three-neck bottle contained ethyl 4-bromobutyrate (1 g, 5.1 mmol), 7-Hydroxyisoquinoline (0.74 g, 5.1 mmol), and 50 mL of acetonitrile. As cesium carbonate (3.32 g, 10.2 mmol) was added and the mixture was allowed to dissolve, the temperature of the solution rose to 50 °C. After 8 h of stirring, the mixture was allowed to cool to ambient temperature, vacuum-filtered, and then dried using a rotary evaporator. The residue was diluted with 50 mL of water, and then the solution was extracted using ethyl acetate (50 mL × 3). Anhydrous sodium sulfate was mixed with the organic layer and allowed to dry overnight. Vacuum filtering was followed by reduced pressure evaporation of the solvent to provide a crude product (light yellow oil 1.2 g, yield 91.01%) that was used straight away for the next stage without additional purification. ESI-MS *m*/*z*: 260.12 $[M + H]^+$.

Synthesis of 4-(Isoquinolin-7-yloxy) Butanoic Acid

In a 100 mL tri-necked bottle containing ethyl 4-(isoquinolin-7-yloxy) butanoate (1.2 g, 4.63 mmol), 20 mL of ethanol was added, and the mixture was shaken until it dissolved. Then, 3 mL of water and NaOH (0.75 g, 18.8 mmol) were added to the bottle at 25 °C, and the solution was refluxed for 2 h. Through the use of thin-layer chromatography (TLC), the reaction was observed. After cooling the mixture to 25 °C, it was dried with a rotary evaporator, and the remaining 30 mL was diluted with water. Then, 1N hydrochloric acid was added to the solution to bring its pH to 5, resulting in an amount of solid white precipitate. After vacuum filtration was employed to dry the precipitate, a crude product (white solid 0.86 g, yield 80.8%) was obtained. This product was used straight away for the following stage without undergoing additional purification. ESI-MS *m*/*z*: 232.09 $[M + H]^+$.

Synthesis of 4-(4-Fluorophenoxy-N-(2-oxazolidinone)-3-yl) Butylamide (**Y-1**)

By adding 4-(4-fluorophenoxy) butyric acid (0.86 g, 3.72 mmol) and 20 mL of dichloromethane in a 50 mL tri-necked container, the mixture was swirled until dissolved. Then, EDCI (0.85 g, 4.44 mmol), HOBt (0.6 g, 4.44 mmol), and triethylamine (0.84 g, 9.25 mmol) were added to a bottle at 0 °C. After agitating in an ice bath for 1 h, 3-amino-2-oxazolidinone (0.37 g, 3.72 mmol) was added again. After heating the mixture to 25 °C, it was stirred overnight. Following TLC, the product underwent rotational evaporation drying after being vacuum-filtered. Column chromatography (CH_2Cl_2: MeOH = 40:1). White solid, 0.77 g. Yield, 66.33%. ^1H NMR (600 MHz, DMSO-d_6) δ 10.23 (s, 1H), 8.82 (d, *J* = 4.6 Hz, 1H), 8.27 (d, *J* = 8.1 Hz, 1H), 7.89 (d, *J* = 8.9 Hz, 1H), 7.40–7.35 (m, 2H), 7.27 (dd, *J* = 8.9, 2.6 Hz, 1H), 4.37 (t, *J* = 7.8 Hz, 2H), 4.18 (t, *J* = 6.4 Hz, 2H), 3.68 (t, *J* = 7.8 Hz, 2H), 2.38 (t, *J* = 7.3 Hz, 2H), 2.06 (p, *J* = 6.8 Hz, 2H).^{13}C NMR (151 MHz, DMSO-d_6) δ 171.41, 159.80, 157.58, 151.11, 149.89, 136.08, 129.69, 123.53, 119.91, 119.70, 108.40, 67.37, 62.05, 46.34, 29.97, 24.71; ESI-MS *m*/*z*: 316.1292 $[M + H]^+$.

3.1.3. Synthesis of Target Compound **Y-2**

Synthesis of Ethyl 4-((6-Bromonaphthalen-2-yl) oxy) Butanoate

A 100 mL three-neck bottle contained ethyl 4-bromobutyrate (1 g, 5.1 mmol), 6-bromo-2-naphthol (1.13 g, 5.1 mmol), and 50 mL of acetonitrile. As cesium carbonate (3.32 g, 10.2 mmol) was added and the mixture was allowed to dissolve, the temperature of the solution rose to 50 °C. After 8 h of stirring, the mixture was allowed to cool to ambient temperature, vacuum-filtered, and then dried using a rotary evaporator. The residue was diluted with 50 mL of water, and then the solution was extracted using ethyl acetate (50 mL × 3). Anhydrous sodium sulfate was mixed with the organic layer and allowed to dry overnight. Vacuum filtering was followed by reduced pressure evaporation of the solvent to provide a crude product (light yellow oil 1.59 g, yield 93.01%) that was used straight away for the next stage without additional purification. ESI-MS *m*/*z*: 337.04 $[M + H]^+$.

Synthesis of 4-((6-Bromonaphthalen-2-yl)oxy) Butanoic Acid

In a 100 mL tri-necked bottle containing 4-((6-bromonaphthalen-2-yl)oxy) butanoate (1.59 g, 4.72 mmol), 20 mL of ethanol was added, and the mixture was shaken until it dissolved. Then, 3 mL of water and NaOH (0.75 g, 18.8 mmol) were added to the bottle at 25 °C, and the solution was refluxed for 2 h. Through the use of thin-layer chromatography (TLC), the reaction was observed. After cooling the mixture to 25 °C, it was dried with a rotary evaporator, and the remaining 30 mL was diluted with water. Then, 1N hydrochloric acid was added to the solution to bring its pH to 5, resulting in an amount of solid white precipitate. After vacuum filtration was employed to dry the precipitate, a crude product

(white solid 1.22g g, yield 85.8%) was obtained. This product was used straight away for the following stage without undergoing additional purification. ESI-MS *m/z*: 309.16 [M + H]$^+$.

Synthesis of 4-((6-Bromonaphthalen-2-yl)oxy)-N-(2-oxooxazolidin-3-yl) Butanamide (**Y-2**)

A mixture of 4-((6-bromonaphthalen-2-yl)oxy) butanoic acid (1.22 g, 3.96 mmol) and 20 mL of dichloromethane in a 50 mL tri-necked container was swirled until dissolved. Then, EDCI (0.87 g, 4.55 mmol), HOBt (0.62 g, 4.55 mmol), and triethylamine 1.2 g, 9.25 mmol) were added to a bottle at 0 °C. After agitating in an ice bath for 1 h, 3-amino-2-oxazolidinone (0.4 g, 3.96 mmol) was added again. After heating the mixture to 25 °C, it was stirred overnight. Following TLC, the product underwent rotational evaporation drying after being vacuum-filtered. Column chromatography (CH_2Cl_2: MeOH = 40:1). Compound **Y-2** was obtained as a white solid, 0.78 g, with a yield of 65.33%. ^{1}H NMR (600 MHz, DMSO-d_6) δ 10.21 (s, 1H), 8.11 (d, *J* = 2.1 Hz, 1H), 7.83 (d, *J* = 9.0 Hz, 1H), 7.77 (d, *J* = 8.8 Hz, 1H), 7.56 (dd, *J* = 8.8, 2.1 Hz, 1H), 7.35 (d, *J* = 2.5 Hz, 1H), 7.23 (dd, *J* = 9.0, 2.5 Hz, 1H), 4.37 (t, *J* = 7.8 Hz, 2H), 4.12 (t, *J* = 6.3 Hz, 2H), 3.68 (t, *J* = 7.8 Hz, 2H), 2.37 (t, *J* = 7.4 Hz, 2H), 2.04 (p, *J* = 6.7 Hz, 2H). ^{13}C NMR (151 MHz, DMSO-d_6) δ 171.41, 157.58, 157.30, 133.38, 130.12, 129.83, 129.69, 129.37, 129.07, 120.39, 116.70, 107.27, 67.23, 62.05, 46.34, 29.94, 24.75; ESI-MS *m/z*: 393.0443 [M + H]$^+$.

3.1.4. Synthesis of Target Compound **Y-3**

Synthesis of Ethyl 4-(4-Methoxyphenoxy) Butanoate

A 100 mL three-neck bottle contained ethyl 4-bromobutyrate (1 g, 5.1 mmol), 4-methoxyphenol (0.83 g, 5.1 mmol), and 50 mL of acetonitrile. As cesium carbonate (3.32 g, 10.2 mmol) was added and the mixture was allowed to dissolve, the temperature of the solution rose to 50 °C. After 8 h of stirring, the mixture was allowed to cool to ambient temperature, vacuum-filtered, and then dried using a rotary evaporator. The residue was diluted with 50 mL of water, and then the solution was extracted using ethyl acetate (50 mL × 3). Anhydrous sodium sulfate was mixed with the organic layer and allowed to dry overnight. Vacuum filtering was followed by reduced pressure evaporation of the solvent to provide a crude product (light yellow oil 1.09 g, yield 90.19%) that was used straight away for the next stage without additional purification. ESI-MS *m/z*: 239.12 [M + H]$^+$.

Synthesis of 4-(4-Methoxyphenoxy) Butanoic Acid

In a 100 mL tri-necked bottle containing ethyl 4-(4-methoxyphenoxy) butanoate (1.09 g, 4.6 mmol), 20 mL of ethanol was added, and the mixture was shaken until it dissolved. Then, 3 mL of water and NaOH (0.75 g, 18.8 mmol) were added to the bottle at 25 °C, and the solution was refluxed for 2 h. Through the use of thin-layer chromatography (TLC), the reaction was observed. After cooling the mixture to 25 °C, it was dried with a rotary evaporator, and the remaining 30 mL was diluted with water. Then, 1N hydrochloric acid was added to the solution to bring its pH to 5, resulting in an amount of solid white precipitate. After vacuum filtration was employed to dry the precipitate, a crude product (white solid 0.78 g, yield 80.8%) was obtained. This product was used straight away for the following stage without undergoing additional purification. ESI-MS *m/z*: 211.09 [M + H]$^+$.

Synthesis of 4-(4-Methoxyphenoxy)-N-(2-oxooxazolidin-3-yl) Butanamide (**Y-3**)

A mixture of 4-(4-methoxyphenoxy) butanoic acid (0.78g, 3.71 mmol) and 20 mL of dichloromethane in a 50 mL tri-necked container was swirled until dissolved. Then, EDCI (0.87 g, 4.55mmol), HOBt (0.62g, 4.55mmol), and triethylamine (0.93 g, 9.25mmol) were added to a bottle at 0 °C. After agitating in an ice bath for 1 h, 3-amino-2-oxazolidinone (0.38 g, 3.71mmol) was added again. After heating the mixture to 25 °C, it was stirred overnight. Following TLC, the product underwent rotational evaporation drying after being vacuum-

filtered. Column chromatography (CH_2Cl_2: MeOH = 40:1). Compound **Y-3** was obtained as a white solid, 0.71g, with a yield of 64.98%. ^{1}H NMR (600 MHz, DMSO-d_6) δ 10.17 (s, 1H), 6.88–6.82 (m, 4H), 4.35 (t, J = 7.8 Hz, 2H), 3.91 (t, J = 6.3 Hz, 2H), 3.70–3.63 (m, 5H), 2.29 (t, J = 7.4 Hz, 2H), 1.93 (p, J = 6.8 Hz, 2H). ^{13}C NMR (151 MHz, DMSO-d_6) δ 171.44, 157.56, 153.81, 152.96, 115.86, 115.06, 67.47, 62.03, 55.82, 46.31, 29.94, 24.92; ESI-MS m/z: 295.1289 $[M + H]^+$.

3.1.5. Synthesis of Target Compound **Y-5**

Synthesis of Ethyl 4-([1,1′-Biphenyl]-4-yloxy) Butanoate

A 100 mL three-neck bottle contained ethyl 4-bromobutyrate (1 g, 5.1 mmol), 4-Phenylpheno (0.87 g, 5.1 mmol), and 50 mL of acetonitrile. As cesium carbonate (3.32 g, 10.2 mmol) was added and the mixture was allowed to dissolve, the temperature of the solution rose to 50 °C. After 8 h of stirring, the mixture was allowed to cool to ambient temperature, vacuum-filtered, and then dried using a rotary evaporator. The residue was diluted with 50 mL of water, and then the solution was extracted using ethyl acetate (50 mL × 3). Anhydrous sodium sulfate was mixed with the organic layer and allowed to dry overnight. Vacuum filtering was followed by reduced pressure evaporation of the solvent to provide a crude product (light yellow oil 1.31 g, yield 90.01%) that was used straight away for the next stage without additional purification. ESI-MS m/z: 285. 14 $[M + H]^+$.

Synthesis of 4-([1,1′-Biphenyl]-4-yloxy) Butanoic Acid

In a 100 mL tri-necked bottle containing ethyl 4-([1,1′-biphenyl]-4-yloxy) butanoate (1.31 g, 4.59 mmol), 30 mL of ethanol was added, and the mixture was shaken until it dissolved. Then, 3 mL of water and NaOH (0.85 g, 21.2 mmol) were added to the bottle at 25 °C, and the solution was refluxed for 2 h. Through the use of thin-layer chromatography (TLC), the reaction was observed. After cooling the mixture to 25 °C, it was dried with a rotary evaporator, and the remaining 30 mL was diluted with water. Then, 1N hydrochloric acid was added to the solution to bring its pH to 5, resulting in an amount of solid white precipitate. After vacuum filtration was employed to dry the precipitate, a crude product (white solid 0.94 g, yield 80.7%) was obtained. This product was used straight away for the following stage without undergoing additional purification. ESI-MS m/z: 257.11 $[M + H]^+$.

Synthesis of 4-([1,1′-Biphenyl]-4-yloxy)-N-(2-oxooxazolidin-3-yl) Butanamide (**Y-5**)

A mixture of 4-([1,1′-biphenyl]-4-yloxy) butanoic acid (0.94 g, 3.67 mmol) and 20 mL of dichloromethane in a 50 mL tri-necked container was swirled until dissolved. Then, EDCI (0.87 g, 4.55 mmol), HOBt (0.62 g, 4.55 mmol), and triethylamine (0.93 g, 9.25 mmol) were added to a bottle at 0 °C. After agitating in an ice bath for 1 h, 3-amino-2-oxazolidinone (0.37 g, 3.67 mmol) was added again. After heating the mixture to 25 °C, it was stirred overnight. Following TLC, the product underwent rotational evaporation drying after being vacuum-filtered. Column chromatography (CH_2Cl_2: MeOH = 40:1). Compound **Y-5** was obtained as a white solid, 0.84g, with a yield of 66.2%.^{1}H NMR (600 MHz, DMSO-d_6) δ 10.20 (s, 1H), 7.63–7.56 (m, 4H), 7.43 (t, J = 7.8 Hz, 2H), 7.33–7.27 (m, 1H), 7.05–6.99 (m, 2H), 4.36 (t, J = 7.7 Hz, 2H), 4.04 (t, J = 6.4 Hz, 2H), 3.67 (t, J = 7.8 Hz, 2H), 2.33 (t, J = 7.4 Hz, 2H), 1.99 (p, J = 6.8 Hz, 2H). ^{13}C NMR (151 MHz, DMSO-d_6) δ 158.60, 157.57, 140.31, 133.02, 129.33, 128.23, 127.17, 126.63, 115.40, 67.10, 62.04, 46.33, 29.91, 24.84; ESI-MS m/z: 341.1496 $[M + H]^+$.

3.1.6. Synthesis of Target Compound **Y-8**

Synthesis of Ethyl 2-(4-Nitrophenoxy) Acetate

A 100 mL three-neck bottle contained ethyl Ethyl bromoacetate (1.03 g, 5.98 mmol), 4-nitrophenol (0.83 g, 5.98 mmol), and 50 mL of acetonitrile. As cesium carbonate (3.11 g, 9.52

mmol) was added and the mixture was allowed to dissolve, the temperature of the solution rose to 50 °C. After 8 h of stirring, the mixture was allowed to cool to ambient temperature, vacuum-filtered, and then dried using a rotary evaporator. The residue was diluted with 50 mL of water, and then the solution was extracted using ethyl acetate (50 mL × 3). Anhydrous sodium sulfate was mixed with the organic layer and allowed to dry overnight. Vacuum filtering was followed by reduced pressure evaporation of the solvent to provide a crude product (light yellow oil 1.28 g, yield 95.6%) that was used straight away for the next stage without additional purification. ESI-MS *m/z*: 226.07 $[M + H]^+$.

Synthesis of 2-(4-Nitrophenoxy) Acetic Acid

In a 100 mL tri-necked bottle containing ethyl 2-(4-nitrophenoxy) acetate (1.28 g, 5.71 mmol), 20 mL of ethanol was added, and the mixture was shaken until it dissolved. Then, 3 mL of water and NaOH (0.76 g, 18.8 mmol) were added to the bottle at 25 °C, and the solution was refluxed for 2 h. Through the use of thin-layer chromatography (TLC), the reaction was observed. After cooling the mixture to 25 °C, it was dried with a rotary evaporator, and the remaining 30 mL was diluted with water. Then, 1N hydrochloric acid was added to the solution to bring its pH to 5, resulting in an amount of solid white precipitate. After vacuum filtration was employed to dry the precipitate, a crude product (white solid 0.91 g, yield 81.7%) was obtained. This product was used straight away for the following stage without undergoing additional purification. ESI-MS *m/z*: 198.04 $[M + H]^+$.

Synthesis of 2-(4-Nitrophenoxy)-N-(2-oxooxazolidin-3-yl) Acetamide (**Y-8**)

A mixture of 2-(4-nitrophenoxy) acetic acid (0.91g, 4.62 mmol) and 20 mL of dichloromethane in a 50 mL tri-necked container was swirled until dissolved. Then, EDCI (1.05 g, 5.54 mmol), HOBt (0.75 g, 5.54 mmol), and triethylamine (0.84 g, 9.25 mmol) were added to a bottle at 0 °C. After agitating in an ice bath for 1 h, 3-amino-2-oxazolidinone (0.47 g, 4.62 mmol) was added again. After heating the mixture to 25 °C, it was stirred overnight. Following TLC, the product underwent rotational evaporation drying after being vacuum-filtered. Column chromatography (CH_2Cl_2: MeOH = 40:1). The compound **Y-8** was obtained as a white solid, 0.78g, with a yield of 60.1%.^{1}H NMR (600 MHz, DMSO-d_6) δ 10.60 (s, 1H), 8.27–8.18 (m, 2H), 7.23–7.17 (m, 2H), 4.85 (s, 2H), 4.41 (t, *J* = 7.7 Hz, 2H), 3.70 (t, *J* = 7.7 Hz, 2H). ^{13}C NMR (151 MHz, DMSO-d_6) δ 166.75, 163.12, 157.37, 141.92, 126.26, 126.16, 115.87, 115.63, 66.74, 62.22, 47.55, 46.30; ESI-MS *m/z*: 282.0725 $[M + H]^+$.

3.1.7. Synthesis of Target Compound **Y-9**

Synthesis of Ethyl 4-((6-Bromopyridin-3-yl)oxy) Butanoate

A 100 mL three-neck bottle contained ethyl 4-bromobutyrate (1 g, 5.1 mmol), 2-Bromo-5-hydroxypyridine (0.89 g, 5.1 mmol), and 50 mL of acetonitrile. As cesium carbonate (3.32 g, 10.2 mmol) was added and the mixture was allowed to dissolve, the temperature of the solution rose to 50 °C. After 8 h of stirring, the mixture was allowed to cool to ambient temperature, vacuum-filtered, and then dried using a rotary evaporator. The residue was diluted with 50 mL of water, and then the solution was extracted using ethyl acetate (50 mL × 3). Anhydrous sodium sulfate was mixed with the organic layer and allowed to dry overnight. Vacuum filtering was followed by reduced pressure evaporation of the solvent to provide a crude product (light yellow oil 1.35 g, yield 92.01%) that was used straight away for the next stage without additional purification. ESI-MS *m/z*: 289.01 $[M + H]^+$.

Synthesis of 4-((6-Bromopyridin-3-yl)oxy) Butanoic Acid

In a 100 mL tri-necked bottle containing ethyl 4-((6-bromopyridin-3-yl)oxy) butanoate (1.35 g, 4.69 mmol), 20 mL of ethanol was added, and the mixture was shaken until it dissolved.

Then, 3 mL of water and NaOH (0.85 g, 21.2 mmol) were added to the bottle at 25 °C, and the solution was refluxed for 2 h. Through the use of thin-layer chromatography (TLC), the reaction was observed. After cooling the mixture to 25 °C, it was dried with a rotary evaporator, and the remaining 30 mL was diluted with water. Then, 1N hydrochloric acid was added to the solution to bring its pH to 5, resulting in an amount of solid white precipitate. After vacuum filtration was employed to dry the precipitate, a crude product (white solid 0.98 g, yield 80.8%) was obtained. This product was used straight away for the following stage without undergoing additional purification. ESI-MS *m*/*z*: 260.98 $[M + H]^+$.

Synthesis of 4-((6-Bromopyridin-3-yl)oxy)-N-(2-oxooxazolidin-3-yl) Butanamide (**Y-9**)

A mixture of 4-((6-bromopyridin-3-yl)oxy) butanoic acid (0.98 g, 3.79 mmol) and 20 mL of dichloromethane in a 50 mL tri-necked container was swirled until dissolved. Then, EDCI (0.87 g, 4.55 mmol), HOBt 0.62 g, 4.55 mmol), and triethylamine (0.93 g, 9.25 mmol) were added to a bottle at 0 °C. After agitating in an ice bath for 1 h, 3-amino-2-oxazolidinone (0.39 g, 3.79 mmol) was added again. After heating the mixture to 25 °C, it was stirred overnight. Following TLC, the product underwent rotational evaporation drying after being vacuum-filtered. Column chromatography (CH_2Cl_2: MeOH = 40:1). Compound **Y-9** was obtained as a white solid, 0.84g, with a yield of 65.33%. ^{1}H NMR (600 MHz, DMSO-d_6) δ 10.19 (s, 1H), 8.12 (d, *J* = 3.2 Hz, 1H), 7.54 (d, *J* = 8.7 Hz, 1H), 7.39 (dd, *J* = 8.7, 3.2 Hz, 1H), 4.36 (t, *J* = 7.8 Hz, 2H), 4.07 (t, *J* = 6.3 Hz, 2H), 3.66 (t, *J* = 7.8 Hz, 2H), 2.31 (t, *J* = 7.3 Hz, 2H), 1.98 (p, *J* = 6.7 Hz, 2H). ^{13}C NMR (151 MHz, DMSO-d_6) δ 171.30, 157.56, 155.23, 138.46, 138.40, 131.65, 128.71, 125.92, 68.02, 62.05, 46.33, 29.72, 24.57; ESI-MS *m*/*z*: 344.0244 $[M + H]^+$.

3.1.8. Synthesis of Target Compound **Y-11**

Synthesis of Ethyl 4-(4-(Methylsulfonyl)phenoxy) Butanoate

A 100 mL three-neck bottle contained ethyl 4-bromobutyrate (1 g, 5.1 mmol), 4-(Methylsulfonyl) phenol (0.88 g, 5.1 mmol), and 50 mL of acetonitrile. As cesium carbonate (3.32 g, 10.2 mmol) was added and the mixture was allowed to dissolve, the temperature of the solution rose to 50 °C. After 8 h of stirring, the mixture was allowed to cool to ambient temperature, vacuum-filtered, and then dried using a rotary evaporator. The residue was diluted with 50 mL of water, and then the solution was extracted using ethyl acetate (50 mL × 3). Anhydrous sodium sulfate was mixed with the organic layer and allowed to dry overnight. Vacuum filtering was followed by reduced pressure evaporation of the solvent to provide a crude product (light yellow oil 1.32 g, yield 91.7%) that was used straight away for the next stage without additional purification. ESI-MS *m*/*z*: 287.09 $[M + H]^+$.

Synthesis of 4-(4-(Methylsulfonyl)phenoxy) Butanoic Acid

In a 100 mL tri-necked bottle containing ethyl 4-(4-(methylsulfonyl)phenoxy) butanoate (1.32 g, 4.64 mmol), 20 mL of ethanol was added, and the mixture was shaken until it dissolved. Then, 3 mL of water and NaOH (0.75 g, 18.8 mmol) were added to the bottle at 25 °C, and the solution was refluxed for 2 h. Through the use of thin-layer chromatography (TLC), the reaction was observed. After cooling the mixture to 25 °C, it was dried with a rotary evaporator, and the remaining 30 mL was diluted with water. Then, 1N hydrochloric acid was added to the solution to bring its pH to 5, resulting in an amount of solid white precipitate. After vacuum filtration was employed to dry the precipitate, a crude product (white solid 1.06 g, yield 89.3%) was obtained. This product was used straight away for the following stage without undergoing additional purification. ESI-MS *m*/*z*: 259.06 $[M + H]^+$.

Synthesis of 4-(4-(Methylsulfonyl)phenoxy)-N-(2-oxooxazolidin-3-yl) Butanamide (**Y-11**)

A mixture of 4-(4-(methylsulfonyl)phenoxy) butanoic acid (1.06 g, 4.12 mmol) and 20 mL of dichloromethane in a 50 mL tri-necked container was swirled until dissolved. Then, EDCI (0.85 g, 4.44 mmol), HOBt (0.6 g, 4.44 mmol), and triethylamine (0.84 g, 9.25 mmol) were added to a bottle at 0 °C. After agitating in an ice bath for 1 h, 3-amino-2-oxazolidinone (0.42 g, 4.12 mmol) was added again. After heating the mixture to 25°C, it was stirred overnight. Following TLC, the product underwent rotational evaporation drying after being vacuum-filtered. Column chromatography (CH_2Cl_2: MeOH = 40:1). Compound **Y-11** was obtained as a white solid, 0.84g, with a yield of 60.5%. ^{1}H NMR (600 MHz, DMSO-d_6) δ 10.19 (s, 1H), 7.86–7.81 (m, 2H), 7.18–7.13 (m, 2H), 4.36 (t, *J* = 7.8 Hz, 2H), 4.11 (t, *J* = 6.3 Hz, 2H), 3.66 (t, *J* = 7.8 Hz, 2H), 3.15 (s, 3H), 2.33 (t, *J* = 7.3 Hz, 2H), 2.00 (p, *J* = 6.7 Hz, 2H).^{13}C NMR (151 MHz, DMSO-d_6) δ 171.32, 162.76, 157.57, 133.03, 129.68, 115.43, 67.70, 62.05, 46.33, 44.44, 40.53, 29.77, 24.59; ESI-MS *m*/*z*: 343.0957 [M + H]$^+$.

3.1.9. Synthesis of Target Compound **Y-15**

Synthesis of Ethyl 4-((5-Bromopyrazin-2-yl)oxy) Butanoate

A 100 mL three-neck bottle contained ethyl 4-bromobutyrate (1 g, 5.1 mmol), 2-BroMo-5-hydroxypyriMidine (0.89 g, 5.1 mmol), and 50 mL of acetonitrile. As cesium carbonate (3.32 g, 10.2 mmol) was added and the mixture was allowed to dissolve, the temperature of the solution rose to 50 °C. After 8 h of stirring, the mixture was allowed to cool to ambient temperature, vacuum-filtered, and then dried using a rotary evaporator. The residue was diluted with 50 mL of water, and then the solution was extracted using ethyl acetate (50 mL × 3). Anhydrous sodium sulfate was mixed with the organic layer and allowed to dry overnight. Vacuum filtering was followed by reduced pressure evaporation of the solvent to provide a crude product (light yellow oil 1.37 g, yield 93.01%) that was used straight away for the next stage without additional purification. ESI-MS *m*/*z*: 290.01 [M + H]$^+$.

Synthesis of 4-((5-Bromopyrazin-2-yl)oxy) Butanoic Acid

In a 100 mL tri-necked bottle containing ethyl 4-((5-bromopyrazin-2-yl)oxy) butanoate (1.37 g, 4.74 mmol), 20 mL of ethanol was added, and the mixture was shaken until it dissolved. Then, 3 mL of water and NaOH (0.85 g, 21.2 mmol) were added to the bottle at 25 °C, and the solution was refluxed for 2 h. Through the use of thin-layer chromatography (TLC), the reaction was observed. After cooling the mixture to 25 °C, it was dried with a rotary evaporator, and the remaining 30 mL was diluted with water. Then, 1N hydrochloric acid was added to the solution to bring its pH to 5, resulting in an amount of solid white precipitate. After vacuum filtration was employed to dry the precipitate, a crude product (white solid 1.02 g, yield 82.8%) was obtained. This product was used straight away for the following stage without undergoing additional purification. ESI-MS *m*/*z*: 261.98 [M + H]$^+$.

Synthesis of 4-((5-Bromopyrazin-2-yl)oxy)-N-(2-oxooxazolidin-3-yl) Butanamide (**Y-15**)

A mixture of 4-((5-bromopyrazin-2-yl)oxy) butanoic acid (1.02 g, 3.92 mmol) and 20 mL of dichloromethane in a 50 mL tri-necked container was swirled until dissolved. Then, EDCI (0.87 g, 4.55 mmol), HOBt (0.62 g, 4.55 mmol), and triethylamine (0.84 g, 9.25 mmol) were added to a bottle at 0 °C. After agitating in an ice bath for 1 h, 3-amino-2-oxazolidinone (0.4 g, 3.92 mmol) was added again. After heating the mixture to 25°C, it was stirred overnight. Following TLC, the product underwent rotational evaporation drying after being vacuum-filtered. Column chromatography (CH_2Cl_2: MeOH = 40:1). Compound **Y-15** was obtained as a white solid, 0.89g, with a yield of 66.45%.^{1}H NMR (600 MHz, DMSO-d_6') δ 10.17 (s, 1H), 8.41 (d, *J* = 1.4 Hz, 1H), 8.18 (d, *J* = 1.4 Hz, 1H), 4.35 (t, *J* = 7.8 Hz, 2H), 4.31 (t, *J* = 6.4 Hz, 2H), 3.65 (t, *J* = 7.8 Hz, 2H), 2.30 (t, *J* = 7.3 Hz, 2H), 1.99 (h, *J* = 6.5 Hz, 2H). ^{13}C NMR (151 MHz, DMSO-d_6) δ

171.24, 159.77, 157.53, 143.25, 136.09, 130.11, 66.50, 62.03, 46.30, 29.85, 24.33; ESI-MS *m*/*z*: 345.0192 $[M + H]^+$.

3.1.10. Synthesis of Target Compound **Y-16**

Synthesis of Ethyl 2-(4-Chlorophenoxy) Acetate

A 100 mL three-neck bottle contained Ethyl bromoacetate (1.03 g, 6.00 mmol), 4-chlorophenol (0.77 g, 6 mmol), and 50 mL of acetonitrile. As cesium carbonate (3.92 g, 12.00 mmol) was added and the mixture was allowed to dissolve, the temperature of the solution rose to 50 °C. After 8 h of stirring, the mixture was allowed to cool to ambient temperature, vacuum-filtered, and then dried using a rotary evaporator. The residue was diluted with 50 mL of water, and then the solution was extracted using ethyl acetate (50 mL × 3). Anhydrous sodium sulfate was mixed with the organic layer and allowed to dry overnight. Vacuum filtering was followed by reduced pressure evaporation of the solvent to provide a crude product (light yellow oil 1.22 g, yield 95.6%) that was used straight away for the next stage without additional purification. ESI-MS *m*/*z*: 215.04 $[M + H]^+$.

Synthesis of 2-(4-Chlorophenoxy) Acetic Acid

In a 100 mL tri-necked bottle containing ethyl 2-(4-chlorophenoxy) acetate (0.79 g, 4.30 mmol), 20 mL of ethanol was added, and the mixture was shaken until it dissolved. Then, 3 mL of water and NaOH (0.75 g, 18.8 mmol) were added to the bottle at 25 °C, and the solution was refluxed for 2 h. Through the use of thin-layer chromatography (TLC), the reaction was observed. After cooling the mixture to 25 °C, it was dried with a rotary evaporator, and the remaining 30 mL was diluted with water. Then, 1N hydrochloric acid was added to the solution to bring its pH to 5, resulting in an amount of solid white precipitate. After vacuum filtration was employed to dry the precipitate, a crude product (white solid 0.79 g, yield 71.7%) was obtained. This product was used straight away for the following stage without undergoing additional purification. ESI-MS *m*/*z*: 186.01 $[M + H]^+$.

Synthesis of 2-(4-Chlorophenoxy)-N-(2-Oxooxazolidin-3-yl) Acetamide (**Y-16**)

A mixture of 2-(4-chlorophenoxy) acetic acid (0.79 g, 4.30 mmol) and 20 mL of dichloromethane in a 50 mL tri-necked container was swirled until dissolved. Then, EDCI (0.85 g, 4.44 mmol), HOBt (0.6 g, 4.44 mmol), and triethylamine (0.84 g, 9.25 mmol) were added to a bottle at 0 °C. After agitating in an ice bath for 1 h, 3-amino-2-oxazolidinone (0.44 g, 4.30 mmol) was added again. After heating the mixture to 25°C, it was stirred overnight. Following TLC, the product underwent rotational evaporation drying after being vacuum-filtered. Column chromatography (CH_2Cl_2: MeOH = 40:1). Compound **Y-16** was obtained as a white solid, 0.71g, with a yield of 52.6%. ^{1}H-NMR(400 MHz, DMSO- *d*$_6$) δ 10.10 (s, 1H), 7.07 (d, *J*=8.0 Hz, 1H), 6.69-6.75 (m, 3H), 4.31 (t, *J*=8.0 Hz, 2H), 3.90 (t, *J*=8.0 Hz, 2H), 3.62 (t, *J*=8.0 Hz, 2H), 2.47 (t, *J*=8.0 Hz, 2H), 2.11-1.65 (m, 4H); ^{13}C-NMR (DMSO-*d*$_6$):δ 171.74, 164.76, 162.34, 160.71, 157.66, 131.23, 111.38, 107.68, 102.52, 67.98, 62.08, 46.38, 33.01, 28.36, 21.94. ESI-MS *m*/*z*: 293.0602 $[M + H]^+$.

3.1.11. Synthesis of Target Compound **Y-17**

Synthesis of Ethyl 4-(4-butylphenoxy) Butanoate

A 100 mL three-neck bottle contained ethyl 4-bromobutyrate (1 g, 5.1 mmol), 4-Butylphenol (0.76 g, 5.1 mmol), and 50 mL of acetonitrile. As cesium carbonate (3.32 g, 10.2 mmol) was added and the mixture was allowed to dissolve, the temperature of the solution rose to 50 °C. After 8 h of stirring, the mixture was allowed to cool to ambient temperature, vacuum-filtered, and then dried using a rotary evaporator. The residue was diluted with 50 mL of

water, and then the solution was extracted using ethyl acetate (50 mL × 3). Anhydrous sodium sulfate was mixed with the organic layer and allowed to dry overnight. Vacuum filtering was followed by reduced pressure evaporation of the solvent to provide a crude product (light yellow oil 1.31 g, yield 97.6%) that was used straight away for the next stage without additional purification. ESI-MS *m*/*z*: 264.17 $[M + H]^+$.

Synthesis of 4-(4-Butylphenoxy) Butanoic Acid

In a 100 mL tri-necked bottle containing ethyl 4-(4-butylphenoxy) butanoate (1.31 g, 4.98 mmol), 20 mL of ethanol was added, and the mixture was shaken until it dissolved. Then, 3 mL of water and NaOH (0.75 g, 18.8 mmol) were added to the bottle at 25 °C, and the solution was refluxed for 2 h. Through the use of thin-layer chromatography (TLC), the reaction was observed. After cooling the mixture to 25 °C, it was dried with a rotary evaporator, and the remaining 30 mL was diluted with water. Then, 1N hydrochloric acid was added to the solution to bring its pH to 5, resulting in an amount of solid white precipitate. After vacuum filtration was employed to dry the precipitate, a crude product (white solid 0.84 g, yield 71.7%) was obtained. This product was used straight away for the following stage without undergoing additional purification. ESI-MS *m*/*z*: 237.14 $[M + H]^+$.

Synthesis of 4-(4-Butylphenoxy)-N-(2-oxooxazolidin-3-yl) Butanamide (**Y-17**)

A mixture of 4-(4-butylphenoxy) butanoic acid (0.84g, 3.57 mmol) and 20 mL of dichloromethane in a 50 mL tri-necked container was swirled until dissolved. Then, EDCI (0.85 g, 4.44 mmol), HOBt (0.6 g, 4.44 mmol), and triethylamine (0.84 g, 9.25 mmol) were added to a bottle at 0 °C. After agitating in an ice bath for 1 h, 3-amino-2-oxazolidinone (0.36 g, 3.57 mmol) was added again. After heating the mixture to 25 °C, it was stirred overnight. Following TLC, the product underwent rotational evaporation drying after being vacuum-filtered. Column chromatography (CH_2Cl_2: MeOH = 40:1). Compound **Y-17** is obtained as a white solid, 0.7g, with a yield of 62.9%. ^{1}H-NMR(400 MHz, DMSO-d_6) δ 10.18 (s, 1H), 7.24 (t, *J*=8.0 Hz, 1H), 7.19-7.12 (m, 1H), 6.93 (t, *J*=8.0 Hz, 2H), 4.32 (t, *J*=8.0 Hz, 2H), 4.00 (t, *J*=8.0 Hz, 2H), 3.63 (t, *J*=8.0 Hz, 2H), 2.46 (t, *J*=8.0 Hz, 2H), 1.94-1.92 (m, 2H); ^{13}C-NMR (DMSO-d_6):δ 171.39, 157.67, 154.87, 153.11, 143.67, 116.27, 111.44, 105.29, 68.84, 62.11, 46.37, 29.75, 24.79. ESI-MS *m*/*z*: 343.1629$[M + H]^+$.

3.1.12. Synthesis of Target Compound **Y-18**

Synthesis of Ethyl 4-(4-(Tert-butoxy)phenoxy) Butanoate

A 100 mL three-neck bottle contained ethyl 4-bromobutyrate (1 g, 5.1 mmol), 4-T-butoxyphenol (0.84 g, 5.1 mmol), and 50 mL of acetonitrile. As cesium carbonate (3.32 g, 10.2 mmol) was added and the mixture was allowed to dissolve, the temperature of the solution rose to 50 °C. After 8 h of stirring, the mixture was allowed to cool to ambient temperature, vacuum-filtered, and then dried using a rotary evaporator. The residue was diluted with 50 mL of water, and then the solution was extracted using ethyl acetate (50 mL × 3). Anhydrous sodium sulfate was mixed with the organic layer and allowed to dry overnight. Vacuum filtering was followed by reduced pressure evaporation of the solvent to provide a crude product (light yellow oil 1.35 g, yield 94.6%) that was used straight away for the next stage without additional purification. ESI-MS *m*/*z*: 281.17 $[M + H]^+$.

Synthesis of 4-(4-(Tert-butoxy)phenoxy) Butanoic Acid

In a 100 mL tri-necked bottle containing ethyl 4-(4-(tert-butoxy)phenoxy) butanoate (1.35 g, 4.82 mmol), 20 mL of ethanol was added, and the mixture was shaken until it dissolved. Then, 3 mL of water and NaOH (0.75 g, 18.8 mmol) were added to the bottle at 25 °C, and the solution was refluxed for 2 h. Through the use of thin-layer chromatography (TLC), the

reaction was observed. After cooling the mixture to 25 °C, it was dried with a rotary evaporator, and the remaining 30 mL was diluted with water. Then, 1N hydrochloric acid was added to the solution to bring its pH to 5, resulting in an amount of solid white precipitate. After vacuum filtration was employed to dry the precipitate, a crude product (white solid 0.96 g, yield 78.7%) was obtained. This product was used straight away for the following stage without undergoing additional purification. ESI-MS *m/z*: 253.14 $[M + H]^+$.

Synthesis of 4-(4-(Tert-butoxy)phenoxy)-N-(2-oxooxazolidin-3-yl) Butanamide (**Y-18**)

A mixture of 4-(4-(tert-butoxy)phenoxy) butanoic acid (0.96 g, 3.79 mmol) and 20 mL of dichloromethane in a 50 mL tri-necked container was swirled until dissolved. Then, EDCI (0.85 g, 4.44 mmol), HOBt (0.6 g, 4.44 mmol), and triethylamine (0.84 g, 9.25 mmol) were added to a bottle at 0 °C. After agitating in an ice bath for 1 h, 3-amino-2-oxazolidinone (0.39 g, 3.79 mmol) was added again. After heating the mixture to 25 °C, it was stirred overnight. Following TLC, the product underwent rotational evaporation drying after being vacuum-filtered. Column chromatography (CH_2Cl_2: MeOH = 40:1). Compound **Y-18** was obtained as a white solid, 0.79g, with a yield of 62.2%. ^1H NMR (600 MHz, DMSO-d_6) δ 10.18 (s, 1H), 6.90–6.85 (m, 2H), 6.85–6.79 (m, 2H), 4.36 (t, *J* = 7.8 Hz, 2H), 3.93 (t, *J* = 6.3 Hz, 2H), 3.66 (t, *J* = 7.8 Hz, 2H), 2.30 (t, *J* = 7.4 Hz, 2H), 1.94 (p, *J* = 6.7 Hz, 2H), 1.23 (s, 9H). ^{13}C NMR (151 MHz, DMSO-d_6) δ 171.44, 157.57, 148.63, 125.48, 114.99, 77.82, 67.21, 62.03, 46.32, 29.95, 28.92, 24.93; ESI-MS *m/z*: 336.1685 $[M + H]^+$.

3.1.13. Synthesis of Target Compound **Y-19**

Synthesis of Ethyl 4-(4-ethylphenoxy) Butanoate

A 100 mL three-neck bottle contained ethyl 4-bromobutyrate (1 g, 5.1 mmol), 4-ethyl-pheno (0.61 g, 5.1 mmol), and 50 mL of acetonitrile. As cesium carbonate (3.32 g, 10.2 mmol) was added and the mixture was allowed to dissolve, the temperature of the solution rose to 50 °C. After 8 h of stirring, the mixture was allowed to cool to ambient temperature, vacuum-filtered, and then dried using a rotary evaporator. The residue was diluted with 50 mL of water, and then the solution was extracted using ethyl acetate (50 mL × 3). Anhydrous sodium sulfate was mixed with the organic layer and allowed to dry overnight. Vacuum filtering was followed by reduced pressure evaporation of the solvent to provide a crude product (light yellow oil 1.15 g, yield 95.9%) that was used straight away for the next stage without additional purification. ESI-MS *m/z*: 237.14 $[M + H]^+$.

Synthesis of 4-(4-Ethylphenoxy) Butanoic Acid

In a 100 mL tri-necked bottle containing ethyl 4-(4-ethylphenoxy) butanoate (1.2 g, 4.63 mmol), 20 mL of ethanol was added, and the mixture was shaken until it dissolved. Then, 3 mL of water and NaOH (0.75 g, 18.8 mmol) were added to the bottle at 25 °C, and the solution was refluxed for 2 h. Through the use of thin-layer chromatography (TLC), the reaction was observed. After cooling the mixture to 25 °C, it was dried with a rotary evaporator, and the remaining 30 mL was diluted with water. Then, 1N hydrochloric acid was added to the solution to bring its pH to 5, resulting in an amount of solid white precipitate. After vacuum filtration was employed to dry the precipitate, a crude product (white solid 0.79 g, yield 78.6%) was obtained. This product was used straight away for the following stage without undergoing additional purification. ESI-MS *m/z*: 209.11 $[M + H]^+$.

Synthesis of 4-(4-Ethylphenoxy)-N-(2-oxooxazolidin-3-yl) Butanamide (**Y-19**)

A mixture of 4-(4-ethylphenoxy) butanoic acid (0.79 g, 3.84 mmol) and 20 mL of dichloromethane in a 50 mL tri-necked container was swirled until dissolved. Then, EDCI (0.85 g, 4.44 mmol), HOBt (0.6 g, 4.44 mmol), and triethylamine (0.84 g, 9.25 mmol) were

added to a bottle at 0 °C. After agitating in an ice bath for 1 h, 3-amino-2-oxazolidinone (0.39 g, 3.84 mmol) was added again. After heating the mixture to 25 °C, it was stirred overnight. Following TLC, the product underwent rotational evaporation drying after being vacuum-filtered. Column chromatography (CH_2Cl_2: MeOH = 40:1). Compound **Y-19** was obtained as a white solid, 0.66g, with a yield of 58.9%. ^{1}H NMR (600 MHz, DMSO-d_6) δ 10.17 (s, 1H), 7.12–7.07 (m, 2H), 6.86–6.80 (m, 2H), 4.35 (t, *J* = 7.8 Hz, 2H), 3.94 (t, *J* = 6.4 Hz, 2H), 3.66 (t, *J* = 7.8 Hz, 2H), 2.56–2.50 (m, 2H), 2.30 (t, *J* = 7.4 Hz, 2H), 1.94 (p, *J* = 6.8 Hz, 2H), 1.14 (t, *J* = 7.6 Hz, 3H). ^{13}C NMR (151 MHz, DMSO-d_6) δ 171.44, 157.56, 157.01, 136.17, 129.09, 114.79, 114.76, 66.95, 62.03, 46.32, 29.94, 27.76, 24.88, 16.41; ESI-MS *m*/*z*: 293.1506 $[M + H]^+$.

3.1.14. Synthesis of Target Compound **Y-25**

Synthesis of Ethyl 4-(4-(Trifluoromethyl)phenoxy) Butanoate

A 100 mL three-neck bottle contained ethyl 4-bromobutyrate (1 g, 5.1 mmol), 4-Trifluoromethylphenol (0.88 g, 5.1 mmol), and 50 mL of acetonitrile. As cesium carbonate (3.32 g, 10.2 mmol) was added and the mixture was allowed to dissolve, the temperature of the solution rose to 50 °C. After 8 h of stirring, the mixture was allowed to cool to ambient temperature, vacuum-filtered, and then dried using a rotary evaporator. The residue was diluted with 50 mL of water, and then the solution was extracted using ethyl acetate (50 mL × 3). Anhydrous sodium sulfate was mixed with the organic layer and allowed to dry overnight. Vacuum filtering was followed by reduced pressure evaporation of the solvent to provide a crude product (light yellow oil 1.27 g, yield 90.5%) that was used straight away for the next stage without additional purification. ESI-MS *m*/*z*: 277.10 $[M + H]^+$.

Synthesis of 4-(4-(Trifluoromethyl)phenoxy) Butanoic Acid

In a 100 mL tri-necked bottle containing ethyl 4-(4-(trifluoromethyl)phenoxy) butanoate (1.27 g, 4.62 mmol), 20 mL of ethanol was added, and the mixture was shaken until it dissolved. Then, 3 mL of water and NaOH (0.75 g, 18.8 mmol) were added to the bottle at 25 °C, and the solution was refluxed for 2 h. Through the use of thin-layer chromatography (TLC), the reaction was observed. After cooling the mixture to 25 °C, it was dried with a rotary evaporator, and the remaining 30 mL was diluted with water. Then, 1N hydrochloric acid was added to the solution to bring its pH to 5, resulting in an amount of solid white precipitate. After vacuum filtration was employed to dry the precipitate, a crude product (white solid 0.97 g, yield 84.6%) was obtained. This product was used straight away for the following stage without undergoing additional purification. ESI-MS *m*/*z*: 248.07 $[M + H]^+$.

Synthesis of N-(2-Oxooxazolidin-3-yl)-4-(4-(trifluoromethyl)phenoxy) Butanamide (**Y-25**)

A mixture of 4-(4-(trifluoromethyl)phenoxy) butanoic acid (0.97 g, 3.91 mmol) and 20 mL of dichloromethane in a 50 mL tri-necked container was swirled until dissolved. Then, EDCI (0.85 g, 4.44 mmol), HOBt (0.6 g, 4.44 mmol), and triethylamine (0.84 g, 9.25 mmol) were added to a bottle at 0 °C. After agitating in an ice bath for 1 h, 3-amino-2-oxazolidinone (0.40 g, 3.91 mmol) was added again. After heating the mixture to 25 °C, it was stirred overnight. Following TLC, the product underwent rotational evaporation drying after being vacuum-filtered. Column chromatography (CH_2Cl_2: MeOH = 40:1). Compound **Y-25** was obtained as a white solid, 0.67g, with a yield of 57.8%. H NMR (600 MHz, DMSO-d_6) δ 10.19 (s, 1H), 7.67–7.62 (m, 2H), 7.12 (d, *J* = 8.6 Hz, 2H), 4.36 (t, *J* = 7.8 Hz, 2H), 4.08 (t, *J* = 6.4 Hz, 2H), 3.66 (t, *J* = 7.8 Hz, 2H), 2.33 (t, *J* = 7.3 Hz, 2H), 1.99 (p, *J* = 6.8 Hz, 2H). ^{13}C NMR (151 MHz, DMSO-d_6) δ 171.34, 161.83, 157.57, 127.44, 127.42, 127.39, 127.37, 125.95, 124.16, 121.64, 121.42, 115.41, 67.47, 62.04, 46.33, 29.80, 24.62; ESI-MS *m*/*z*: 333.1060 $[M + H]^+$.

3.1.15. Synthesis of Target Compound **Y-26**

Synthesis of Ethyl 4-(4-iodophenoxy) Butanoate

A 100 mL three-neck bottle contained ethyl 4-bromobutyrate (1 g, 5.1 mmol), 4-Iodophenol (1.12 g, 5.1 mmol), and 50 mL of acetonitrile. As cesium carbonate (3.32 g, 10.2 mmol) was added and the mixture was allowed to dissolve, the temperature of the solution rose to 50 °C. After 8 h of stirring, the mixture was allowed to cool to ambient temperature, vacuum-filtered, and then dried using a rotary evaporator. The residue was diluted with 50 mL of water, and then the solution was extracted using ethyl acetate (50 mL × 3). Anhydrous sodium sulfate was mixed with the organic layer and allowed to dry overnight. Vacuum filtering was followed by reduced pressure evaporation of the solvent to provide a crude product (light yellow oil 1.63 g, yield 95.5%) that was used straight away for the next stage without additional purification. ESI-MS *m*/*z*: 335.01 $[M + H]^+$.

Synthesis of 4-(4-Iodophenoxy) Butanoic Acid

In a 100 mL tri-necked bottle containing ethyl 4-(4-iodophenoxy) butanoate (1.63 g, 4.87 mmol), 20 mL of ethanol was added, and the mixture was shaken until it dissolved. Then, 3 mL of water and NaOH (0.75 g, 18.8 mmol) were added to the bottle at 25 °C, and the solution was refluxed for 2 h. Through the use of thin-layer chromatography (TLC), the reaction was observed. After cooling the mixture to 25 °C, it was dried with a rotary evaporator, and the remaining 30 mL was diluted with water. Then, 1N hydrochloric acid was added to the solution to bring its pH to 5, resulting in an amount of solid white precipitate. After vacuum filtration was employed to dry the precipitate, a crude product (white solid 1.27 g, yield 85.6%) was obtained. This product was used straight away for the following stage without undergoing additional purification. ESI-MS *m*/*z*: 306.98 $[M + H]^+$.

Synthesis of 4-(4-Iodophenoxy)-N-(2-oxooxazolidin-3-yl) Butanamide (**Y-26**)

A mixture of 4-(4-iodophenoxy) butanoic acid (1.27 g, 4.16 mmol) and 20 mL of dichloromethane in a 50 mL tri-necked container was swirled until dissolved. Then, EDCI (0.85 g, 4.44 mmol), HOBt (0.6 g, 4.44 mmol), and triethylamine (0.84 g, 9.25 mmol) were added to a bottle at 0 °C. After agitating in an ice bath for 1 h, 3-amino-2-oxazolidinone (0.43g, 4.16 mmol) was added again. After heating the mixture to 25 °C, it was stirred overnight. Following TLC, the product underwent rotational evaporation drying after being vacuum-filtered. Column chromatography (CH_2Cl_2: MeOH = 40:1). Compound **Y-26** was obtained as a white solid, 0.9g, with a yield of 55.3%. ^{1}H NMR (600 MHz, DMSO-d_6) δ 10.20 (s, 1H), 7.61–7.55 (m, 2H), 6.81–6.76 (m, 2H), 4.35 (t, *J* = 7.7 Hz, 2H), 3.97 (t, *J* = 6.4 Hz, 2H), 3.65 (t, *J* = 7.8 Hz, 2H), 3.06 (q, *J* = 7.3 Hz, 2H), 2.30 (t, *J* = 7.3 Hz, 2H), 1.95 (p, *J* = 6.8 Hz, 2H), 1.19 (t, *J* = 7.3 Hz, 3H).^{13}C NMR (151 MHz, DMSO-d_6) δ 171.36, 158.86, 157.55, 138.43, 138.43, 117.76, 117.76, 83.52, 67.18, 62.03, 46.32, 45.95, 29.84, 24.68; ESI-MS *m*/*z*: 391.0149 $[M + H]^+$.

3.1.16. Synthesis of Target Compound **Y-28**

Synthesis of Ethyl 4-Phenoxybutanoate

A 100 mL three-neck bottle contained ethyl 4-bromobutyrate (1 g, 5.1 mmol), phenol (0.66 g, 5.1 mmol), and 50 mL of acetonitrile. As cesium carbonate (3.32 g, 10.2 mmol) was added and the mixture was allowed to dissolve, the temperature of the solution rose to 50 °C. After 8 h of stirring, the mixture was allowed to cool to ambient temperature, vacuum-filtered, and then dried using a rotary evaporator. The residue was diluted with 50 mL of water, and then the solution was extracted using ethyl acetate (50 mL × 3). Anhydrous sodium sulfate was mixed with the organic layer and allowed to dry overnight. Vacuum filtering was followed by reduced pressure evaporation of the solvent to provide a crude product (light yellow oil 0.98

g, yield 92.5%) that was used straight away for the next stage without additional purification. ESI-MS *m*/*z*: 209.11 $[M + H]^+$.

Synthesis of 4-Phenoxybutanoic Acid

In a 100 mL tri-necked bottle containing ethyl 4-phenoxybutanoate (0.98 g, 4.71 mmol), 20 mL of ethanol was added, and the mixture was shaken until it dissolved. Then, 3 mL of water and NaOH (0.75 g, 18.8 mmol) were added to the bottle at 25 °C, and the solution was refluxed for 2 h. Through the use of thin-layer chromatography (TLC), the reaction was observed. After cooling the mixture to 25 °C, it was dried with a rotary evaporator, and the remaining 30 mL was diluted with water. Then, 1N hydrochloric acid was added to the solution to bring its pH to 5, resulting in an amount of solid white precipitate. After vacuum filtration was employed to dry the precipitate, a crude product (white solid 0.69 g, yield 81.6%) was obtained. This product was used straight away for the following stage without undergoing additional purification. ESI-MS *m*/*z*: 181.01 $[M + H]^+$.

Synthesis of N-(2-Oxooxazolidin-3-yl)-4-phenoxybutanamide (**Y-28**)

A mixture of 4-phenoxybutanoic acid (0.69 g, 3.85 mmol) and 20 mL of dichloromethane in a 50 mL tri-necked container was swirled until dissolved. Then, EDCI (0.85 g, 4.44 mmol), HOBt (0.6 g, 4.44 mmol), and triethylamine (0.84 g, 9.25 mmol) were added to a bottle at 0 °C. After agitating in an ice bath for 1 h, 3-amino-2-oxazolidinone (0.39 g, 3.85 mmol) was added again. After heating the mixture to 25 °C, it was stirred overnight. Following TLC, the product underwent rotational evaporation drying after being vacuum-filtered. Column chromatography (CH_2Cl_2: MeOH = 40:1). Compound **Y-28** was obtained as a yellowish solid, 0.62g, with a yield of 57.8%. ^1H NMR (600 MHz, DMSO-d_6) δ 10.18 (s, 1H), 7.31–7.25 (m, 2H), 6.94–6.89 (m, 3H), 4.36 (t, *J* = 7.8 Hz, 2H), 3.98 (t, *J* = 6.4 Hz, 2H), 3.66 (t, *J* = 7.8 Hz, 2H), 2.31 (t, *J* = 7.4 Hz, 2H), 1.96 (p, *J* = 6.8 Hz, 2H). ^{13}C NMR (151 MHz, DMSO-d_6) δ 171.43, 158.94, 129.95, 120.97, 114.91, 66.85, 62.03, 47.49, 46.32, 29.93, 24.84; ESI-MS *m*/*z*: 265.1187 $[M + H]^+$.

3.1.17. Synthesis of Target Compound **Y-29**

Synthesis of Ethyl 4-(Naphthalen-2-yloxy) Butanoate

A 100 mL three-neck bottle contained ethyl 4-bromobutyrate (1 g, 5.1 mmol), 2-Naphthol (0.74 g, 5.1 mmol), and 50 mL of acetonitrile. As cesium carbonate (3.32 g, 10.2 mmol) was added and the mixture was allowed to dissolve, the temperature of the solution rose to 50 °C. After 8 h of stirring, the mixture was allowed to cool to ambient temperature, vacuum-filtered, and then dried using a rotary evaporator. The residue was diluted with 50 mL of water, and then the solution was extracted using ethyl acetate (50 mL × 3). Anhydrous sodium sulfate was mixed with the organic layer and allowed to dry overnight. Vacuum filtering was followed by reduced pressure evaporation of the solvent to provide a crude product (light yellow oil 1.23 g, yield 93.5%) that was used straight away for the next stage without additional purification. ESI-MS *m*/*z*: 259.13 $[M + H]^+$.

Synthesis of 4-(Naphthalen-2-yloxy) Butanoic Acid

In a 100 mL tri-necked bottle containing ethyl 4-(naphthalen-2-yloxy) butanoate (1.23 g, 4.77 mmol), 20 mL of ethanol was added, and the mixture was shaken until it dissolved. Then, 3 mL of water and NaOH (0.75 g, 18.8 mmol) were added to the bottle at 25 °C, and the solution was refluxed for 2 h. Through the use of thin-layer chromatography (TLC), the reaction was observed. After cooling the mixture to 25 °C, it was dried with a rotary evaporator, and the remaining 30 mL was diluted with water. Then, 1N hydrochloric acid was added to the solution to bring its pH to 5, resulting in an amount of solid white precipitate. After vacuum filtration was employed to dry the precipitate, a crude product (white solid 0.89 g, yield

81.6%) was obtained. This product was used straight away for the following stage without undergoing additional purification. ESI-MS *m/z*: 231.09 [M + H]$^+$.

Synthesis of 4-(Naphthalen-2-yloxy)-N-(2-oxooxazolidin-3-yl) Butanamide (**Y-29**)

A mixture of 4-(naphthalen-2-yloxy) butanoic acid (0.89 g, 3.89 mmol) and 20 mL of dichloromethane in a 50 mL tri-necked container was swirled until dissolved. Then, EDCI (0.85 g, 4.44 mmol), HOBt (0.6 g, 4.44 mmol), and triethylamine (0.84 g, 9.25 mmol) were added to a bottle at 0 °C. After agitating in an ice bath for 1 h, 3-amino-2-oxazolidinone (0.40 g, 3.89 mmol) was added again. After heating the mixture to 25 °C, it was stirred overnight. Following TLC, the product underwent rotational evaporation drying after being vacuum-filtered. Column chromatography (CH_2Cl_2: MeOH = 40:1). Compound **Y-29** was obtained as a white solid, 0.63g, with a yield of 51.8%. ^{1}H NMR (600 MHz, DMSO-d_6) δ 10.21 (s, 1H), 7.84–7.77 (m, 3H), 7.45 (m, *J* = 8.2, 6.8, 1.3 Hz, 1H), 7.37–7.29 (m, 2H), 7.17 (dd, *J* = 8.9, 2.6 Hz, 1H), 4.36 (p, *J* = 7.6 Hz, 2H), 4.12 (t, *J* = 6.3 Hz, 2H), 3.67 (t, *J* = 7.8 Hz, 2H), 2.37 (t, *J* = 7.4 Hz, 2H), 2.04 (p, *J* = 6.8 Hz, 2H).^{13}C NMR (151 MHz, DMSO-d_6) δ 171.44, 157.58, 156.87, 134.76, 129.74, 128.93, 127.97, 127.14, 126.84, 124.00, 119.21, 107.18, 67.12, 62.05, 46.34, 29.98, 24.81; ESI-MS *m/z*: 315.1348 [M + H]$^+$.

3.1.18. Synthesis of Target Compound **Y-33**

Synthesis of Ethyl 2-(4-Bromophenoxy) Acetate

A 100 mL three-neck bottle contained Ethyl bromoacetate (1.03 g, 6 mmol), 4-Bromophenol (1.01 g, 6 mmol), and 50 mL of acetonitrile. As cesium carbonate (3.92 g, 12.00 mmol) was added and the mixture was allowed to dissolve, the temperature of the solution rose to 50 °C. After 8 h of stirring, the mixture was allowed to cool to ambient temperature, vacuum-filtered, and then dried using a rotary evaporator. The residue was diluted with 50 mL of water, and then the solution was extracted using ethyl acetate (50 mL × 3). Anhydrous sodium sulfate was mixed with the organic layer and allowed to dry overnight. Vacuum filtering was followed by reduced pressure evaporation of the solvent to provide a crude product (Light yellow oil 1.45 g, yield 94.5%) that was used straight away for the next stage without additional purification. ESI-MS *m/z*: 257.99 [M + H]$^+$.

Synthesis of 2-(4-Bromophenoxy) Acetic Acid

In a 100 mL tri-necked bottle containing ethyl 2-(4-bromophenoxy) acetate (1.45 g, 5.67 mmol), 20 mL of ethanol was added, and the mixture was shaken until it dissolved. Then, 3 mL of water and NaOH (0.75 g, 18.8 mmol) were added to the bottle at 25 °C, and the solution was refluxed for 2 h. Through the use of thin-layer chromatography (TLC), the reaction was observed. After cooling the mixture to 25 °C, it was dried with a rotary evaporator, and the remaining 30 mL was diluted with water. Then, 1N hydrochloric acid was added to the solution to bring its pH to 5, resulting in an amount of solid white precipitate. After vacuum filtration was employed to dry the precipitate, a crude product (white solid 1.01 g, yield 77.1%) was obtained. This product was used straight away for the following stage without undergoing additional purification. ESI-MS *m/z*: 230.98 [M + H]$^+$.

Synthesis of 2-(4-Bromophenoxy)-N-(2-oxooxazolidin-3-yl) Acetamide (**Y-33**)

A mixture of 2-(4-bromophenoxy) acetic acid (1.01 g, 4.37 mmol) and 20 mL of dichloromethane in a 50 mL tri-necked container, the mixture was swirled until dissolved. Then, EDCI (0.85 g, 4.44 mmol), HOBt (0.6 g, 4.44 mmol), and triethylamine (0.84 g, 9.25 mmol) were added to a bottle at 0 °C. After agitating in an ice bath for 1 h, 3-amino-2-oxazolidinone (0.46 g, 4.37 mmol) was added again. After heating the mixture to 25 °C, it was stirred overnight. Following TLC, the product underwent rotational evaporation drying after

being vacuum-filtered. Column chromatography (CH_2Cl_2: MeOH = 40:1). Compound **Y-33** was obtained as a white solid, 0.81g, with a yield of 58.99%. ^{1}H NMR (600 MHz, DMSO-d_6) δ 10.52 (s, 1H), 7.51–7.42 (m, 2H), 7.00–6.94 (m, 2H), 4.65 (s, 2H), 4.39 (t, *J* = 7.8 Hz, 2H), 3.68 (t, *J* = 7.8 Hz, 2H). ^{13}C NMR (151 MHz, DMSO-d_6) δ 167.27, 157.36, 157.30, 132.63, 132.63, 117.57, 117.57,113.36, 66.63, 62.17, 46.28; ESI-MS *m*/*z*: 316.9955 $[M + H]^+$.

3.1.19. Synthesis of Target Compound **Y-37**

Synthesis of Ethyl 4-(naphthalen-1-yloxy) Butanoate

A 100 mL three-neck bottle contained ethyl 4-bromobutyrate (1 g, 5.1 mmol), 1-Naphthol (0.74 g, 5.1 mmol), and 50 mL of acetonitrile. As cesium carbonate (3.32 g, 10.2 mmol) was added and the mixture was allowed to dissolve, the temperature of the solution rose to 50 °C. After 8 h of stirring, the mixture was allowed to cool to ambient temperature, vacuum-filtered, and then dried using a rotary evaporator. The residue was diluted with 50 mL of water, and then the solution was extracted using ethyl acetate (50 mL × 3). Anhydrous sodium sulfate was mixed with the organic layer and allowed to dry overnight. Vacuum filtering was followed by reduced pressure evaporation of the solvent to provide a crude product (light yellow oil 1.21 g, yield 92.5%) that was used straight away for the next stage without additional purification. ESI-MS *m*/*z*: 259.13 $[M + H]^+$.

Synthesis of 4-(Naphthalen-1-yloxy) Butanoic Acid

In a 100 mL tri-necked bottle containing ethyl 4-(naphthalen-1-yloxy) butanoate (1.21 g, 4.72 mmol), 20 mL of ethanol was added, and the mixture was shaken until it dissolved. Then, 3 mL of water and NaOH (0.75 g, 18.8 mmol) were added to the bottle at 25 °C, and the solution was refluxed for 2 h. Through the use of thin-layer chromatography (TLC), the reaction was observed. After cooling the mixture to 25 °C, it was dried with a rotary evaporator, and the remaining 30 mL was diluted with water. Then, 1N hydrochloric acid was added to the solution to bring its pH to 5, resulting in an amount of solid white precipitate. After vacuum filtration was employed to dry the precipitate, a crude product (white solid 0.91 g, yield 83.6%) was obtained. This product was used straight away for the following stage without undergoing additional purification. ESI-MS *m*/*z*: 231.09 $[M + H]^+$.

Synthesis of 4-(Naphthalen-1-yloxy)-N-(2-oxooxazolidin-3-yl) Butanamide (**Y-37**)

A mixture of 4-(naphthalen-1-yloxy) butanoic acid (0.91 g, 3.94 mmol) and 20 mL of dichloromethane in a 50 mL tri-necked container was swirled until dissolved. Then, EDCI (0.85 g, 4.44 mmol), HOBt (0.6 g, 4.44 mmol), and triethylamine (0.84 g, 9.25 mmol) were added to a bottle at 0 °C. After agitating in an ice bath for 1 h, 3-amino-2-oxazolidinone (0.40 g, 3.94 mmol) was added again. After heating the mixture to 25 °C, it was stirred overnight. Following TLC, the product underwent rotational evaporation drying after being vacuum-filtered. Column chromatography (CH_2Cl_2: MeOH = 40:1). Compound **Y-37** was obtained as a white solid, 0.66g, with a yield of 53.8%. ^{1}H NMR (600 MHz, DMSO-d_6) δ 10.23 (s, 1H), 8.20 (dd, *J* = 7.7, 1.7 Hz, 1H), 7.87 (dd, *J* = 7.5, 1.7 Hz, 1H), 7.56–7.44 (m, 3H), 7.41 (t, *J* = 7.9 Hz, 1H), 6.95 (dd, *J* = 7.7, 1.0 Hz, 1H), 4.37 (t, *J* = 7.8 Hz, 2H), 4.18 (t, *J* = 6.2 Hz, 2H), 3.68 (t, *J* = 7.8 Hz, 2H), 2.45 (t, *J* = 7.4 Hz, 2H), 2.16–2.07 (m, 2H). ^{13}C NMR (151 MHz, DMSO-d_6) δ 157.57, 154.41, 134.50, 127.91, 126.90, 126.72, 125.71, 125.42, 122.07, 120.36, 105.58, 67.35, 62.05, 46.36, 30.15, 24.93; ESI-MS *m*/*z*: 315.1341 $[M + H]^+$.

3.1.20. Synthesis of Target Compound **Y-38**

Synthesis of Ethyl 4-(4-Isopropylphenoxy) Butanoate

A 100 mL three-neck bottle contained ethyl 4-bromobutyrate (1 g, 5.1 mmol), 4-Isopropylphenol (0.69 g, 5.1 mmol), and 50 mL of acetonitrile. As cesium carbonate (3.32 g, 10.2 mmol) was added and the mixture was allowed to dissolve, the temperature of the solution rose to 50 °C. After 8 h of stirring, the mixture was allowed to cool to ambient temperature, vacuum-filtered, and then dried using a rotary evaporator. The residue was diluted with 50 mL of water, and then the solution was extracted using ethyl acetate (50 mL × 3). Anhydrous sodium sulfate was mixed with the organic layer and allowed to dry overnight. Vacuum filtering was followed by reduced pressure evaporation of the solvent to provide a crude product (light yellow oil 1.16 g, yield 90.5%) that was used straight away for the next stage without additional purification. ESI-MS *m*/*z*: 251.16 $[M + H]^+$.

Synthesis of 4-(4-Isopropylphenoxy) Butanoic Acid

In a 100 mL tri-necked bottle containing ethyl 4-(4-isopropylphenoxy) butanoate (1.16 g, 4.62 mmol), 20 mL of ethanol was added, and the mixture was shaken until it dissolved. Then, 3 mL of water and NaOH (0.75 g, 18.8 mmol) were added to the bottle at 25 °C, and the solution was refluxed for 2 h. Through the use of thin-layer chromatography (TLC), the reaction was observed. After cooling the mixture to 25 °C, it was dried with a rotary evaporator, and the remaining 30 mL was diluted with water. Then, 1N hydrochloric acid was added to the solution to bring its pH to 5, resulting in an amount of solid white precipitate. After vacuum filtration was employed to dry the precipitate, a crude product (white solid 0.85 g, yield 82.6%) was obtained. This product was used straight away for the following stage without undergoing additional purification. ESI-MS *m*/*z*: 223.13 $[M + H]^+$.

Synthesis of 4-(4-Isopropylphenoxy)-N-(2-oxooxazolidin-3-yl) Butanamide (**Y-38**)

A mixture of 4-(4-isopropylphenoxy) butanoic acid (0.85 g, 3.81 mmol) and 20 mL of dichloromethane in a 50 mL tri-necked container was swirled until dissolved. Then, EDCI (0.85 g, 4.44 mmol), HOBt (0.6 g, 4.44 mmol), and triethylamine (0.84 g, 9.25 mmol) were added to a bottle at 0 °C. After agitating in an ice bath for 1 h, 3-amino-2-oxazolidinone (0.39 g, 3.81 mmol) was added again. After heating the mixture to 25 °C, it was stirred overnight. Following TLC, the product underwent rotational evaporation drying after being vacuum-filtered. Column chromatography (CH_2Cl_2: MeOH = 40:1). Compound **Y-38** was obtained as a white solid, 0.62g, with a yield of 53.8%. ^{1}H NMR (600 MHz, DMSO-d_6) δ 10.17 (s, 1H), 7.15–7.10 (m, 2H), 6.86–6.81 (m, 2H), 4.35 (t, *J* = 7.7 Hz, 2H), 3.94 (t, *J* = 6.4 Hz, 2H), 3.66 (t, *J* = 7.7 Hz, 2H), 2.82 (t, *J* = 6.9 Hz, 1H), 2.30 (t, *J* = 7.4 Hz, 2H), 1.94 (p, *J* = 6.8 Hz, 2H), 1.16 (d, *J* = 6.9 Hz, 6H). ^{13}C NMR (151 MHz, DMSO-d_6) δ 157.56, 157.04, 140.86, 127.58, 127.58,114.74, 114.74, 66.94, 62.03, 46.32, 33.04, 29.95, 24.89, 24.60; ESI-MS *m*/*z*: 307.1653 $[M + H]^+$.

3.1.21. Synthesis of Target Compound **Y-39**

Synthesis of Ethyl 4-((4-bromophenyl) thio) Butanoate

A 100 mL three-neck bottle contained ethyl 4-bromobutyrate (1 g, 5.1 mmol), 4-Bromothiophenol (0.96g, 5.1 mmol), and 50 mL of acetonitrile. As cesium carbonate (3.32 g, 10.2 mmol) was added and the mixture was allowed to dissolve, the temperature of the solution rose to 50 °C. After 8 h of stirring, the mixture was allowed to cool to ambient temperature, vacuum-filtered, and then dried using a rotary evaporator. The residue was diluted with 50 mL of water, and then the solution was extracted using ethyl acetate (50 mL × 3). Anhydrous sodium sulfate was mixed with the organic layer and allowed to dry overnight. Vacuum filtering was followed by reduced pressure evaporation of the solvent to provide a crude product (light yellow oil 1.43 g, yield 92.5%) that was used straight away for the next stage without additional purification. ESI-MS *m*/*z*: 303.01 $[M + H]^+$.

Synthesis of 4-((4-Bromophenyl) thio) Butanoic Acid

In a 100 mL tri-necked bottle containing ethyl ethyl 4-((4-bromophenyl) thio) butanoate (1.43 g, 4.72 mmol), 20 mL of ethanol was added, and the mixture was shaken until it dissolved. Then, 3 mL of water and NaOH (0.75 g, 18.8 mmol) were added to the bottle at 25 °C, and the solution was refluxed for 2 h. Through the use of thin-layer chromatography (TLC), the reaction was observed. After cooling the mixture to 25 °C, it was dried with a rotary evaporator, and the remaining 30 mL was diluted with water. Then, 1N hydrochloric acid was added to the solution to bring its pH to 5, resulting in an amount of solid white precipitate. After vacuum filtration was employed to dry the precipitate, a crude product (white solid 1.09 g, yield 84.6%) was obtained. This product was used straight away for the following stage without undergoing additional purification. ESI-MS *m*/*z*: 274.97 $[M + H]^+$.

Synthesis of 4-((4-Bromophenyl) thio)-N-(2-oxooxazolidin-3-yl) Butanamide (**Y-39**)

A mixture of 4-((4-bromophenyl) thio) butanoic acid (1.09g, 3.99 mmol) and 20 mL of dichloromethane in a 50 mL tri-necked container was swirled until dissolved. Then, EDCI (0.85 g, 4.44 mmol), HOBt (0.6 g, 4.44 mmol), and triethylamine (0.84 g, 9.25 mmol) were added to a bottle at 0 °C. After agitating in an ice bath for 1 h, 3-amino-2-oxazolidinone (0.41g, 3.99 mmol) was added again. After heating the mixture to 25 °C, it was stirred overnight. Following TLC, the product underwent rotational evaporation drying after being vacuum-filtered. Column chromatography (CH_2Cl_2: MeOH = 40:1). Compound **Y-39** was obtained as a white solid, 0.74g, with a yield of 51.8%.^1H NMR (600 MHz, DMSO-d_6) δ 10.17 (s, 1H), 7.52–7.47 (m, 2H), 7.30–7.25 (m, 2H), 4.36 (t, *J* = 7.7 Hz, 2H), 3.65 (t, *J* = 7.8 Hz, 2H), 2.99 (t, *J* = 7.3 Hz, 2H), 2.28 (t, *J* = 7.2 Hz, 2H), 1.81 (p, *J* = 7.2 Hz, 2H).^{13}C NMR (151 MHz, DMSO-d_6) δ 171.20, 157.57, 136.22, 132.30, 132.30, 130.21, 130.21, 62.05, 46.32, 40.54, 32.12, 31.47, 24.70; ESI-MS *m*/*z*: 361.0042 $[M + H]^+$.

3.1.22. Synthesis of Target Compound **Y-40**

Synthesis of Ethyl 4-(2,4-Dimethylphenoxy) Butanoate

A 100 mL three-neck bottle contained ethyl 4-bromobutyrate (1 g, 5.1 mmol), 2,4-Dimethylphenol (0.62 g, 5.1 mmol), and 50 mL of acetonitrile. As cesium carbonate (3.32 g, 10.2 mmol) was added and the mixture was allowed to dissolve, the temperature of the solution rose to 50 °C. After 8 h of stirring, the mixture was allowed to cool to ambient temperature, vacuum-filtered, and then dried using a rotary evaporator. The residue was diluted with 50 mL of water, and then the solution was extracted using ethyl acetate (50 mL × 3). Anhydrous sodium sulfate was mixed with the organic layer and allowed to dry overnight. Vacuum filtering was followed by reduced pressure evaporation of the solvent to provide a crude product (light yellow oil 1.08 g, yield 89.5%) that was used straight away for the next stage without additional purification. ESI-MS *m*/*z*: 237.14 $[M + H]^+$.

Synthesis of 4-(2,4-Dimethylphenoxy) Butanoic Acid

In a 100 mL tri-necked bottle containing ethyl 4-(2,4-dimethylphenoxy) butanoate (1.08 g, 4.59 mmol), 20 mL of ethanol was added, and the mixture was shaken until it dissolved. Then, 3 mL of water and NaOH (0.75 g, 18.8 mmol) were added to the bottle at 25 °C, and the solution was refluxed for 2 h. Through the use of thin-layer chromatography (TLC), the reaction was observed. After cooling the mixture to 25 °C, it was dried with a rotary evaporator, and the remaining 30 mL was diluted with water. Then, 1N hydrochloric acid was added to the solution to bring its pH to 5, resulting in an amount of solid white precipitate. After vacuum filtration was employed to dry the precipitate, a crude product

(white solid 0.77 g, yield 80.6%) was obtained. This product was used straight away for the following stage without undergoing additional purification. ESI-MS *m*/*z*: 209.11 $[M + H]^+$.

Synthesis of 4-(2,4-Dimethylphenoxy)-N-(2-oxooxazolidin-3-yl) Butanamide (**Y-40**)

A mixture of 4-(2,4-dimethylphenoxy) butanoic acid (0.86 g, 3.70 mmol) and 20 mL of dichloromethane in a 50 mL tri-necked container was swirled until dissolved. Then, EDCI (0.85 g, 4.44 mmol), HOBt (0.6 g, 4.44 mmol), and triethylamine (0.84 g, 9.25 mmol) were added to a bottle at 0 °C. After agitating in an ice bath for 1 h, 3-amino-2-oxazolidinone (0.38 g, 3.70 mmol) was added again. After heating the mixture to 25 °C, it was stirred overnight. Following TLC, the product underwent rotational evaporation drying after being vacuum-filtered. Column chromatography (CH_2Cl_2: MeOH = 40:1). Compound **Y-40** was obtained as a white solid, 0.57g, with a yield of 52.8%.^{1}H NMR (600 MHz, DMSO-d_6) δ 10.17 (s, 1H), 7.00 (d, *J* = 8.2 Hz, 1H), 6.72 (d, *J* = 2.7 Hz, 1H), 6.63 (dd, *J* = 8.2, 2.7 Hz, 1H), 4.38–4.32 (m, 2H), 3.95–3.90 (m, 2H), 3.66 (t, *J* = 7.8 Hz, 2H), 2.29 (t, *J* = 7.4 Hz, 2H), 2.17 (s, 3H), 2.12 (s, 3H), 1.93 (p, *J* = 6.9 Hz, 2H).^{13}C NMR (151 MHz, DMSO-d_6) δ 171.44, 157.56, 157.04, 137.68, 130.62, 128.37, 116.34, 111.91, 66.85, 62.02, 46.32, 29.93, 24.90, 20.09, 18.88; ESI-MS *m*/*z*: 293.1501 $[M + H]^+$.

3.1.23. Synthesis of Target Compound **Y-41**

Synthesis of Ethyl 4-(4-Isobutylphenoxy) Butanoate

A 100 mL three-neck bottle contained ethyl 4-bromobutyrate (1 g, 5.1 mmol), (4-isobutylphenyl) methanol (0.77 g, 5.1 mmol), and 50 mL of acetonitrile. As cesium carbonate (3.32 g, 10.2 mmol) was added and the mixture was allowed to dissolve, the temperature of the solution rose to 50 °C. After 8 h of stirring, the mixture was allowed to cool to ambient temperature, vacuum-filtered, and then dried using a rotary evaporator. The residue was diluted with 50 mL of water, and then the solution was extracted using ethyl acetate (50 mL × 3). Anhydrous sodium sulfate was mixed with the organic layer and allowed to dry overnight. Vacuum filtering was followed by reduced pressure evaporation of the solvent to provide a crude product (light yellow oil 1.2 g, yield 91.01%) that was used straight away for the next stage without additional purification. ESI-MS *m*/*z*: 265.18 $[M + H]^+$.

Synthesis of 4-(4-Isobutylphenoxy) Butanoic Acid

In a 100 mL tri-necked bottle containing ethyl 4-(isoquinolin-7-yloxy) butanoate (1.29 g, 4.87 mmol), 20 mL of ethanol was added, and the mixture was shaken until it dissolved. Then, 3 mL of water and NaOH (0.75 g, 18.8 mmol) were added to the bottle at 25 °C, and the solution was refluxed for 2 h. Through the use of thin-layer chromatography (TLC), the reaction was observed. After cooling the mixture to 25 °C, it was dried with a rotary evaporator, and the remaining 30 mL was diluted with water. Then, 1N hydrochloric acid was added to the solution to bring its pH to 5, resulting in an amount of solid white precipitate. After vacuum filtration was employed to dry the precipitate, a crude product (white solid 0.96 g, yield 83.2%) was obtained. This product was used straight away for the following stage without undergoing additional purification. ESI-MS *m*/*z*: 237.14 $[M + H]^+$.

Synthesis of 4-(4-Isobutylphenoxy)-N-(2-oxooxazolidin-3-yl) Butanamide (**Y-41**)

A mixture of 4-(4-isobutylphenoxy) butanoic acid (0.96 g, 4.05 mmol) and 20 mL of dichloromethane in a 50 mL tri-necked container was swirled until dissolved. Then, EDCI (0.85 g, 4.44 mmol), HOBt (0.6 g, 4.44 mmol), and triethylamine (0.84 g, 9.25 mmol) were added to a bottle at 0 °C. After agitating in an ice bath for 1 h, 3-amino-2-oxazolidinone (0.41 g, 4.05 mmol) was added again. After heating the mixture to 25 °C, it was stirred overnight. Following TLC, the product underwent rotational evaporation drying after being vacuum-filtered. Column chromatography (CH_2Cl_2: MeOH = 40:1). Compound **Y-41** was obtained as a

white solid, 0.67g, with a yield of 55.1%.^{1}H NMR (600 MHz, DMSO-d_6) δ 10.18 (s, 1H), 7.11–7.06 (m, 2H), 6.86–6.81 (m, 2H), 4.36 (t, *J* = 7.8 Hz, 2H), 3.95 (t, *J* = 6.4 Hz, 2H), 3.66 (t, *J* = 7.8 Hz, 2H), 2.30 (t, *J* = 7.4 Hz, 2H), 1.94 (p, *J* = 6.8 Hz, 2H), 1.57–1.43 (m, 2H), 1.15 (d, *J* = 6.9 Hz, 3H), 0.74 (t, *J* = 7.3 Hz, 3H).^{13}C NMR (151 MHz, DMSO-d_6) δ 171.44, 157.56, 157.07, 139.51, 128.18, 128.18, 114.69, 114.69,66.91, 62.03, 46.32, 31.19, 29.96, 24.92, 22.46, 22.46; ESI-MS *m*/*z*: 321.1808 $[M + H]^+$.

3.1.24. Synthesis of 3-Hydrazino-1-propanol

Here, 40g of sodium hydroxide and 250g of hydrazine hydrate were added to a 100 mL three-neck flask at 25 °C, and the mixture was subsequently heated to 95 °C. Then, 94 g of 3-chloropropanol was added at an internal temperature of 95 to 100 °C. The reaction was carried out at 95–100 °C for 2 h; the mixture was concentrated under reduced pressure, filtered, and the solids were washed with ethanol. Then, 49.6 g of a colorless oil liquid was obtained at 102–104 °C (0.6 mmHg). ESI-MS *m*/*z*: 91.06 $[M + H]^+$.

3.1.25. Synthesis of 3-amino-tetrahydro-l,3-oxazin-2-one

First, a 250 mL three-neck container was used to hold 3-Hydrazino-1-propanol (18.01 g, 0.20 mol), diethyl carbonate (30.68 g, 0.26 mol), and sodium methoxide (4.08 g, 0.06 mol). For four hours, the solution was heated under reflux. The solid precipitate was filtered, and the solids were dried after cooling to room temperature. When dry, the solvent was evacuated. The products underwent column chromatography (methanol/dichloromethane *V*/*V* 1:40). Ethanol was used to recrystallize the dry solid and white solid. With a weight of 19.22 g, the white solid yielded 62.81% ESI-MS *m*/*z*: 116.01 $[M + H]^+$.

3.1.26. Synthesis of Target Compound **Y-4**

Synthesis of Ethyl 5-(4-bromophenoxy) Pentanoate

A 100 mL three-neck bottle contained ethyl Ethyl 5-bromovalerate (1.02 g, 4.7 mmol), 4-Bromophenol (0.83 g, 4.7 mmol), and 50 mL of acetonitrile. As cesium carbonate (3.32 g, 10.2 mmol) was added and the mixture was allowed to dissolve, the temperature of the solution rose to 50 °C. After 8 h of stirring, the mixture was allowed to cool to ambient temperature, vacuum-filtered, and then dried using a rotary evaporator. The residue was diluted with 50 mL of water, and then the solution was extracted using ethyl acetate (50 mL × 3). Anhydrous sodium sulfate was mixed with the organic layer and allowed to dry overnight. Vacuum filtering was followed by reduced pressure evaporation of the solvent to provide a crude product (light yellow oil 1.29 g, yield 91.3%) that was used straight away for the next stage without additional purification. ESI-MS *m*/*z*: 301.04 $[M + H]^+$.

Synthesis of 5-(4-Bromophenoxy) Pentanoic Acid

In a 100 mL tri-necked bottle containing ethyl 5-(4-bromophenoxy) pentanoate (1.29 g, 4.29 mmol), 20 mL of ethanol was added, and the mixture was shaken until it dissolved. Then, 3 mL of water and NaOH (0.75 g, 18.8 mmol) were added to the bottle at 25 °C, and the solution was refluxed for 2 h. Through the use of thin-layer chromatography (TLC), the reaction was observed. After cooling the mixture to 25 °C, it was dried with a rotary evaporator, and the remaining 30 mL was diluted with water. Then, 1N hydrochloric acid was added to the solution to bring its pH to 5, resulting in an amount of solid white precipitate. After vacuum filtration was employed to dry the precipitate, a crude product (white solid 0.84 g, yield 72.3%) was obtained. This product was used straight away for the following stage without undergoing additional purification. ESI-MS *m*/*z*: 273.00 $[M + H]^+$.

Synthesis of 5-(4-Bromophenoxy)-N-(2-oxo-1,3-oxazinan-3-yl) Pentanamide (**Y-4**)

A mixture of 5-(4-bromophenoxy) pentanoic acid (0.84 g, 3.10 mmol) and 20 mL of dichloromethane in a 50 mL tri-necked container was swirled until dissolved. Then, EDCI (0.85 g, 4.44 mmol), HOBt (0.6 g, 4.44 mmol), and triethylamine (0.84 g, 9.25 mmol) were added to a bottle at 0 °C. After agitating in an ice bath for 1 h, 3-amino-tetrahydro-l,3-oxazin-2-one (0.32 g, 3.28 mmol) was added again. After heating the mixture to 25 °C, it was stirred overnight. Following TLC, the product underwent rotational evaporation drying after being vacuum-filtered. Column chromatography (CH_2Cl_2: MeOH = 40:1). Compound **Y-4** was obtained as a white solid, 0.51g, with a yield of 58.1%.^{1}H NMR (600 MHz, DMSO-d_6) δ 10.15 (s, 1H), 7.46–7.40 (m, 2H), 6.93–6.87 (m, 2H), 4.22 (t, J = 5.2 Hz, 2H), 3.95 (t, J = 6.3 Hz, 2H), 3.42 (t, J = 6.2 Hz, 2H), 2.17 (t, J = 7.2 Hz, 2H), 2.07–2.00 (m, 2H), 1.76–1.61 (m, 4H).^{13}C NMR (151 MHz, DMSO-d_6) δ 171.41, 158.38, 152.98, 132.55, 117.22, 112.21, 67.86, 67.16, 50.47, 49.30, 33.08, 28.55, 28.36, 22.78, 21.93; ESI-MS m/z: 371.0605 [M + H]$^+$.

3.1.27. Synthesis of Target Compound **Y-6**

Synthesis of Ethyl 4-(4-Bromophenoxy) Butanoate

A 100 mL three-neck bottle contained ethyl 4-bromobutyrate (1.02 g, 5.1 mmol), 4-Bromophenol (0.88 g, 5.1 mmol), and 50 mL of acetonitrile. As cesium carbonate (3.32 g, 10.2 mmol) was added and the mixture was allowed to dissolve, the temperature of the solution rose to 50 °C. After 8 h of stirring, the mixture was allowed to cool to ambient temperature, vacuum-filtered, and then dried using a rotary evaporator. The residue was diluted with 50 mL of water, and then the solution was extracted using ethyl acetate (50 mL × 3). Anhydrous sodium sulfate was mixed with the organic layer and allowed to dry overnight. Vacuum filtering was followed by reduced pressure evaporation of the solvent to provide a crude product (light yellow oil 1.31 g, yield 89.8%) that was used straight away for the next stage without additional purification. ESI-MS m/z: 286.02 [M + H]$^+$.

Synthesis of 4-(4-Bromophenoxy) Butanoic Acid

In a 100 mL tri-necked bottle containing ethyl 4-(4-bromophenoxy) butanoate (1.31 g, 4.58 mmol), 20 mL of ethanol was added, and the mixture was shaken until it dissolved. Then, 3 mL of water and NaOH (0.75 g, 18.8 mmol) were added to the bottle at 25 °C, and the solution was refluxed for 2 h. Through the use of thin-layer chromatography (TLC), the reaction was observed. After cooling the mixture to 25 °C, it was dried with a rotary evaporator, and the remaining 30 mL was diluted with water. Then, 1N hydrochloric acid was added to the solution to bring its pH to 5, resulting in an amount of solid white precipitate. After vacuum filtration was employed to dry the precipitate, a crude product (white solid 0.84 g, yield 71.2%) was obtained. This product was used straight away for the following stage without undergoing additional purification. ESI-MS m/z: 258.99 [M + H]$^+$.

Synthesis of 4-(4-Bromophenoxy)-N-(2-oxo-1,3-oxazinan-3-yl) Butanamide (**Y-6**)

A mixture of 4-(4-bromophenoxy) butanoic acid (0.84 g, 3.26 mmol) and 20 mL of dichloromethane in a 50 mL tri-necked container was swirled until dissolved. Then, EDCI (0.85 g, 4.44 mmol), HOBt (0.6 g, 4.44 mmol), and triethylamine (0.84 g, 9.25 mmol) were added to a bottle at 0 °C. After agitating in an ice bath for 1 h, 3-amino-tetrahydro-l,3-oxazin-2-one (0.38 g, 3.26 mmol) was added again. After heating the mixture to 25 °C, it was stirred overnight. Following TLC, the product underwent rotational evaporation drying after being vacuum-filtered. Column chromatography (CH_2Cl_2: MeOH = 40:1). Compound **Y-6** was obtained as a white solid, 0.51g, with a yield of 53.7%. ^{1}H NMR (600 MHz, DMSO-d_6) δ 10.23 (s, 1H), 7.46–7.41 (m, 2H), 6.94–6.87 (m, 2H), 4.15 (s, 2H), 3.99 (t, J = 6.4 Hz, 2H), 3.90 (dd, J = 6.0, 4.4 Hz, 2H), 3.48 (dd, J = 5.9, 4.5 Hz, 2H), 2.31 (t, J = 7.4 Hz, 2H), 1.96 (p, J = 6.8 Hz, 2H).^{13}C

NMR (151 MHz, DMSO-d_6) δ 170.92, 166.02, 158.28, 132.59, 117.25, 112.33, 68.38, 67.37, 64.26, 50.51, 29.92, 24.83; ESI-MS *m*/*z*: 359.0430 $[M + H]^+$.

3.1.28. Synthesis of Target Compound **Y-10**

Synthesis of Ethyl 2-(4-Bromophenoxy) Acetate

A 100 mL three-neck bottle contained Ethyl bromoacetate (1.02 g, 6 mmol), 4-Bromophenol (1.03 g, 6 mmol), and 50 mL of acetonitrile. As cesium carbonate (3.92 g, 12.00 mmol) was added and the mixture was allowed to dissolve, the temperature of the solution rose to 50 °C. After 8 h of stirring, the mixture was allowed to cool to ambient temperature, vacuum-filtered, and then dried using a rotary evaporator. The residue was diluted with 50 mL of water, and then the solution was extracted using ethyl acetate (50 mL × 3). Anhydrous sodium sulfate was mixed with the organic layer and allowed to dry overnight. Vacuum filtering was followed by reduced pressure evaporation of the solvent to provide a crude product (light yellow oil 1.4 g, yield 91.3%) that was used straight away for the next stage without additional purification. ESI-MS *m*/*z*: 257.99 $[M + H]^+$.

Synthesis of 2-(4-Bromophenoxy) Acetic Acid

In a 100 mL tri-necked bottle containing ethyl 2-(4-bromophenoxy) acetate (1.4 g,5.47 mmol), 20 mL of ethanol was added, and the mixture was shaken until it dissolved. Then, 3 mL of water and NaOH (0.75 g, 18.8 mmol) were added to the bottle at 25 °C, and the solution was refluxed for 2 h. Through the use of thin-layer chromatography (TLC), the reaction was observed. After cooling the mixture to 25 °C, it was dried with a rotary evaporator, and the remaining 30 mL was diluted with water. Then, 1N hydrochloric acid was added to the solution to bring its pH to 5, resulting in an amount of solid white precipitate. After vacuum filtration was employed to dry the precipitate, a crude product (white solid 0.87 g, yield 70.3%) was obtained. This product was used straight away for the following stage without undergoing additional purification. ESI-MS *m*/*z*: 229.96 $[M + H]^+$.

Synthesis of 2-(4-Bromophenoxy)-N-(2-oxo-1,3-oxazinan-3-yl) Acetamide (**Y-10**)

A mixture of 2-(4-bromophenoxy) acetic acid (0.87 g, 3.85 mmol) and 20 mL of dichloromethane in a 50 mL tri-necked container was swirled until dissolved. Then, EDCI (0.85 g, 4.44 mmol), HOBt (0.6 g, 4.44 mmol), and triethylamine (0.84 g, 9.25 mmol) were added to a bottle at 0 °C. After agitating in an ice bath for 1 h, 3-amino-tetrahydro-l,3-oxazin-2-one (0.45 g, 3.85 mmol) was added again. After heating the mixture to 25 °C, it was stirred overnight. Following TLC, the product underwent rotational evaporation drying after being vacuum-filtered. Column chromatography (CH_2Cl_2: MeOH = 40:1). Compound **Y-10** was obtained as a white solid, 0.69g, with a yield of 55.1%.^{1}H NMR (600 MHz, DMSO-d_6) δ 10.51 (s, 1H), 7.50–7.41 (m, 2H), 6.99–6.93 (m, 2H), 4.61 (s, 2H), 4.24 (t, *J* = 5.2 Hz, 2H), 3.45 (t, *J* = 6.2 Hz, 2H), 2.09–2.02 (m, 2H). ^{13}C NMR (151 MHz, DMSO) δ 166.87, 157.36, 152.73, 132.59, 132.43, 117.57, 117.27, 113.28, 67.27, 66.66, 49.28, 22.76; ESI-MS *m*/*z*: 329.0135 $[M + H]^+$.

3.1.29. Synthesis of Target Compound **Y-20**

Synthesis of Ethyl 2-(4-chlorophenoxy) Acetate

A 100 mL three-neck bottle contained Ethyl bromoacetate (1.02 g, 6 mmol), 4-Chlorophenol (0.77 g, 6 mmol), and 50 mL of acetonitrile. As cesium carbonate (3.92 g, 12.00 mmol) was added and the mixture was allowed to dissolve, the temperature of the solution rose to 50 °C. After 8 h of stirring, the mixture was allowed to cool to ambient temperature, vacuum-filtered, and then dried using a rotary evaporator. The residue was diluted with 50 mL of water, and then the solution was extracted using ethyl acetate (50 mL × 3). Anhydrous sodium

sulfate was mixed with the organic layer and allowed to dry overnight. Vacuum filtering was followed by reduced pressure evaporation of the solvent to provide a crude product (light yellow oil 1.2 g, yield 93.8%) that was used straight away for the next stage without additional purification. ESI-MS *m*/*z*: 215.04 $[M + H]^+$.

Synthesis of 2-(4-Chlorophenoxy) Acetic Acid

In a 100 mL tri-necked bottle containing ethyl 2-(4-chlorophenoxy) acetate (1.05 g, 5.62 mmol), 20 mL of ethanol was added, and the mixture was shaken until it dissolved. Then, 3 mL of water and NaOH (0.75 g, 18.8 mmol) were added to the bottle at 25 °C, and the solution was refluxed for 2 h. Through the use of thin-layer chromatography (TLC), the reaction was observed. After cooling the mixture to 25 °C, it was dried with a rotary evaporator, and the remaining 30 mL was diluted with water. Then, 1N hydrochloric acid was added to the solution to bring its pH to 5, resulting in an amount of solid white precipitate. After vacuum filtration was employed to dry the precipitate, a crude product (white solid 0.75 g, yield 72.5%) was obtained. This product was used straight away for the following stage without undergoing additional purification. ESI-MS *m*/*z*: 187.01 $[M + H]^+$.

Synthesis of 2-(4-Chlorophenoxy)-N-(2-oxo-1,3-oxazinan-3-yl) Acetamide (**Y-20**)

A mixture of 2-(4-chlorophenoxy) acetic acid (0.75 g, 4.08 mmol) and 20 mL of dichloromethane in a 50 mL tri-necked container was swirled until dissolved. Then, EDCI (0.85 g, 4.44 mmol), HOBt (0.6 g, 4.44 mmol), and triethylamine (0.84 g, 9.25 mmol) were added to a bottle at 0 °C. After agitating in an ice bath for 1 h, 3-amino-tetrahydro-l,3-oxazin-2-one (0.47 g, 3.28 mmol) was added again. After heating the mixture to 25 °C, it was stirred overnight. Following TLC, the product underwent rotational evaporation drying after being vacuum-filtered. Column chromatography (CH_2Cl_2: MeOH = 40:1). Compound **Y-20** was obtained as a white solid, 0.74g, with a yield of 57.8%.^{1}H NMR (600 MHz, DMSO-d_6) δ 10.51 (s, 1H), 7.38–7.29 (m, 2H), 7.04–6.98 (m, 2H), 4.61 (s, 2H), 4.24 (t, *J* = 5.3 Hz, 2H), 3.45 (t, *J* = 6.2 Hz, 2H), 2.09–2.01 (m, 2H). ^{13}C NMR (151 MHz, DMSO-d_6) δ 166.90, 156.91, 152.74, 129.70, 129.54, 125.54, 117.05, 67.27, 66.75, 49.28, 22.76; ESI-MS *m*/*z*: 285.0637 $[M + H]^+$.

3.1.30. Synthesis of 4-Ethyl-N-(2-oxo-1,3-oxazinan-3-yl) Benzamide **Y-22**

A mixture of 4-Ethylbenzoic acid (0.5 g, 3.36 mmol) and 20 mL of dichloromethane in a 50 mL tri-necked container was swirled until dissolved. Then, EDCI (0.85 g, 4.44 mmol), HOBt (0.6 g, 4.44 mmol), and triethylamine (0.84 g, 9.25 mmol) were added to a bottle at 0 °C. After agitating in an ice bath for 1 h, 3-amino-tetrahydro-l,3-oxazin-2-one (0.39g, 3.36 mmol) was added again. After heating the mixture to 25 °C, it was stirred overnight. Following TLC, the product underwent rotational evaporation drying after being vacuum-filtered. Column chromatography (CH_2Cl_2: MeOH = 40:1). White solid 0.58 g. Yield of 60.33%. ^{1}H NMR (600 MHz, DMSO-d_6) δ 10.75 (s, 1H), 7.81–7.77 (m, 2H), 7.34 (d, *J* = 8.1 Hz, 2H), 4.28 (t, *J* = 5.3 Hz, 2H), 3.54 (t, *J* = 6.2 Hz, 2H), 2.67 (q, *J* = 7.6 Hz, 2H), 2.14–2.07 (m, 2H), 1.19 (t, *J* = 7.6 Hz, 3H).^{13}C NMR (151 MHz, DMSO-d_6) δ 165.47, 153.13, 148.76, 129.98, 128.35, 128.05, 67.29, 49.42, 40.54, 28.55, 22.89, 15.76; ESI-MS *m*/*z*: 249.1229 $[M + H]^+$.

3.1.31. Synthesis of 4-Methyl-N-(2-oxo-1,3-oxazinan-3-yl) Benzamide **Y-23**

A mixture of p-Toluic acid (0.5 g, 3.67 mmol) and 20 mL of dichloromethane in a 50 mL tri-necked container was swirled until dissolved. Then, EDCI (0.85 g, 4.44 mmol), HOBt (0.6 g, 4.44 mmol), and triethylamine (0.84 g, 9.25 mmol) were added to a bottle at 0 °C. After agitating in an ice bath for 1 h, 3-amino-tetrahydro-l,3-oxazin-2-one (0.31g, 3.67 mmol) was added again. After heating the mixture to 25 °C, it was stirred overnight. Following TLC, the product underwent rotational evaporation drying after being vacuum-filtered. Column

chromatography (CH_2Cl_2: MeOH = 40:1). White solid 0.51 g. Yield of 59.33%. ^{1}H NMR (600 MHz, DMSO-d_6) δ 10.75 (s, 1H), 7.77 (d, *J* = 7.9 Hz, 2H), 7.31 (d, *J* = 7.9 Hz, 2H), 4.28 (t, *J* = 5.3 Hz, 2H), 3.54 (t, *J* = 6.2 Hz, 2H), 2.37 (s, 3H), 2.10 (p, *J* = 5.8 Hz, 2H).^{13}C NMR (151 MHz, DMSO-d_6) δ 165.43, 153.14, 142.62, 129.71, 129.51, 127.95, 67.29, 49.42, 46.12, 22.88, 21.50; ESI-MS *m*/*z*: 235.1093 $[M + H]^+$.

3.1.32. Synthesis of Target Compound **Y-27**

Synthesis of Ethyl 4-(4-(Trifluoromethyl)phenoxy) Butanoate

A 100 mL three-neck bottle contained ethyl 4-bromobutyrate (1 g, 5.1 mmol), 4-Trifluoromethylphenol (0.57 g, 5.1 mmol), and 50 mL of acetonitrile. As cesium carbonate (3.32 g, 10.2 mmol) was added and the mixture was allowed to dissolve, the temperature of the solution rose to 50 °C. After 8 h of stirring, the mixture was allowed to cool to ambient temperature, vacuum-filtered, and then dried using a rotary evaporator. The residue was diluted with 50 mL of water, and then the solution was extracted using ethyl acetate (50 mL × 3). Anhydrous sodium sulfate was mixed with the organic layer and allowed to dry overnight. Vacuum filtering was followed by reduced pressure evaporation of the solvent to provide a crude product (light yellow oil 1.03 g, yield 90.5%) that was used straight away for the next stage without additional purification. ESI-MS *m*/*z*: 226.1 $[M + H]^+$.

Synthesis of 4-(4-(Trifluoromethyl)phenoxy) Butanoic Acid

In a 100 mL tri-necked bottle containing ethyl 4-(4-(trifluoromethyl)phenoxy) butanoate (1.03 g, 4.61 mmol), 20 mL of ethanol was added, and the mixture was shaken until it dissolved. Then, 3 mL of water and NaOH (0.75 g, 18.8 mmol) were added to the bottle at 25 °C, and the solution was refluxed for 2 h. Through the use of thin-layer chromatography (TLC), the reaction was observed. After cooling the mixture to 25 °C, it was dried with a rotary evaporator, and the remaining 30 mL was diluted with water. Then, 1N hydrochloric acid was added to the solution to bring its pH to 5, resulting in an amount of solid white precipitate. After vacuum filtration was employed to dry the precipitate, a crude product (white solid 0.63 g, yield 69.8%) was obtained. This product was used straight away for the following stage without undergoing additional purification. ESI-MS *m*/*z*: 199.3 $[M + H]^+$.

Synthesis of 2-(4-Chlorophenoxy)-N-(2-oxo-1,3-oxazinan-3-yl) Acetamide (**Y-27**)

A mixture of 4-(4-(trifluoromethyl)phenoxy) butanoic acid (0.65 g, 3.22 mmol) and 20 mL of dichloromethane in a 50 mL tri-necked container was swirled until dissolved. Then, EDCI (0.85 g, 4.44 mmol), HOBt (0.6 g, 4.44 mmol), and triethylamine (0.84 g, 9.25 mmol) were added to a bottle at 0 °C. After agitating in an ice bath for 1 h, 3-amino-tetrahydro-l,3-oxazin-2-one (0.37 g, 3.22 mmol) was added again. After heating the mixture to 25 °C, it was stirred overnight. Following TLC, the product underwent rotational evaporation drying after being vacuum-filtered. Column chromatography (CH_2Cl_2: MeOH = 40:1). Compound **Y-27** was obtained as a white solid, 0.71g, with a yield of 55.1%. ^{1}H NMR (600 MHz, DMSO-d_6) δ 10.22 (s, 1H), 7.64 (d, *J* = 8.7 Hz, 2H), 7.11 (d, *J* = 8.6 Hz, 2H), 4.25–4.20 (m, 2H), 4.09 (t, *J* = 6.4 Hz, 2H), 3.43 (t, *J* = 6.2 Hz, 2H), 2.30 (t, *J* = 7.3 Hz, 2H), 2.07–1.94 (m, 4H). ^{13}C NMR (151 MHz, DMSO-d_6) δ 171.10, 161.85, 152.99, 127.43, 127.41, 127.38, 127.35, 125.95, 124.16, 122.37, 121.61, 121.40, 121.19, 115.41, 67.49, 67.29, 67.18, 49.31, 29.84, 24.74, 22.76; ESI-MS *m*/*z*: 347.1140 $[M + H]^+$.

3.1.33. Synthesis of Target Compound **Y-31**

Synthesis of Ethyl 4-(4-Iodophenoxy) Butanoate

A 100 mL three-neck bottle contained ethyl 4-bromobutyrate (1 g, 5.1 mmol), 4-Iodophenol (1.11 g, 5.1 mmol), and 50 mL of acetonitrile. As cesium carbonate (3.32 g, 10.2 mmol) was added and the mixture was allowed to dissolve, the temperature of the solution rose to 50 °C. After 8 h of stirring, the mixture was allowed to cool to ambient temperature, vacuum-filtered, and then dried using a rotary evaporator. The residue was diluted with 50 mL of water, and then the solution was extracted using ethyl acetate (50 mL × 3). Anhydrous sodium sulfate was mixed with the organic layer and allowed to dry overnight. Vacuum filtering was followed by reduced pressure evaporation of the solvent to provide a crude product (light yellow oil 1.61 g, yield 95.1%) that was used straight away for the next stage without additional purification. ESI-MS *m*/*z*: 335.01 $[M + H]^+$.

Synthesis of 4-(4-Iodophenoxy) Butanoic Acid

In a 100 mL tri-necked bottle containing ethyl 4-(4-iodophenoxy) butanoate (1.61 g, 4.85 mmol), 20 mL of ethanol was added, and the mixture was shaken until it dissolved. Then, 3 mL of water and NaOH (0.75 g, 18.8 mmol) were added to the bottle at 25 °C, and the solution was refluxed for 2 h. Through the use of thin-layer chromatography (TLC), the reaction was observed. After cooling the mixture to 25 °C, it was dried with a rotary evaporator, and the remaining 30 mL was diluted with water. Then, 1N hydrochloric acid was added to the solution to bring its pH to 5, resulting in an amount of solid white precipitate. After vacuum filtration was employed to dry the precipitate, a crude product (white solid 1.07 g, yield 72.3%) was obtained. This product was used straight away for the following stage without undergoing additional purification. ESI-MS *m*/*z*: 306.98 $[M + H]^+$.

Synthesis of 4-(4-Iodophenoxy)-N-(2-oxo-1,3-oxazinan-3-yl) Butanamide (**Y-31**)

A mixture of 4-(4-iodophenoxy) butanoic acid (1.07 g, 3.51 mmol) and 20 mL of dichloromethane in a 50 mL tri-necked container was swirled until dissolved. Then, EDCI (0.85 g, 4.44 mmol), HOBt (0.6 g, 4.44 mmol), and triethylamine (0.84 g, 9.25 mmol) were added to a bottle at 0 °C. After agitating in an ice bath for 1 h, 3-amino-tetrahydro-l,3-oxazin-2-one (0.41 g, 3.51 mmol) was added again. After heating the mixture to 25 °C, it was stirred overnight. Following TLC, the product underwent rotational evaporation drying after being vacuum-filtered. Column chromatography (CH_2Cl_2: MeOH = 40:1). Compound **Y-31** was obtained as a white solid, 0.85g, with a yield of 60.1% ^{1}H NMR (600 MHz, DMSO-d_6) δ 10.20 (s, 1H), 7.61–7.55 (m, 2H), 6.81–6.75 (m, 2H), 4.24–4.19 (m, 2H), 3.97 (t, J = 6.4 Hz, 2H), 3.42 (t, J = 6.2 Hz, 2H), 2.27 (t, J = 7.3 Hz, 2H), 2.07–2.00 (m, 2H), 1.95 (p, J = 6.8 Hz, 2H).^{13}C NMR (151 MHz, DMSO-d_6) δ 171.12, 158.89, 152.99, 138.42, 117.76, 117.73, 83.48, 67.21, 67.18, 50.45, 49.31, 29.89, 24.80, 22.77; ESI-MS *m*/*z*: 405.0309 $[M + H]^+$.

3.1.34. Synthesis of Target Compound **Y-32**

Synthesis of Ethyl 2-(4-Nitrophenoxy) Acetate

A 100 mL three-neck bottle contained Ethyl bromoacetate (1.02 g, 6 mmol), 4-Nitrophenol (0.83 g, 6 mmol), and 50 mL of acetonitrile. As cesium carbonate (3.32 g, 10.2 mmol) was added and the mixture was allowed to dissolve, the temperature of the solution rose to 50 °C. After 8 h of stirring, the mixture was allowed to cool to ambient temperature, vacuum-filtered, and then dried using a rotary evaporator. The residue was diluted with 50 mL of water, and then the solution was extracted using ethyl acetate (50 mL × 3). Anhydrous sodium sulfate was mixed with the organic layer and allowed to dry overnight. Vacuum filtering was followed by reduced pressure evaporation of the solvent to provide a crude product (light yellow oil 1.21 g, yield 89.3%) that was used straight away for the next stage without additional purification. ESI-MS *m*/*z*: 226.06 $[M + H]^+$.

Synthesis of 2-(4-Nitrophenoxy) Acetic Acid

In a 100 mL tri-necked bottle containing ethyl 2-(4-nitrophenoxy) acetate (1.21 g, 5.36 mmol), 20 mL of ethanol was added, and the mixture was shaken until it dissolved. Then, 3 mL of water and NaOH (0.75 g, 18.8 mmol) were added to the bottle at 25 °C, and the solution was refluxed for 2 h. Through the use of thin-layer chromatography (TLC), the reaction was observed. After cooling the mixture to 25 °C, it was dried with a rotary evaporator, and the remaining 30 mL was diluted with water. Then, 1N hydrochloric acid was added to the solution to bring its pH to 5, resulting in an amount of solid white precipitate. After vacuum filtration was employed to dry the precipitate, a crude product (white solid 0.79 g, yield 75.3%) was obtained. This product was used straight away for the following stage without undergoing additional purification. ESI-MS *m*/*z*: 199.09 $[M + H]^+$.

Synthesis of 2-(4-Nitrophenoxy)-N-(2-oxo-1,3-oxazinan-3-yl) Acetamide (**Y-32**)

A mixture of 2-(4-nitrophenoxy) acetic acid (0.79 g, 4.03 mmol) and 20 mL of dichloromethane in a 50 mL tri-necked container was swirled until dissolved. Then, EDCI (0.85 g, 4.44 mmol), HOBt (0.6 g, 4.44 mmol), and triethylamine (0.84 g, 9.25 mmol) were added to a bottle at 0 °C. After agitating in an ice bath for 1 h, 3-amino-tetrahydro-l,3-oxazin-2-one (0.47 g, 4.03 mmol) was added again. After heating the mixture to 25 °C, it was stirred overnight. Following TLC, the product underwent rotational evaporation drying after being vacuum-filtered. Column chromatography (CH_2Cl_2: MeOH = 40:1). Compound Y-32 was obtained as a white solid, 0.67g, with a yield of 56.2%. ^1H NMR (600 MHz, DMSO-d_6) δ 10.62 (s, 1H), 8.26–8.17 (m, 2H), 7.22–7.16 (m, 2H), 4.81 (s, 2H), 4.27–4.22 (m, 2H), 3.47 (t, *J* = 6.2 Hz, 2H), 2.10–2.03 (m, 2H).^{13}C NMR (151 MHz, DMSO-d_6) δ 171.12, 158.89, 152.99, 138.42, 117.76, 117.73, 83.48, 67.21, 67.18, 50.45, 49.31, 40.55, 29.89, 24.80, 22.77; ESI-MS *m*/*z*: 296.0879 $[M + H]^+$.

3.1.35. Synthesis of 4-Chloro-N-(2-oxo-1,3-oxazinan-3-yl) Benzamide **Y-34**

A mixture of 4-Chlorobenzoic acid (0.5 g, 3.19 mmol) and 20 mL of dichloromethane in a 50 mL tri-necked container was swirled until dissolved. Then, EDCI (0.85 g, 4.44 mmol), HOBt (0.6 g, 4.44 mmol), and triethylamine (0.84 g, 9.25 mmol) were added to a bottle at 0 °C. After agitating in an ice bath for 1 h, 3-amino-tetrahydro-l,3-oxazin-2-one (0.31g, 3.19mmol) was added again. After heating the mixture to 25°C, it was stirred overnight. Following TLC, the product underwent rotational evaporation drying after being vacuum-filtered. Column chromatography (CH_2Cl_2: MeOH = 40:1). White solid 0.61 g. Yield of 75.29%. ^1H NMR (600 MHz, DMSO-d_6) δ 10.93 (s, 1H), 7.90–7.86 (m, 2H), 7.62–7.58 (m, 2H), 4.29 (t, *J* = 5.3 Hz, 2H), 3.55 (t, *J* = 6.2 Hz, 2H), 2.14–2.07 (m, 2H). ^{13}C NMR (151 MHz, DMSO-d_6) δ 164.62, 153.03, 137.45, 131.24, 129.86, 129.86, 129.17, 129.17, 67.36, 49.37, 22.85; ESI-MS *m*/*z*: 255.0534 $[M + H]^+$.

3.1.36. Synthesis of Target Compound **Y-35**

Synthesis of Ethyl 4-Phenoxybutanoate

A 100 mL three-neck bottle contained ethyl 4-bromobutyrate (1 g, 5.1 mmol), phenol (0.77 g, 5.1 mmol), and 50 mL of acetonitrile. As cesium carbonate (3.32 g, 10.2 mmol) was added and the mixture was allowed to dissolve, the temperature of the solution rose to 50 °C. After 8 h of stirring, the mixture was allowed to cool to ambient temperature, vacuum-filtered, and then dried using a rotary evaporator. The residue was diluted with 50 mL of water, and then the solution was extracted using ethyl acetate (50 mL × 3). Anhydrous sodium sulfate was mixed with the organic layer and allowed to dry overnight. Vacuum filtering was followed by reduced pressure evaporation of the solvent to provide a crude product (light yellow oil 0.99

g, yield 93.8%) that was used straight away for the next stage without additional purification. ESI-MS *m*/*z*: 209.11 [M + H]$^+$.

Synthesis of 4-Phenoxybutanoic Acid

In a 100 mL tri-necked bottle containing ethyl 4-phenoxybutanoate (0.99 g, 4.78 mmol), 20 mL of ethanol was added, and the mixture was shaken until it dissolved. Then, 3 mL of water and NaOH (0.75 g, 18.8 mmol) were added to the bottle at 25 °C, and the solution was refluxed for 2 h. Through the use of thin-layer chromatography (TLC), the reaction was observed. After cooling the mixture to 25 °C, it was dried with a rotary evaporator, and the remaining 30 mL was diluted with water. Then, 1N hydrochloric acid was added to the solution to bring its pH to 5, resulting in an amount of solid white precipitate. After vacuum filtration was employed to dry the precipitate, a crude product (white solid 0.62 g, yield 72.5%) was obtained. This product was used straight away for the following stage without undergoing additional purification. ESI-MS *m*/*z*: 181.08 [M + H]$^+$.

Synthesis of N-(2-Oxo-1,3-oxazinan-3-yl)-4-phenoxybutanamide (**Y-35**)

A mixture of 4-phenoxybutanoic acid (0.62 g, 3.46 mmol) and 20 mL of dichloromethane in a 50 mL tri-necked container was swirled until dissolved. Then, EDCI (0.85 g, 4.44 mmol), HOBt (0.6 g, 4.44 mmol), and triethylamine (0.84 g, 9.25 mmol) were added to a bottle at 0 °C. After agitating in an ice bath for 1 h, 3-amino-tetrahydro-l,3-oxazin-2-one (0.41g, 3.46 mmol) was added again. After heating the mixture to 25 °C, it was stirred overnight. Following TLC, the product underwent rotational evaporation drying after being vacuum-filtered. Column chromatography (CH_2Cl_2: MeOH = 40:1). Compound **Y-35** was obtained as a white solid, 0.65g, with a yield of 58.2%. ^{1}H NMR (600 MHz, DMSO-d_6) δ 10.20 (s, 1H), 7.31–7.24 (m, 2H), 6.94–6.89 (m, 3H), 4.24–4.19 (m, 2H), 3.98 (t, *J* = 6.4 Hz, 2H), 3.43 (t, *J* = 6.2 Hz, 2H), 2.28 (t, *J* = 7.4 Hz, 2H), 2.07–2.00 (m, 2H), 1.96 (p, *J* = 6.9 Hz, 2H). ^{13}C NMR (151 MHz, DMSO-d_6) δ 171.20, 158.97, 153.00, 129.94, 129.94, 120.94, 114.91, 114.88, 67.18, 66.89, 49.31, 29.98, 24.95, 22.77; ESI-MS *m*/*z*: 279.1381 [M + H]$^+$.
