# Supplementary material for: Design, Synthesis, and Biological Evaluation of the Quorum-Sensing Inhibitors of Pseudomonas aeruginosa PAO1
Source: Molecules. 2024 May 8;29(10):2211. doi: 10.3390/molecules29102211 (PMC11123961; doi:10.3390/molecules29102211)
Supplement: Supplementary file 1 [file molecules-29-02211-s001.zip › Supplemntary Table S1.pdf]

Supplemental information

Design, synthesis, and biological evaluation of the quorum-sensing inhibitors of *Pseudomonas aeruginosa* PAO1

1 National Engineering Research Center for the Emergency Drug, State Key Laboratory of Toxicology and Medical Countermeasures, Beijing Institute of Pharmacology and Toxicology, Beijing 100850, China; yanxinlin11@163.com (X.Y.) houshi28@126.com (S.H.); jiajiachang@126.com (J.C.);
2 School of Pharmaceutical Sciences, Jilin University, Changchun 130021, China; xing-cheng21@mails.jlu.edu.cn (C.X.)
3 School of Life Sciences, Jilin University, Changchun, 130012, China; yuanyuanz21@mails.jlu.edu.cn (Y.Z)
* Correspondence: xiaojunhai@139.com (J.X); Tel.:01066931634

Table S1 Inhibition rate of biofilm by different compounds at different concentrations

	Inhibition ratio							
concentration	15	25	26	29	31	37	39	C_{10}-HSL
325	0.3207	0.3217	0.3869	0.0907	0.4098	0.1816	0.2124	-0.1877
162.5	0.3385	0.3987	0.4457	0.4091	0.4213	0.2869	0.2487	0.063
81.25	0.3680	0.3335	0.4249	0.3466	0.3954	0.3644	0.2841	0.0025
40.625	0.2704	0.3219	0.3345	0.3273	0.4044	0.3515	0.2342	0.0225
20.3125	0.06725	0.18803	0.02166	0.09513	0.211	0.1527	0.1287	-0.0435
